# The 2023 AO Spine-Praxis Guidelines in Acute Spinal Cord Injury: What Have We Learned? What Are the Critical Knowledge Gaps and Barriers to Implementation?

**DOI:** 10.1177/21925682231196825

**Published:** 2024-03-25

**Authors:** Michael G. Fehlings, Ali Moghaddamjou, Nathan Evaniew, Lindsay A. Tetreault, Mohammed Ali Alvi, Andrea C. Skelly, Brian K. Kwon

**Affiliations:** 1 Division of Neurosurgery and Spine Program, Department of Surgery, 7938 University of Toronto, Toronto, ON, Canada; 2 Division of Neurosurgery, Krembil Neuroscience Centre, Toronto Western Hospital, 7989 University Health Network, Toronto, ON, Canada; 3 Institute of Medical Science, 7938 University of Toronto, Toronto, ON, Canada; 4 McCaig Institute for Bone and Joint Health, Department of Surgery, Orthopaedic Surgery, Cumming School of Medicine, 2129 University of Calgary, AB, Canada; 5 Department of Neurology, NYU Langone Medical Center, New York, NY, USA; 6 Aggregate Analytics, Inc, Fircrest, WA, USA; 7 Department of Orthopaedics, 8166 University of British Columbia, Vancouver, BC, Canada; 8 International Collaboration on Repair Discoveries (ICORD), 8166 University of British Columbia, Vancouver, BC, Canada

**Keywords:** spinal cord injury, hemodynamic management, trauma, timing of surgery, decompression, timing of surgery, intraoperative SCI

## Abstract

**Study Design:**

Narrative summary of the 2023 AO Spine-Praxis clinical practice guidelines for management in acute spinal cord injury (SCI).

**Objectives:**

The objective of this article is to summarize the key findings of the clinical practice guidelines for the optimal management of traumatic and intraoperative SCI (ISCI). This article will also highlight potential knowledge translation opportunities for each recommendation and discuss important knowledge gaps and areas of future research.

**Methods:**

Systematic reviews were conducted according to accepted methodological standards to evaluate the current body of evidence and inform the guideline development process. The summarized evidence was reviewed by a multidisciplinary guidelines development group that consisted of international multidisciplinary stakeholders. The Grading of Recommendation, Assessment, Development, and Evaluation (GRADE) approach was used to rate the certainty of the evidence for each critical outcome and the “evidence to recommendation” framework was used to formulate the final recommendations.

**Results:**

The key recommendations regarding the timing of surgical decompression, hemodynamic management, and the prevention, diagnosis, and management of ISCI are summarized. While a strong recommendation was made for early surgery, further prospective research is required to define what constitutes sufficient surgical decompression, examine the role of ultra-early surgery, and assess the impact of early surgery in different SCI phenotypes, including central cord syndrome. Furthermore, additional investigation is required to evaluate the impact of mean arterial blood pressure targets on neurological recovery and to determine the utility of spinal cord perfusion pressure measurements. Finally, there is a need to examine the role of neuroprotective agents for the treatment of ISCI and to prospectively validate the new AO Spine-Praxis care pathway for the prevention, diagnosis, and management of ISCI. To optimize the translation of these guidelines into practice, important barriers to their implementation, particularly in underserved areas, need to be explored. Ultimately, these recommendations will help to establish more personalized approaches to care for SCI patients.

**Conclusions:**

The recommendations from the 2023 AO Spine-Praxis guidelines not only highlight the current best practice in the management of SCI, but reveal critical knowledge gaps and barriers to implementation that will help to guide further research efforts in SCI.

## Introduction

Spinal Cord Injury (SCI) is a devastating life-altering condition that imposes a significant burden on patients, their families, and the global healthcare system. Despite the impact of SCI, treatment of this condition remains challenging. Guidelines for the management of SCI have been summarized in this focus issue in the Global Spine Journal. Three specific topics were addressed: a) the role and timing of surgical intervention for acute SCI; b) the optimal hemodynamic management of acute SCI; and c) the prevention, diagnosis and management of intraoperative SCI (ISCI). The first topic represents an update from the 2017 AO Spine Guidelines,^1^ which was deemed necessary given the emergence of several new high-quality studies.^2-11^ The second topic is an update of the 2013 AANS/CNS guidelines,^12^ which was deemed a priority due to the uncertainty in the field regarding the optimum blood pressure parameters to use for hemodynamic management of acute SCI, and the growing interest in the measurement of spinal cord perfusion pressure (SCPP).^13-15^ The third topic, on the diagnosis and management of ISCI, represents an entirely novel guideline effort and fills a significant knowledge gap in the literature.^16^

To develop these guidelines, we utilized rigorous methodology that abided by current standards.^17^ In the first step, a summary of the evidence was developed through systematic reviews of the literature using the PRISMA standards. All systematic reviews were registered on PROSPERO. In the second step, the results of the systematic reviews were reviewed by a multidisciplinary, international guideline development group (GDG) to formulate recommendations based on the best available evidence. In the case of defining ISCI and summarizing its incidence and risk factors, both a systematic review and a scoping review were conducted for rigor and completeness.

The well-established Grading of Recommendation, Assessment, Development, and Evaluation (GRADE) system was used to rate the overall quality of evidence and assist in guideline development.^18^ GRADE methodology allows for a systematic approach to achieve consensus by considering the certainty of the evidence for benefits and harms, patient values, resource use, impact on health inequities, and the acceptability and feasibility of various treatment options. To accurately reflect the viewpoints of all stakeholders, the GDG included spine and trauma surgeons, neurologists, rehabilitation specialists, critical and neurocritical care physicians, and individuals with lived experience. In addition, an effort was made to recruit specialists from across the world to incorporate international perspectives. Considerable consultation occurred with various key stakeholders and external societies.

The current article summarizes the key findings of the guidelines and emphasizes practical considerations. Importantly, knowledge gaps and areas for future research are discussed, as are unique challenges related to knowledge translation and implementation.

## Role and Timing of Surgery for Acute Spinal Cord Injury

With respect to the timing of surgery, the GDG made a strong recommendation that surgical decompression be completed within 24 hours after injury when medically feasible.^19^ The ability to generate a stronger recommendation than the AO Spine guidelines of 2017 was in part related to a 2021 meta-analysis that combined the results of 4 large datasets with over 1500 patients to conclude that ASIA Impairment Scale (AIS) grade conversion and ASIA motor score improvement were higher in those who were decompressed within 24 hours.^2^

While these guidelines provide a strong recommendation regarding the timing of surgery, the process of evaluating the evidence using the GRADE system has revealed the need for additional research to further define the effect of surgical timing on outcomes after acute SCI. One of the key questions that remains unanswered is related to the definition and potential efficacy of ultra-early surgery. It is readily acknowledged that there is nothing “biologically magical” about the 24 hour time point after an acute SCI. This cut-off emerged from the STASCIS study as an approximate median time from injury to surgical decompression and also represented a medically feasible and logistically realistic time window to try and accomplish a surgical decompression.^20^ Without question, there is a temporal pattern to the pathophysiologic responses that occur after SCI, which provides a biological rationale for why a decompression even earlier than 24 hours post-injury would be better for the spinal cord. However, based on the current level of evidence, the GDG could not make a definitive recommendation regarding what might be considered an ultra-early decompression or the impact of earlier time thresholds (ie <4, <8 hours) on neurological recovery. First, the literature provides inconsistent definitions of what actually constitutes ultra-early.^21-24^ Furthermore, timing of surgery following injury is sometimes confused with timing of surgery following arrival to the hospital. To facilitate data pooling, it is recommended that future studies adopt consistent definitions for ultra-early surgery. Furthermore, post-hoc analyses using advanced analytical techniques can treat time between injury to surgical decompression as a continuous variable, which may provide valuable insights. Indeed, such an approach has previously suggested a dose-response relationship between the timing of surgical intervention and neurological recovery.^2^

A key priority to enhance the assessment of the impact of surgical decompression (or any other acute therapeutic intervention for that matter) on outcomes after acute SCI is to establish improved techniques for evaluating the nature of the SCI and neurological function. One of the key confounding issues in interpreting ultra-early surgical decompression is accounting for the effect of ultra-early baseline neurologic assessment. Specifically, ultra-early surgical decompression implies that the baseline neurological assessment was also performed ultra-early. This is inherently a more challenging examination, making it more difficult to interpret neurological benefit from an intervention such as surgery. At a minimum, rigorous international training in ISNCSCI standards would be helpful for enhancing the reliability of the initial neurological examination,^25^ and this would likely reduce inconsistencies as well as improve data analysis (particularly when trying to pool large datasets from multiple sources). This could represent a key knowledge translation opportunity for groups such as AO Spine and other societies such as ASIA, whose InSTeP initiative has made training of the International Standards accessible worldwide.^26^

Moreover, accepting that there are inherent limitations to the functional neurological examination in the early injury setting (particularly with patients who have concomitant injuries and are unable to voluntarily participate in the examination), there is an urgent need to develop alternative objective tools for assessing acute SCI. Ongoing work in this area is trying to establish biomarkers through neurophysiology,^27^ imaging,^28,29^ or biochemical/molecular analysis of serum or cerebrospinal fluid (CSF).^30-32^

The impact of early surgical decompression also needs to be explored across different SCI phenotypes. Of particular importance is central cord syndrome (CCS), which is the most common cause of incomplete SCI.^33^ Despite the importance of CCS, a limited number of studies have addressed the timing of surgery in this cohort of patients. Until prospective research is conducted on this population, the benefits of early surgical decompression seen in SCI must be extrapolated to CCS.^34^ Of note, recent studies were published after the systematic review was completed for the current focus issue and provide evidence that early surgical intervention within 24 hours is beneficial for patients with central cord injury and incomplete cervical SCI.^34,35^ one of the main challenges in individuals with very “mild” incomplete cervical SCI, where the motor impairment may be modest, is determining the effect of surgical decompression on other important (and often overlooked) features such as spasticity, balance, and neuropathic pain. Through the AO Spine Knowledge Forum in SCI, an effort is ongoing to better study patients with incomplete cervical SCI and other important impairments beyond motor weakness.

Another topic that merits further exploration relates to whether the completeness of decompression influences outcomes after acute SCI. The work of Aarabi and colleagues, in which the completeness of decompression after cervical SCI impacted outcomes, is interesting and merits further careful prospective evaluation.^36^ Moreover, the role of expansile duroplasty^37^ in enhancing neurological outcomes after acute SCI merits further investigation and will partly be addressed by the trial being conducted by Papadopoulos and colleagues in the United Kingdom (NCT04936620). In this regard, the use of intraoperative ultrasound to assess the completeness of decompression in the setting of acute SCI also warrants further evaluation.^38^

Since the completion of the systematic review and guideline development process, the highly anticipated SCI Prospective Observational European Multicenter comparative cohort study (SCI-POEM) has been published.^39^ Unfortunately, the SCI-POEM study has numerous methodological issues which detract from any conclusions which might be drawn from the work. The 12 hour cut-off employed in the study would be categorized as ultra-early surgery and not as early intervention, as reported by the authors. Interestingly, it appears that a significant proportion of patients classified in the “late” group in the study did, in fact, undergo early surgery utilizing the internationally accepted and validated 24 hour cut-off to define “early” surgery. Moreover, the SCI-POEM study would not have impacted our conclusions or recommendations regarding ultra-early surgery, owing to several critical limitations. Indeed these issues would have likely resulted in an assessment of “very low quality” using the rigorous GRADE standards. These critical limitations include the presence of considerable residual imbalances in the 2 study cohorts (ultra-early vs delayed intervention) despite extensive statistical manipulation, high rates of loss-to-follow-up, lack of statistical power to model the outcomes separately in cervical and thoracic SCI cases, and the absence of an assessment of upper extremity motor function, which is a key priority for individuals with cervical SCI.

## Hemodynamic Management

Regarding the hemodynamic management of acute SCI, the GDG acknowledged the very low quality of available evidence and formulated weak recommendations that mean arterial pressure (MAP) be maintained between 75-80 mmHg on the low end to between 90-95 on the upper end for between 3 to 7 days post-injury. No conclusions were made regarding the use of a specific vasopressor or inotrope due to the current level of evidence. While this does represent a departure from the 2013 AANS/CNS guidelines which recommended that MAP be maintained between 85-90 mmHg for 7 days,^40^ the GDG agreed that the available literature did not consistently support a neurological benefit with this range of 85-90 mmHg, and there was little evidence to substantiate a 7 day duration of treatment for all patients. Hence, these new guidelines, to some extent, represent an acknowledgement of the uncertainty that remains in the field around the neurological effects of specific MAP targets. They also highlight the need for high quality prospective studies to better delineate the relationship between MAP targets and neurological outcomes. As institutions adopt electronic medical records and have the ability to collect high-frequency physiologic and hemodynamic data as the UCSF group has done for many years, it will likely be possible to access such large datasets and utilize advanced analytical techniques to better discern the relationship between specific MAP targets and neurological outcome.^41^ The challenge for the future is in going beyond “associations” and “correlations” to actual “causation” when interpreting the role of MAP augmentation in acute SCI care.

Of course, beyond the MAP is the emerging interest in SCPP. This concept of SCPP is, of course, not foreign to the neurosurgical community, which has for years focused on cerebral perfusion pressure (CPP) in traumatic brain injury as the difference between MAP and intracranial pressure (ICP). The GDG agreed that the evidence was not sufficient to establish a guideline around SCPP, and that a recommendation would not be practical given that only a handful of centers are actively studying this parameter. One of the key knowledge gaps for future research will be to understand the differences between measuring intrathecal/CSF pressure at the site of SCI with a pressure catheter inserted locally at the time of surgery (as described by Papadopoulos and colleagues)^42^ vs distal to the SCI in the lumbar cistern with a pressure/drainage catheter system that is familiar to most clinicians.^43-46^ Elegant work by Papadopoulos and colleagues has already demonstrated that when the injured spinal cord swells against the dura, the “intraspinal” pressure measured at the injury site may be significantly higher than that of the “intrathecal” pressure in the lumbar cistern.^47^ Given the increased understanding of the role of spinal cord swelling and occlusion of the subarachnoid space when the injured spinal cord abuts against the dura (potentially causing an increase in intraspinal pressure), it is apparent that the technique for achieving “complete decompression” (as described above)^48^ is a consideration that is closely related to this aspect of hemodynamic management. As lumbar CSF drainage is routinely performed for a host of indications, including augmenting SCPP in thoracoabdominal aortic aneurysm surgery patients, it will be important in the future to determine if this can be “translated” to the acute SCI population, recognizing the complexities of spinal cord swelling and the potential occlusion of the subarachnoid space at the site of injury. Such investigations are ongoing.

Finally, what is ultimately required is a way of monitoring physiologic responses at the injury site itself. This knowledge gap was emphasized by the aptly titled review by Saadoun and Papadopoulos in 2016: *“Spinal Cord Injury: is monitoring from the injury site the future?”.*^15^ Work by this group has revealed that intradural monitoring of the physiology and metabolism of the injury site in response to hemodynamic management is possible and can yield important information about what is actually happening within the spinal cord after injury. Efforts to establish similar approaches with epidural technologies are ongoing using near-infrared spectroscopy which can non-invasively interrogate the spinal cord tissue with near-infrared light.^49,50^ The technologies that emerge as effective at monitoring this space will fill an important knowledge gap in the hemodynamic management of acute SCI.

## Intraoperative Spinal Cord Injury

Although relatively uncommon, ISCI is an inherent risk to any spine surgery and can have devastating physical and psychological consequences for patients and their families. The current guidelines pertaining to ISCI aim to unify the definition of ISCI, delineate risk factors of ISCI, define the diagnostic accuracy of intraoperative neuromonitoring (IONM) techniques, and propose treatment algorithms for the management of potential ISCI. In terms of the role of IONM, after a systematic review of the literature^51^ the GDG determined that there was sufficient high-quality evidence to recommend considering the use of IONM for high-risk patients undergoing spine surgery. Through a scoping review, we identified the following risk factors for ISCI: 1) complex spine deformity including a rigid thoracic curve with high deformity angular ratio (dAR); 2) revision congenital spine deformity; 3) spine conditions associated with significant cord compression and myelopathy; 4) intramedullary spinal cord tumor; 5) unstable spine fractures including those with bilateral facet dislocation and disc herniation or extension distraction injury with ankylosing spondylitis; and 6) ossification of the posterior longitudinal ligament (OPLL) associated with severe cord compression and moderate to severe myelopathy. The current guidelines also proposed a 5-step systematic approach to the management of ISCI. The steps include initial clinical assessment to identify patient risk, pre-operative planning, intraoperative planning, intraoperative management including anesthesiologic, neurophysiological/technical and surgical strategies, as well as postoperative management.

Given the state of the available literature, there are key knowledge gaps which represent opportunities for further research. Furthermore, implementation of this algorithm for the management of ISCI, of course, represents a significant challenge, and prospective evaluation of the proposed approach will be necessary. Such research could drive further precision in the management and prevention of ISCI. We anticipate that one of the major challenges will be determining what constitutes a “high risk” patient and discerning the role of IONM in this population. For example, should all patients with degenerative cervical myelopathy (who inherently have extrinsic cord compression) have IONM during their surgical decompression? Will this be cost-effective and feasible for all centers, particularly (but not exclusively) in lower income countries? It is important to highlight that these recommendations (especially given they are based on relatively low-quality evidence) should not be used to put surgeons in medicolegal jeopardy, particularly when it is not feasible for them to institute IONM for every case. The GDG did, however, consider that, in the realm of “reducing inequity” (a factor within the GRADE evidence-to-decision framework), a recommendation for IONM could be used by surgeons in resource-limited environments to advocate for this technology in their patients. Studies that specifically look at implementation and cost-effectiveness of IONM strategies will be needed in the future.

## Healthcare Resources

Effective implementation of recommendations made in the current clinical practice guidelines requires a healthcare system capable of transferring patients to facilities equipped to offer acute surgical care. In a 2022 AO Spine survey of spine care professionals, the majority of surgeons reported that they encountered logistical or administrative barriers when attempting early surgical intervention. In the same survey, surgeons from low-middle-income countries (LMICs) were significantly less likely to be able to provide hemodynamic management when compared to surgeons from high-income countries. Factors such as challenges in patient transfer, medical stabilization, diagnostic evaluation, surgeon availability, and operating room resources can hinder timely access to surgical intervention. With these strong recommendations for early surgery, it is crucial to conduct research and local quality improvement initiatives to identify specific barriers to implementation. The current evidence should encourage healthcare systems to streamline care pathways that begin at the site of injury in order to better address barriers to surgical care delivery. These barriers may extend beyond available resources and encompass issues of equity, diversity, and inclusion. Delays in care may arise due to a patient’s socioeconomic status and geographical location. To overcome these obstacles, the first step is to define the scope and impact of the identified barriers in order to bring these issues to the attention of key stakeholders in the healthcare system.

The barriers in accessing the recommended care for SCI are amplified in medically underserved areas and LMICs. A recent meta-analysis revealed that despite the increasing incidence of SCI in LMICs, patients in LMICs had significantly longer times to surgical decompression compared to individuals in high-income countries. These results highlight the global disparities in delivering surgical care for SCI patients. Ensuring fair and equitable access to surgical care in LMICs is a complex issue that requires a multifaceted approach. It is critical to identify the factors that impede access to care and address those that can be easily modified, including surgeons’ perceptions regarding the benefits of early surgery, which may be different in LMICs.

## Personalized Approach

Throughout the guideline development process, the importance of personalizing the approach to individual patient care was highlighted. This is particularly important given the heterogeneity in patient presentations and recovery trajectories seen in SCI. As such, a personalized approach that may deviate from the current recommendations may be justified. With advances in research, the hope is to develop enhanced personalized recommendations in the next iteration of the guidelines.

A personalized approach to care can be provided in every step of the management of SCI. Evaluation of a patient’s physiological reserve by evaluating their frailty and co-morbidities enables better prediction of the clinical trajectory and should be accounted for in his or her management. A personalized approach to surgical decompression should also be considered. Pre- and post-operative MRI sequences can guide management through quantifying the degree of compression of the spinal cord. Pressure monitoring of the injured cord during surgery can also be done to ensure adequate decompression. The use of genetic and serological biomarkers could also further add the precision of SCI management.

With the aging of the population in many regions of the world, managing acute SCI in a safe manner is a real issue that may represent a challenge for clinicians. Care pathways are required for the acute management of elderly individuals and those with significant medical co-morbidities with acute SCI. This represents a challenge to be addressed in future research studies.

A personalized approach is also necessary to enhance the safety of complex spinal surgery. The development of clinical prediction tools to assess the relative risk of an ISCI would augment the judgment and knowledge of experienced clinicians. Moreover, further work is required to prospectively evaluate techniques and approaches for detecting and managing changes in intraoperative neurophysiological monitoring. We hope that the care pathways and approaches developed in the 2023 AO Spine-Praxis guidelines process will enhance the safety of complex spinal surgery.

In summary, the current AO Spine-Praxis guidelines for the management of acute SCI provide a new recommendation related to the optimal timing of surgery for acute SCI, revised MAP goals for the hemodynamic management of acute SCI, and a practical approach to prevent, diagnose and manage ISCI. We hope that the care pathways and approaches summarized in the 2023 AO Spine-Praxis guidelines process will enhance the safety of complex spinal surgery and optimize outcomes for individuals with acute SCI. Finally, we acknowledge that these are living documents that will require updating and revision as new knowledge is generated. We hope that highlighting key knowledge gaps will help stimulate research efforts in important areas to enhance the care and outcomes of patients with SCI.

## Supplemental Material

Supplemental Material - The 2023 AO Spine-Praxis Guidelines in Acute Spinal Cord Injury: What Have We Learned? What Are the Critical Knowledge Gaps and Barriers to Implementation? Supplemental Material for The 2023 AO Spine-Praxis Guidelines in Acute Spinal Cord Injury: What Have We Learned? What Are the Critical Knowledge Gaps and Barriers to Implementation? by Michael G. Fehlings, Ali Moghaddamjou, Nathan Evaniew, Lindsay A. Tetreault, Mohammed Ali Alvi, Andrea C. Skelly, and Brian K. Kwon in Global Spine Journal

Supplemental Material - The 2023 AO Spine-Praxis Guidelines in Acute Spinal Cord Injury: What Have We Learned? What Are the Critical Knowledge Gaps and Barriers to Implementation? Supplemental Material for The 2023 AO Spine-Praxis Guidelines in Acute Spinal Cord Injury: What Have We Learned? What Are the Critical Knowledge Gaps and Barriers to Implementation? by Michael G. Fehlings, Ali Moghaddamjou, Nathan Evaniew, Lindsay A. Tetreault, Mohammed Ali Alvi, Andrea C. Skelly, and Brian K. Kwon in Global Spine Journal

Supplemental Material - The 2023 AO Spine-Praxis Guidelines in Acute Spinal Cord Injury: What Have We Learned? What Are the Critical Knowledge Gaps and Barriers to Implementation? Supplemental Material for The 2023 AO Spine-Praxis Guidelines in Acute Spinal Cord Injury: What Have We Learned? What Are the Critical Knowledge Gaps and Barriers to Implementation? by Michael G. Fehlings, Ali Moghaddamjou, Nathan Evaniew, Lindsay A. Tetreault, Mohammed Ali Alvi, Andrea C. Skelly, and Brian K. Kwon in Global Spine Journal

Supplemental Material - The 2023 AO Spine-Praxis Guidelines in Acute Spinal Cord Injury: What Have We Learned? What Are the Critical Knowledge Gaps and Barriers to Implementation? Supplemental Material for The 2023 AO Spine-Praxis Guidelines in Acute Spinal Cord Injury: What Have We Learned? What Are the Critical Knowledge Gaps and Barriers to Implementation? by Michael G. Fehlings, Ali Moghaddamjou, Nathan Evaniew, Lindsay A. Tetreault, Mohammed Ali Alvi, Andrea C. Skelly, and Brian K. Kwon in Global Spine Journal

Supplemental Material - The 2023 AO Spine-Praxis Guidelines in Acute Spinal Cord Injury: What Have We Learned? What Are the Critical Knowledge Gaps and Barriers to Implementation? Supplemental Material for The 2023 AO Spine-Praxis Guidelines in Acute Spinal Cord Injury: What Have We Learned? What Are the Critical Knowledge Gaps and Barriers to Implementation? by Michael G. Fehlings, Ali Moghaddamjou, Nathan Evaniew, Lindsay A. Tetreault, Mohammed Ali Alvi, Andrea C. Skelly, and Brian K. Kwon in Global Spine Journal
