# Supplementary material for: The 2023 AO Spine-Praxis Guidelines in Acute Spinal Cord Injury: What Have We Learned? What Are the Critical Knowledge Gaps and Barriers to Implementation?
Source: Global Spine J. 2024 Mar 25;14(3 Suppl):223S–230S. doi: 10.1177/21925682231196825 (PMC10964887; doi:10.1177/21925682231196825)
Supplement: Supplemental Material - The 2023 AO Spine-Praxis Guidelines in Acute Spinal Cord Injury: What Have We Learned? What Are the Critical Knowledge Gaps and Barriers to Implementation? [file sj-pdf-1-gsj-10.1177_21925682231196825.pdf]

Definition, Frequency and Risk Factors for Intra-Operative Spinal Cord Injury: A Knowledge Synthesis

Journal:	*Global Spine Journal*
Manuscript ID	Draft
Manuscript Type:	Special Issue- Knowledge Forum SCI Guidelines
Keywords:	spinal cord injury, neuro, trauma

Abstract:

Study Design
Scoping review and systematic review

Objectives
Intra-operative spinal cord injury (ISCI) is a devastating complication of spine surgery. Presently, a uniform definition for ISCI does not exist. Consequently, the reported frequency of ISCI, and important risk factors vary in the existing literature. To address these gaps in knowledge, a two-part knowledge synthesis was designed.

Methods
A scoping review was conducted to review the definitions used for ISCI and ascertain the frequency of ISCI. The definition formed underwent review and voting by the Guidelines Development Group (GDG). A systematic review of the literature was conducted and reviewed by the GDG to determine the risk factors for ISCI.

Results
Frequency of ISCI ranged from 0 to 61%. Older age, male sex, hypertension, combined myelopathy, blood loss, ponte-osteotomy, coronal deformity angular ratio, and curve magnitude were, had a significant increased risk of ISCI. Better pre-operative neurological status, and use of IONM were associated with a significant decreased risk of ISCI. GDG suggested high-risk factors were, rigid thoracic curve with high deformity angular ratio, revision congenital deformity with significant cord compression and myelopathy, extrinsic intradural or extradural Lesion with cord compression and myelopathy, intramedullary tumor, unstable fractures (bilateral facet dislocation and disc herniation, extension distraction injury with ankylosing spondylitis, ossification of posterior longitudinal ligament (OPLL) with severe cord compression and moderate to severe myelopathy...

SCHOLARONE™
Manuscripts

ABSTRACT

Study Design

Scoping review and systematic review.

Objectives

Intra-operative spinal cord injury (ISCI) is a devastating complication of spine surgery. Presently, a uniform definition for ISCI does not exist. Consequently, the reported frequency of ISCI, and important risk factors vary in the existing literature. To address these gaps in knowledge, a mixed-methods knowledge synthesis was undertaken.

Methods

A scoping review was conducted to review the definitions used for ISCI and ascertain the frequency of ISCI. The definition of ISCI underwent formal review, revision and voting by the Guidelines Development Group (GDG). A systematic review of the literature was conducted to determine the risk factors for ISCI. Based on this systematic review, a table was created to summarize the factors deemed to increase the risk for ISCI. All reviews were done according to PRISMA standards and were registered on PROSPERO.

Results

The frequency of ISCI ranged from 0 to 61%. Older age, male sex, cardiovascular disease including hypertension, severe myelopathy, blood loss, requirement for osteotomy, coronal deformity angular ratio, and curve magnitude were associated with increased risk of ISCI. Better pre-operative neurological status, and use of IONM were associated with a decreased risk of ISCI. The risk factors for ISCI included a rigid thoracic curve with high deformity angular ratio, revision congenital deformity with significant cord compression and myelopathy, extrinsic intradural or extradural lesions with cord compression and myelopathy, intramedullary spinal cord tumor, unstable spine fractures (bilateral facet dislocation and disc herniation, extension distraction injury with ankylosing spondylitis, ossification of posterior longitudinal ligament (OPLL) with severe cord compression and moderate to severe myelopathy.

Conclusions

For Peer Review

ISCI has been defined as “a new or worsening neurological deficit attributable to spinal cord dysfunction during spine surgery that is diagnosed intra-operatively via neurophysiologic monitoring or by an intraoperative wake-up test, or immediately post-operatively based on clinical assessment”. This paper defines clinical and imaging factors which increase the risk for ISCI, and which could assist clinicians in decision making.

For Peer Review

INTRODUCTION

Intra-operative spinal cord injury (ISCI) is one of the most feared and devastating complications of spine surgery.[1,2] To date, studies have described ISCI using various definitions and diagnostic criteria; hence, the reported frequency, and risk factors of ISCI have also varied in the literature. Part of this variability in definition is attributed to the fact that some studies use findings of intra-operative neuromonitoring (IONM) to define ISCI, while others rely only on post-operative neurological examination findings. The lack of standardized definitions and diagnostic criteria for ISCI is a major challenge in finding solutions to minimize ISCI. Unifying nomenclature and developing diagnostic criteria are essential for accurately quantifying the frequency of ISCI and ascertaining the risk factors associated with it. To the authors' knowledge, there has been no systematic analysis or synthesis of the literature that defines ISCI, examines the frequency and outcomes of ISCI, delineates the role of IONM, and/or reviews the management strategies in the case of ISCI (both with and without the use of IONM). Additionally, the factors that predispose a patient to sustaining an ISCI have been poorly characterized.

To address these gaps in knowledge, a mixed-methods knowledge synthesis was undertaken. A formal systematic review was planned, and a protocol was registered on (PROSPERO CRD42022298841). The original contextual and key questions and the PICOTS (P=Population, I=Intervention, C=Comparators, O=Outcomes, T=Timing, S=Study Design) are found in Table 1. Briefly, the proposed review intended to (I) provide context regarding case definitions and diagnostic criteria of ISCI, and the use and accuracy of IONM, (ii) evaluate the risk factors for development of ISCI and (iii) address key questions related to comparative effectiveness and harms of ISCI management options and (iii). Broad scoping literature searches of published literature along with input from clinical experts from the guideline development group yielded limited substantial evidence to address the original questions or perform a full systematic review, except for the question regarding risk factors and accuracy of IONM. Given this, our original plan was revised to also incorporate scoping reviews that addressed 1) definitions, frequency, and risk factors for ISCI 2) use and accuracy of IONM for diagnosis of ISCI and 3) reported management approaches for ISCI and related harms.

This purpose of this study was to conduct a scoping and systematic review of the literature to address the following key questions:

For Peer Review

Contextual question: What definitions or monitoring thresholds have been used to define and determine ISCI, and what is the reported frequency of ISCI?

Key Question: What are the risk factors for the development of an intra-operative spinal cord injury?

METHODS

The contextual question on definition, and frequency of ISCI was answered by conducting a scoping review. The key question on risk factors of ISCI was addressed by conducting a systematic review. Methods used for the systematic review of ISCI risk factors were in accordance with the Agency for Healthcare Research and Quality's (AHRQ) *Methods Guide for Effectiveness and Comparative Effectiveness Review*.[3] Contextual questions were answered based on the U.S. Preventive Services Task Force methods[4] for contextual questions and based on citations identified via the formal literature search and gray literature.

Criteria for Inclusion/Exclusion of Studies in the Review

The criteria for inclusion and exclusion of studies for the ***systematic review of risk factors*** for ISCI are specified in Table 1

Study Design: Consistent with other reviews that support guideline updates, studies with the least potential for bias using a "best evidence" approach were focused on. Randomized control trials (RCTs) and high-quality prospective comparative cohort studies that controlled for confounding factors and met inclusion criteria were included as the primary evidence source. Risk factor studies that controlled for confounding were considered as the primary evidence base. In the absence of high-quality studies, lower-quality studies (e.g., case series) and narrative reviews were considered.

Literature Search Strategies

Literature Databases: MEDLINE®, and The Cochrane Library were extensively searched. Only studies published in English were included. Figure 1 contains the terms used in the search. Citations from the search were deduplicated and dual-screened for inclusion. In addition, sources

For Peer Review

of gray literature were reviewed, including professional society guidelines, selected pertinent book chapters, and other similar literature, primarily for the contextual question. Citations suggested by the clinical authors and guideline development group were compared against the a priori criteria for inclusion and exclusion. The EndNote library was searched using Key Words "risk".

Publication Date Range: The search included citations from database inception to January 26, 2022.

Hand Searching: Reference lists of included studies, systematic reviews, and pertinent gray literature were also evaluated for relevant studies.

Process for Selecting Studies

For the systematic review on risk factors of ISCI, the pre-established criteria above were used to screen citations (titles and abstracts) identified by the literature search. Any citation deemed not relevant for full-text review was reviewed by a second researcher to assure accuracy and completeness. Potentially eligible citations identified for inclusion by at least one of the reviewers were retrieved for full-text screening. Each full-text article was independently reviewed for eligibility by two team members. Any disagreements were resolved by consensus. Studies excluded after full-text review with reasons for exclusion are listed in Appendix A. For the ***contextual question,*** studies reporting on thresholds used to identify ISCI were selected if they reported on a minimum of three patients and satisfied the population inclusion/exclusion criteria in Table 1.

Data Abstraction and Data Management

After studies were selected for inclusion for the key question on risk factors, standardized data abstraction included the following (at minimum): patient characteristics (age, sex, comorbidities), completeness (American Spinal Cord Injury Association (ASIA) Impairment Score (AIS) and level of SCI, indication for spine surgery, (e.g., scoliosis, tumor),clinical/disease characteristics (e.g., myelopathy), surgical factors (e.g. approach, levels,

For Peer Review

instrumentation),adjunctive treatments (e.g., steroids, vasopressors), and study-related characteristics (e.g., sample size, design, control of confounding, the timing of follow-up). To address the contextual question, the information on IONM definitions or thresholds and the resolution or persistence of neurological deficit was noted from the selected studies. Data was collected on how the included studies determined there was a neurological deficit. This is done using clinical neurological assessment and categorizing the degree of deficit based on a grading system. Commonly used grading systems in research and clinical practice for categorizing the severity of a neurological deficit due to SCI are ASIA grading system, McCormick grade, and Medical Research Council (MRC) grading system. Neurological deficit can also be detected by changes in IONM parameters. These include somatosensory evoked potentials (SSEP) that monitor the integrity of the dorsal column-medial lemniscal pathway, motor evoked potentials (MEP) and transcranial motor evoked potentials (TcMEP) that monitor the integrity of the corticospinal tracts, and electromyography (EMG) that describes the integrity of individual nerves.[5] Importantly, clinical examination and IONM are not mutually exclusive when reporting on ISCI and studies can use either or both methods to document ISCI.

Assessment of Methodological Risk of Bias of Individual Studies

Pre-defined criteria were used to assess the risk of bias of included nonrandomized studies using the Quality in Prognosis Studies (QUIPS) tool for studies evaluating risk factors .[6] Two methodologists independently assessed the risk of bias. Disagreements were resolved by a discussion leading to consensus. Based on the risk of bias assessment, studies were rated as "good," "fair" or "poor" quality based on the criteria in Table 2. The studies that described ISCI frequency were small case series and were not critically appraised. All were of poor quality.

Data Synthesis

The data was qualitatively summarized in tables using ranges, descriptive analysis, and interpretation of the results. Data on the frequency of ISCI were qualitatively synthesized.

For Peer Review

Adjusted odds ratios provided by authors were reported. Clinical and methodological heterogeneity across studies precluded the pooling of studies.

Grading the Strength of Evidence for Major Comparisons and Outcomes

The overall quality (strength) of evidence (SOE) was assessed based on the application of GRADE described in the AHRQ Methods Guide.[3] GRADE guidance related to synthesis of risk factors was used.[7,8] . The strength of evidence was assigned an overall grade of high, moderate, low, or very low. SOE was initially evaluated by one methodologist and reviewed independently by a second for consistency and validity before the final assessment. Disagreements were resolved by consensus. For the systematic review of risk factors, studies were initially considered to be a high quality of evidence. The evidence was downgraded based on the aggregate assessment of risk of bias across studies reporting on the outcome, consistency, imprecision, directness, and publication bias. Strength of evidence was not applied to the results for the contextual question.

RESULTS

Part one of the Contextual Question; What case definitions of ISCI have been used in the included studies? What criteria or thresholds for evaluation and diagnosis have been used?

Definition of Intra-Operative SCI

The following definition was put forth by the Guidelines Development Group (GDG) after reviewing evidence from the scoping review, and subsequently voting and discussing it in depth, in accordance with the Delphi process [9]: “a new or worsening neurological deficit attributable to spinal cord dysfunction during spine surgery that is diagnosed intra-operatively via neurophysiologic monitoring, by an intraoperative wake-up test or immediately post-operatively based on clinical assessment.” Deficits can include dysfunction attributable to injury of the spinal cord, conus medullaris, or cauda equina.

However, studies provided variable definitions and thresholds for diagnosis of ISCI, which are reported in Table 3.

For Peer Review

The search generated several studies which were grouped based on indication for surgery into those on deformity surgery, and those on tumor surgery. As seen in Table 3, the definitions in the deformity (Kato 2018,[10] Fehlings 2018,[11] Lenke 2016[12]) groups were centered on deterioration in lower extremity motor score (LEMS). LEMS in these studies was described using the ASIA grading system. In contrast, the tumor studies defined ISCI based on change in McCormick grade, MRC grading system, or IONM prompts (Harel 2017,[13] Kang 2017,[14] Korn 2015,[15] Lakomin 2017,[16] Sala 2006,[17] Skinner 2005[18]). Each of these aforementioned studies reported the criteria for defining an ISCI as either a change compared to preoperative LEMS/ ASIA score, a LEMS change of 5 points, change in McCormick grade or in the case of neurophysiologic monitoring, a signal that correlated with a post-operative motor deficit. Briefly, these include MEP amplitude changes > 50-80%, SSEP amplitude changes >50-60%, latency prolongation of 10% or 3msec, TcMEP amplitude change >50-80%, muscle threshold >100 volts, EMG amplitude change >50%, sustained bursts/ trains, lack of waveform, or a D-wave decrease in amplitude >50%.

For Peer Review

Frequency of Intra-operative SCI

For ***the second part of the contextual question*** on the frequency of ISCI, a total of 61 studies (N=15,376) were identified that contained information on the frequency of ISCI (Table 4). Criteria for IONM used to identify ISCI varied among studies. The most commonly employed cut-offs for amplitude drop in signal for SSEP and MEP was 50%, reported by 8 of 13 studies on deformity, 11 of 13 studies for patients with spinal tumors, 9 of 16 studies with mixed pathologies, 7 of 11 studies with cervical region pathology, all 3 studies on patients with thoracic spine pathology, and 1 of 3 studies on patient with lumbar spine pathology. Some studies also used an increase in latency of 10% in adjunct with >50% drop in SSEP or MEP to classify an IONM-based alert. Only cases of ISCI due to spine surgery were reported. Those due to post-operative compression (e.g., hematoma), root-level deficits, or arising from surgeries for pathologies not involving the spine (e.g., vascular surgeries) were not reported. Overall new deficits ranged from 0 to 61%; with increasing granularity, it was found that when the studies were divided by pathology/level of surgery, tumor surgery demonstrated a greater range of frequencies of intra-operative deficits (0-61%) compared to deformity surgery (0-17.8%), while studies with mixed pathologies reported an intermediate range of 0-9.4%. The greatest prevalence of neurological deficit was found in lumbar level surgeries (0-28.5%). Deformity-

related ISCI was more likely to resolve with up to 8% of deficits persisting, while 26.9% of tumor patients had persistent deficits. The sample sizes across studies varied from 5 to 2069, so percentages for ISCI should be interpreted cautiously.

Risk factors for ISCI

From 226 citations identified via literature search, a total of six studies were identified that provided data regarding the frequency of ISCI and conducted multivariable analysis, including four studies (Fehlings 2018,[11] Chen 2012,[19] Kim 2021,[20] Romero-Munoz 2019[21]) that were recommended by clinical experts. (Figure 2). An additional surgeon consensus survey (Iyer 2022)[22] was also considered in the literature analysis, as recommended by the Guidelines Development Group. Data abstraction from these studies is summarized in table 5 and 6.

Five studies evaluated the risk of ISCI (i.e., neurological decline) in the immediate postoperative period, with four studies (Fehlings 2018,[11] Chen 2012,[19] Romero-Munoz 2019,[21] Zhang 2017[23]) using change in ASIA grade to assess neurological status and one study (Kim 2021)[20] using the definition of "any new limb, motor, or sensory neurological deficit". The sixth study (Buckland 2018)[24] evaluated risk of IONM alerts, defined as a reduction in amplitude of 50% or more in SSEPs and/or TcMEPs to signal ISCI. Results from the survey of surgeons on risk factors for ISCI was provided for context (Iyer 2022) [22]. Factors evaluated using multivariable analysis are found in table 7 . Factors assessed using univariate level are found in table 8 . Reported ISCI frequency ranged from 0.3% to 25% (7.1% to 25% in postoperative approaches and 0.3% in the study reporting on risk of IONM alert). Summary of effect estimates is summarized in table 9.

Three patient population groups were identified in the included studies: those with deformity, those with various indications for spine surgery and those with degenerative disease.

Evidence for risk factors for neurological deficits in patients with deformities was derived from one good-quality, prospective cohort (Fehlings 2018, N=265)[11] on scoliosis in adults, and one fair-quality, retrospective cohort (Zhang 2017, N=62) [23] in patients with congenital scoliosis (19%), kyphoscoliosis (74%), and kyphosis (7%). Another poor-quality retrospective cohort (N=2210) (Buckland 2018)[24] described risk factors for IONM alerts in adolescent patients with idiopathic scoliosis.

For Peer Review

Studies in patients with deformity found an increased risk of ISCI with older age, higher blood loss, surgical technique related factors like the requirement for osteotomy, and radiographic factors including coronal deformity angular ratio (DAR) and curve magnitude. For patients with "mixed" indications, one good-quality, retrospective cohort (N=316) (Chen 2012)[19] reported on patients with spinal degeneration (35%), tumor (23%), trauma (22%), deformity (16%), and inflammation (4%), while one fair-quality retrospective cohort (N=1282) (Romero-Munoz 2019)[21] reported on patients with spinal degeneration (75%), deformity (18%), fractures (4%), or other rare injuries (4%). In this latter study, authors did not describe the group to which patients with ISCI were compared to. It appears that patients receiving elective surgery who experienced ISCI were compared to those with other causes of SCI. Studies enrolling patients undergoing spine surgery for a variety of indications found that older age, male sex, hypertension, depression, and a higher number of operative spinal levels were associated with ISCI.

One poor quality retrospective cohort (Kim 2021)[20] reported on patients with degeneration and focused solely on patients with ossification of posterior longitudinal ligament (OPLL). . Two studies (Fehlings,[11] Chen[19]) were rated good, two (Zhang 2017,[23] Romero-Munoz 2020[21]) were rated fair, and two (Kim 2021,[20] Buckland 2019[24]) were rated poor quality (Table 10). Common methodological concerns included retrospective collection of complications (five of the six studies were retrospective study designs) and unclear or unknown study attrition. Other, less frequent concerns included inadequate description of inclusion/exclusion criteria and unclear validity and/or reliability of the measurement methods for prognostic factors and/or confounders. For the studies where the indication for surgery was degeneration, male sex, obesity, CCI, combined myelopathy, a higher number of operative spinal levels and increased time of surgery were associated with ISCI.

Patient Specific Risk Factors for ISCI

Five studies reported on age as a risk factor for ISCI (Fehlings 2018, [11] Romero Munoz 2019, [21] Zhang 2017, [23] Chen 2012,[19] Kim 2021[20]). Four studies reported increased risk of ISCI with increased age (Fehlings 2018,[11] Romero Munoz 2019, [21] Zhang 2018[23], Chen 2012 [19]). Two of these studies reported this as a significant association (Zhang 2017, [23] Chen 2012[19]), while in the other two studies the association was not significant (Fehlings 2018, [11] Romero Munoz 2019 [21]). In one study a slightly decreased risk of ISCI was associated with increased age (OR=0.97), that

For Peer Review

was not significant ($p>0.05$) (Kim 2021[20]).Male sex was associated with increased risk of ISCI in two studies. The association was significant in one study, while not significant in the other (Chen 2012, [19] Kim[20]).Hypertension was associated with a significant increased risk of ISCI in one study, and a non-significant increased risk of ISCI in another study (Chen 2012,[19] Romero Munoz 2019[21]).Diabetes Mellitus was associated with a non-significant decreased risk of ISCI in one study (Romero Munoz 2019[21]).Obesity was associated with an increased but non-significant risk of ISCI in one study (Romero Munoz 2019[21]). Another study reported a non-significant increased risk of ISCI with increasing body mass index (BMI) (Kim[20]).Clinical depression was associated with a non-significant increased risk of ISCI (Romero Munoz 2019[21]). Charleston Comorbidity Index (CCI) was also associated with a non-significant increased risk of ISCI (Kim[20]).Dyslipidemia has a non-significant association with decreased risk of ISCI in one study (Romero Munoz 2019[21]).Worse pulmonary function was reported to have increased risk of ISCI in one study that was non-significant (Zhang 2017 [23]).

Clinical Risk Factors for ISCI

Pre-operative neurological status was reported as a risk factor in two studies (Chen 2012[19], Zhang 2017[23]). In one study a better pre-operative neurological status was associated with a significantly decreased risk of ISCI (Chen 2012[19]). In a second study, the association was not significant, although it was associated with a decreased risk (Zhang 2017 [23]).Combined myelopathy was associated with a significant increased risk of ISCI in one study (Kim 2021[20]).

Blood loss was associated with an increased risk of ISCI in two studies (Fehlings 2018[11], Zhang 2017 [23]). The association was significant in one study, and non-significant in the other (Zhang 2017 [23], Fehlings 2018[11]). In a third study, blood loss had equivocal (OR=1) association with ISCI that was not significant (Kim 2021[20]).

Surgical Risk Factors for ISCI

A higher number of spinal levels was associated with increased risk of ISCI in two studies and with a decreased risk in one study. The increased risk was significant in one study and non-significant in the second study. The decreased risk was non-significant (Fehlings 2018,[11] Kim 2021[20], Chen 2012[19]).Increasing operation time was associated with increased risk of ISCI, however this was not significant (Kim 2021[20]). Lumbar level osteotomy was associated with a

For Peer Review

significant increased risk of ISCI. In the same study prevalence of three level osteotomy had a non-significant increased risk of ISCI (Fehlings 2018[11]).In another study, ponte-osteotomy was associated with a significant increased risk of ISCI (Buckland 2018[24]).Use of IONM was associated with a significant decreased risk of ISCI in one study (Kim 2021[20]).

Radiological Risk Factors ISCI

One study (Fehlings 2018)[11] on patients with scoliosis reported a greater odds of postoperative neurological deficit per 1 unit increase of coronal deformity angular ratio (DAR). One study (Buckland 2018)[24] in patients with adolescent scoliosis found a significant positive association between spinal curve magnitude and IONM alerts but did not report an effect estimate.

In addition to risk factors identified in the systematic review, GDG proposed seven characteristics of high-risk patients for ISCI. These included rigid thoracic curve with high deformity angular ratio, revision congenital Deformity with significant cord compression and myelopathy, extrinsic intradural or extradural Lesion with cord compression and myelopathy, intramedullary tumor, unstable fractures (bilateral facet dislocation and disc herniation, extension distraction injury with ankylosing spondylitis, ossification of posterior longitudinal ligament (OPLL) with severe cord compression and moderate to severe myelopathy. These risk factors were thoroughly discussed and voted upon by the GDG. Eventually, these risk factors were accepted as high risk after a unanimous vote according to the Delphi Process.

Quality (Strength) of Evidence

The overall quality (strength) of evidence for risk factors for ISCI based on multivariate analyses was low or very low for most factors across surgical conditions. (Table 10). Increased odds for ISCI varied by surgical indication/population (e.g., deformity). In patients undergoing surgery for spinal deformity, there was moderate evidence of increased odds for ISCI in patients with increasing age and increasing coronal DAR. There was moderate evidence that estimated blood loss and the number of spinal levels were not associated with increased odds of ISCI in the same population. There was moderate evidence that better preoperative AIS was associated with decreased odds of ISCI in a mixed population.

For Peer Review

Consensus Summary of Risk Factors for Intraoperative Spinal Cord Injury

Based on the knowledge synthesis summarized above and a consensus-based Delphi approach with the Guideline Development Group, a proposed list of risk factors for ISCIS was defined (Table 11).

DISCUSSION

The study of ISCI has been limited to date. Consequently, prior to this Focus issue, a paucity of evidence exists to guide clinicians in the decision-making surrounding patients who sustain an ISCI. This article has sought to identify the definition, frequency, risk factors, and management of ISCI through a scoping and systematic review of the existing literature.

Definition of ISCI:

To define ISCI a scoping review was conducted in which studies were reviewed and divided into two groups based on whether they focused on deformity or tumor surgery. Three studies by Lenke et al.,[12] Fehlings et al.[11] and Kato et al.[10] were centered around outcomes after adult deformity surgery. These outcomes were reported with respect to LEMS, where a major decline was defined as a loss of >5 points as this correlated to a deficit in 3 or more myotomes for 90% of the major decline group.[10] The aforementioned deficits were assessed at various time points postoperatively and therefore represent outcomes as a result of surgery rather than the natural history of the disease. The ASIA assessment is based on scoring of each key myotome from 0-5 on the MRC scale. The LEMS is the sum of all myotomes in the lower extremities bilaterally – this has been shown to correlate with ambulatory ability.[11]

Within tumor-based surgery, six studies provided definitions of ISCI. Each of these studies aimed to demonstrate the role of IONM in spinal cord tumor resection surgery. The definition of ISCI varied from a decrease in McCormick grade, MRC grade or neuromonitoring signal changes, with periods of assessment ranging from immediately postoperatively to outpatient follow up. The McCormick grade defines a patient's neurological impairment based on motor and sensory symptoms and functional status. This was used by Harel,[13] Korn[15] and Sala[17] to define ISCI resulting from spinal tumor resection surgery. Overall, the definition "a new or

For Peer Review

worsening neurological deficit attributable to spinal cord dysfunction during spine surgery that is diagnosed intra-operatively via neurophysiologic monitoring or immediately post-operatively based on clinical assessment" settled on for the purposes of this review is based on a combination of the studies included in this review and GDG recommendations. This definition encompasses both the role of IONM, and the clinical impact on the patient. The true impact of an ISCI is dependent on how the SCI affects the patient clinically; therefore, while the definition of ISCI relates to changes in neuromonitoring, further investigation is required to determine how IONM signals translate to clinical findings. Additionally, the review identified the most commonly used threshold for IONM indicative of ISCI as loss of 50% or more signal on IONM. The above-mentioned definition was reviewed by the GDG in the context of evidence collected from the scoping review. Changes were proposed and voted upon, in accordance with the Delphi Process. The use of wake-up test to detect ISCI was added to the definition as it was proposed that IONM is not readily available across the globe. This change was upheld after the voting process.

Based on the knowledge synthesis and a Delphi-based approach with the Guidelines Development Group, the following definition of Intraoperative Spinal Cord Injury was proposed:

"a new or worsening neurological deficit attributable to spinal cord dysfunction during spine surgery that is diagnosed intra-operatively via neurophysiologic monitoring or via wake-up test, or immediately post-operatively based on clinical assessment".

For Peer Review

Frequency of ISCI:

The frequency of ISCI varied across pathology and spinal level, as seen in Table 3. The data from this review suggested that the frequency of ISCI may be greater in tumor surgery with a range of 0-61% and persistent deficits up to 27%.[16] Of the 13 studies reporting on tumor surgery and IONM, one reported exclusively on extradural tumors (frequency of 1.97% with post-operative deficits), three on intradural extramedullary tumors (up to 16.5% with post-operative deficits), five on intramedullary tumors (up to 71.4% with ISCI) and the remainder on tumors in varied locations. This higher frequency of ISCI could be due in part to pre-existing deficits resulting from tumors within or abutting the cord.[25] [23,26]

Risk Factors of ISCI

The most commonly reported risk factors identified in this systematic review included older age, male sex, hypertension, pre-operative neurological status, blood loss, higher BMI, and number of spinal levels operated on. Older age, male sex, hypertension, combined myelopathy, blood loss, ponte-osteotomy, DAR, and curve magnitude were identified as factors with a statistically significant increased risk of ISCI. A better pre-operative neurological status, and use of IONM were identified as factors associated with a significant decreased risk of ISCI.

One of the most commonly cited risk factors was a decreased preoperative neurological status that can be considered evidence of preoperative spinal cord dysfunction.[21] This can be attributed to the fact that a damaged cord is considered more vulnerable (or potentially "with less reserve") to further insult.[19] Furthermore, it is possible that tumors result in intrinsic cord damage and that patients undergoing surgical resection may have more deficits preoperatively. These preoperative deficits may therefore increase the risk of ISCI as the spinal cord may be more susceptible to ischemia (on top of the obvious fact that the surgical approach often requires dissection through parts of the spinal cord that may be unaffected preoperatively).

The frequency of deficits differed by spinal level, with the lumbar spine representing the greatest frequency of ISCI. Some literature has suggested that the sparse blood supply in the thoracolumbar region may contribute to the relatively increased risk of ischemia and subsequent increased frequency of ISCI in this region.[26] Importantly, while the lumbar region has the highest reported frequency of ISCI, studies have identified a potential for injury to the cord during surgery at all spinal levels. Additionally, while injury at a lumbar spinal level may not, in the strictest definition, mean injury to the lumbar spinal cord but rather to the cauda equina (i.e., nerve root injury), for the purpose of this review, it was considered as ISCI as the etiology of the injury was still iatrogenic during spine surgery.

Several mechanisms have been suggested to describe the etiology of ISCI. Studies have proposed that injury is the result of direct mechanical trauma to the cord or from spinal cord ischemia.[27,28] Mechanical trauma can result from the placement of instrumentation or compression from surrounding structures such as the ligamentum flavum or the intervertebral disc. Vitale et al also discussed the implications of ischemia on ISCI and demonstrated that those with cardiopulmonary comorbidities were more likely to sustain ISCI as detected by IONM. This finding was not reported by the studies assessed in our scoping review – however, blood loss was

For Peer Review

described as a risk factor for ISCI in two studies and may potentially be a surrogate for cord perfusion.[28] Additionally, Zhang et al showed that pulmonary function had an increased risk of ISCI (although not significant).[23]

Older age was also reported as a risk factor for ISCI, presumably due to both the increased likelihood of postoperative complications and because of decreased neural tissue resilience with age.[19] Depression and Charleston Comorbidity Index was associated with an increased, but non-significant risk of ISCI. Hypertension showed a positive association with ISCI in one study but not another. As such, while this scoping review has identified several risk factors of ISCI, there was not enough evidence to establish the association between patients, surgical and disease characteristics and risk of ISCI. Further, it is not feasible to interpret age and co-morbidities in isolation as risk factors of ISCI, as these factors are related to each other. In future studies, authors should evaluate the association of frailty and risk of ISCI, as frailty index includes age and co-morbidities and can serve as a more comprehensive parameter.

Importantly, Kim et al reported a significant negative association between ISCI and the use of IONM (OR 0.14, p=0.003).[20] Specifically, this finding indicates that when IONM was used during surgery, the risk of ISCI was significantly decreased. Use of IONM is a modifiable factor unlike many other factors such as age and co-morbidities. IONM can detect ISCI in real time during surgery, which can notify the surgical team to take actions to minimize or even reverse the deficit when still possible.[23]

The curve magnitude and DAR ratios were identified as important risk factors for ISCI. More significant deformity can result in kinking or stretching of the cord and alter its hemodynamic supply.[23] These surgeries are naturally associated with a higher risk of ISCI due to direct manipulation of the neural elements, acute change in spinal canal alignment, the use of extensive spinal instrumentation and vascular insufficiency from stretch of the anterior spinal artery or over-shortening of the spinal column[1,2,13]Male sex was also shown to be a risk factor by three of the included studies. Chen et al attributed this to a potential protective effect of estrogen and progesterone in female patients.[19]Not all studies, however, reported a statistically significant association between sex and ISCI.

There are several limitations to this knowledge synthesis. Firstly, the majority of the studies available on this topic were rated as low quality. As there was previously no single definition,

For Peer Review

method, or criteria to define ISCI, there is variation in the reported frequency and risk factors of ISCI. Further, it is possible that not all included studied documented neurological score preoperatively using standard grading methods. There are also several strengths to our review. It is the first comprehensive and systematic knowledge synthesis that evaluates the frequency and risk factors of ISCI and provides a uniform definition. The methods used to conduct the knowledge synthesis were rigorous and abided by current standards. Additionally, the quality of the studies included was ascertained and reported. Specifically, the definition was thoroughly reviewed, debated, and voted upon by the GDG after the scoping review, and was formed after a unanimous vote, following the Delphi Process.

In summary, a comprehensive definition of ISCI was provided using the evidence gathered in this knowledge synthesis and input from GDG. This standardization of nomenclature for ISCI will enable future studies to better quantify the incidence of major neurological deficits, identify relevant risk factors and assess treatment protocols for ISCI management. Furthermore, by combining the results on risk factors with the frequency data, and recommendations by GDG, this review identified a subset of patients at "higher risk" for ISCI. These patients include older patients, those with high grade tumors causing compression, severe rigid deformity requiring multiple osteotomies, or structural pathologies causing myelopathy (e.g., OPLL) and those undergoing revision surgery.

While, in theory, the risk of ISCI can never be eliminated there can be strategies developed to mitigate and minimize it. This further raises the question of the role of IONM, management strategies in the event of loss on IONM, and a potential care pathway to mitigate the adverse event.

CONCLUSION

After a comprehensive scoping review, and expert opinion input, a consensus-based definition for ISCI was developed. It has been identified that most studied have reported that a decrease of 50% or more on IONM parameter can be considered indicative of ISCI. Additionally, several clinical, surgical, and radiological risk factors for ISCI have been identified. The results synthesized in this manuscript will supplement clinicians' knowledge of frequency and risk factors for ISCI in order to inform decision making regarding prevention and management

For Peer Review

strategies. A uniform, consensus-based definition of ISCI will also aid in optimizing future research on this topic. Additionally, this review further raises the question of the role of IONM, management strategies in the event of an IONM alert, and a potential care pathway to manage ISCI.

FIGURE LEGENDS

Figure 1: Search Strategy.

Figure 2: Literature search and study selection flow diagram for the systematic review on risk factors of ISCI.

For Peer Review

REFERENCES

1. Daniels AH, Hart RA, Hilibrand AS, et al. Iatrogenic Spinal Cord Injury Resulting From Cervical Spine Surgery. *Global Spine J*. 2017;7(1 Suppl):84S - 90S.

2. Ahn H, Fehlings MG. Prevention, identification, and treatment of perioperative spinal cord injury. *Neurosurg Focus*. 2008;25(5):E15.

3. Methods guide for effectiveness and Comparative Effectiveness Reviews. Accessed October 27, 2022. https://effectivehealthcare.ahrq.gov/products/collections/cer-methods-guide

4. Procedure manual. Accessed October 3, 2022. https://www.uspreventiveservicestaskforce.org/uspstf/about-uspstf/methods-and-processes/procedure-manual

5. Ghatol D, Widrich J. *Intraoperative Neurophysiological Monitoring*. StatPearls Publishing; 2022.

6. Hayden JA, van der Windt DA, Cartwright JL, Côté P, Bombardier C. Assessing bias in studies of prognostic factors. *Ann Intern Med*. 2013;158(4):280-286.

7. Iorio A, Spencer FA, Falavigna M, et al. Use of GRADE for assessment of evidence about prognosis: rating confidence in estimates of event rates in broad categories of patients. *BMJ*. 2015;350:h870.

8. Foroutan F, Guyatt G, Zuk V, et al. GRADE Guidelines 28: Use of GRADE for the assessment of evidence about prognostic factors: rating certainty in identification of groups of patients with different absolute risks. *J Clin Epidemiol*. 2020;121:62-70.

9. Dalkey N, Helmer O. An Experimental Application of the DELPHI Method to the Use of Experts. *Manage Sci*. 1963;9(3):458-467.

10. Kato S, Fehlings MG, Lewis SJ, et al. An Analysis of the Incidence and Outcomes of Major Versus Minor Neurological Decline After Complex Adult Spinal Deformity Surgery: A Subanalysis of Scoli-RISK-1 Study. *Spine* . 2018;43(13):905-912.

11. Fehlings MG, Kato S, Lenke LG, et al. Incidence and risk factors of postoperative neurologic decline after complex adult spinal deformity surgery: results of the Scoli-RISK-1 study. *Spine J*. 2018;18(10):1733-1740.

12. Lenke LG, Fehlings MG, Shaffrey CI, et al. Neurologic Outcomes of Complex Adult Spinal Deformity Surgery: Results of the Prospective, Multicenter Scoli-RISK-1 Study. *Spine* . 2016;41(3):204-212.

13. Harel R, Schleifer D, Appel S, Attia M, Cohen ZR, Knoller N. Spinal intradural extramedullary tumors: the value of intraoperative neurophysiologic monitoring on surgical outcome. *Neurosurg Rev*. 2017;40(4):613-619.

14. Kang H, Gwak HS, Shin SH, et al. Monitoring rate and predictability of intraoperative monitoring in patients with intradural extramedullary and epidural metastatic spinal tumors. *Spinal Cord*. 2017;55(10):906-910.

For Peer Review

15. Korn A, Halevi D, Lidar Z, Biron T, Ekstein P, Constantini S. Intraoperative neurophysiological monitoring during resection of intradural extramedullary spinal cord tumors: experience with 100 cases. *Acta Neurochir* . 2015;157(5):819-830.

16. Lakomkin N, Mistry AM, Zuckerman SL, et al. Utility of Intraoperative Monitoring in the Resection of Spinal Cord Tumors: An Analysis by Tumor Location and Anatomical Region. *Spine* . 2018;43(4):287-294.

17. Sala F, Palandri G, Basso E, et al. Motor evoked potential monitoring improves outcome after surgery for intramedullary spinal cord tumors: a historical control study. *Neurosurgery*. 2006;58(6):1129-1143; discussion 1129-1143.

18. Skinner SA, Nagib M, Bergman TA, Maxwell RE, Msangi G. The initial use of free-running electromyography to detect early motor tract injury during resection of intramedullary spinal cord lesions. *Neurosurgery*. 2005;56(2 Suppl):299-314; discussion 299-314.

19. Chen Q, Li F, Wu W. Risk factors of iatrogenic spinal cord injury in spinal surgery: a multicenter retrospective study. *Int J Neurosci*. 2012;122(10):606-610.

20. Kim JE, Kim JS, Yang S, et al. Neurophysiological monitoring during anterior cervical discectomy and fusion for ossification of the posterior longitudinal ligament. *Clin Neurophysiol Pract*. 2021;6:56-62.

21. Romero-Muñoz LM, Segura-Fragoso A, Talavera-Díaz F, Guimbard-Pérez J, Caba-Mora D, Barriga-Martín A. Neurological injury as a complication of spinal surgery: incidence, risk factors, and prognosis. *Spinal Cord*. 2020;58(3):318-323. doi:10.1038/s41393-019-0367-0

22. Iyer RR, Vitale MG, Fano AN, et al. Establishing consensus: determinants of high-risk and preventative strategies for neurological events in complex spinal deformity surgery. *Spine Deform*. 2022;10(4):733-744.

23. Zhang BB, Zhang T, Tao HR, et al. Neurological complications of thoracic posterior vertebral column resection for severe congenital spinal deformities. *Eur Spine J*. 2017;26(7):1871-1877.

24. Buckland AJ, Moon JY, Betz RR, et al. Ponte Osteotomies Increase the Risk of Neuromonitoring Alerts in Adolescent Idiopathic Scoliosis Correction Surgery. *Spine* . 2019;44(3):E175-E180.

25. Kumar N, Tan WLB, Wei W, Vellayappan BA. An overview of the tumors affecting the spine-inside to out. *Neurooncol Pract*. 2020;7(Suppl 1):i10-i17.

26. Cramer DE, Maher PC, Pettigrew DB, Kuntz C 4th. Major neurologic deficit immediately after adult spinal surgery: incidence and etiology over 10 years at a single training institution. *J Spinal Disord Tech*. 2009;22(8):565-570.

27. Bridwell KH, Lenke LG, Baldus C, Blanke K. Major intraoperative neurologic deficits in pediatric and adult spinal deformity patients. Incidence and etiology at one institution. *Spine* . 1998;23(3):324-331.

28. Vitale MG, Moore DW, Matsumoto H, et al. Risk factors for spinal cord injury during surgery for spinal deformity. *J Bone Joint Surg Am*. 2010;92(1):64-71.

For Peer Review

#1	"Spinal Injuries"[Mesh] OR "Spinal Diseases"[Mesh] OR "Spinal Cord Compression"[Mesh] OR "Spinal Cord Injuries"[Mesh] OR "Spinal Cord Neoplasms"[Mesh] OR "Spinal Cord Ischemia"[Mesh] OR "Osteoarthritis, Spine"[Mesh] OR "Radiculopathy"[Mesh] OR "myelopathy" OR "Cauda Equina Syndrome"[Mesh] OR "cauda equina" OR "conus medullaris"
#2	"Iatrogenic Disease"[Mesh] OR "Intraoperative Complications"[Mesh] OR "Postoperative Complications"[Mesh]
#3	"Aorta, Abdominal"[Mesh] OR "Vascular Surgical Procedures"[Mesh] OR "Stroke"[Mesh] OR "aortic aneurysm"
#4	"Aorta, Abdominal"[Mesh] OR "Vascular Surgical Procedures"[Mesh] OR "Stroke"[Mesh] OR "aortic aneurysm" OR hematoma OR abscess OR palsy
#5	(#1 AND #2) NOT #4: LIMIT ABSTRACT, HUMANS, ENGLISH

Figure 1: Search Strategy.

697x258mm (38 x 38 DPI)

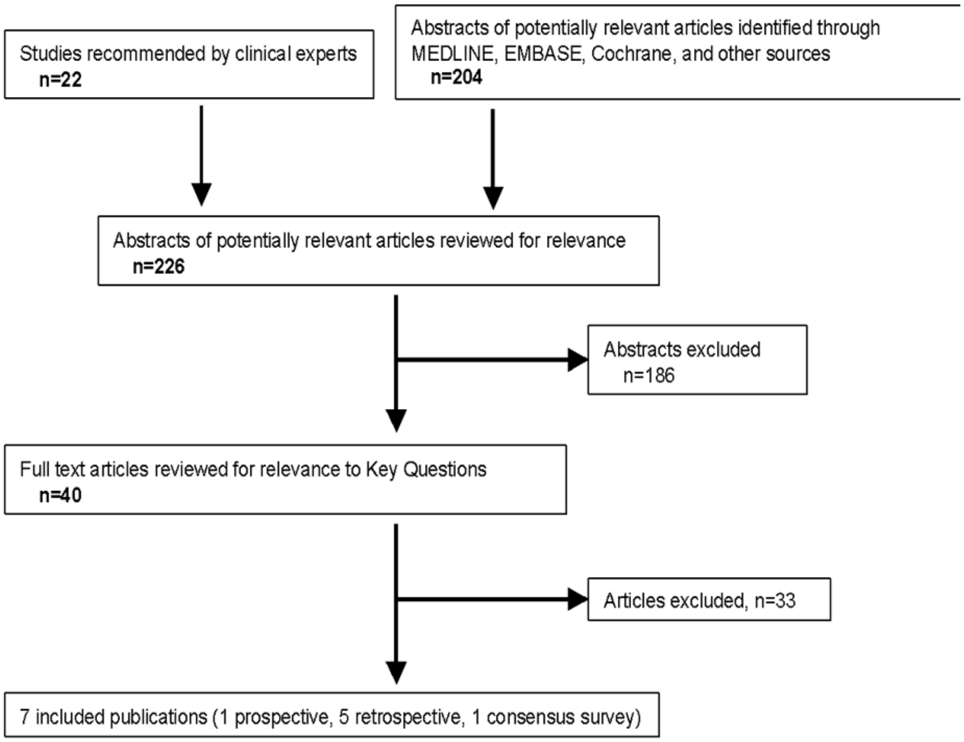

Figure 2: Literature search and study selection flow diagram for the systematic review on risk factors of ISCI.

639x483mm (38 x 38 DPI)

Table 1: Review Key Question on Risk factors for ISCI: Inclusion and exclusion criteria - population, prognostic factors, outcomes, studies.

	Inclusion	Exclusion
**Patients**	• Adolescents (≥11 years to <18 years old) or adults (≥18 years) undergoing any type of spine surgery for any indication or spine-related pathology (including trauma-related pathology, conus injuries, cauda equina injuries)	• Patients <11 years old<br>• Patients undergoing surgery for pathologies not involving the spine (e.g., vascular surgeries)<br>• Patients with new post-operative compression (e.g., hematoma, abscess)<br>• Patients with root-level deficits<br>• Patients with neurological deficits due to cranial pathology (e.g., stroke)
**Prognostic factors of interest**	**Primary Factors of interest**:<br>• **Clinical/pathology factors** (e.g., tumors [intra or extra-dural], scoliosis or other spinal deformity, myelopathy, trauma)<br>• **Surgical factors** (e.g., surgical procedure, surgical approach, number of levels)<br>**Potential confounding factors:**<br>• **Demographic factors** (age, sex, BMI, smoking)	

For Peer Review

**Outcome**	Documented intra-operative spinal cord injury defined as: new or worsening neurological deficit attributable to spinal cord dysfunction that occurs as the result of spine surgery either intra-operatively (diagnosed via intra-operative monitoring) or in the immediate post-operative period (based on clinical assessment); includes cauda-equina syndrome and conus compression in addition to cord compression.	• Root-level injuries, nerve palsies, peripheral nerve injury<br>• Hematoma, abscess<br>• Ischemia following aorta or vascular surgery<br>• Anesthesia effects<br>• Brain/intracranial effects, pathologies (e.g., stroke)
**Studies**	Studies with the highest methodological quality were focused on.<br>• Prospective studies: if no prospective studies were available, retrospective studies were considered.<br>• Only studies which controlled for confounding (e.g., patient, or clinical factors) were included for formal risk factor evaluation.<br>• Case series and narrative reviews were considered in the absence of formal controlled studies of risk factors.	• Studies with <15 patients<br>• For formal risk factor evaluation, studies which do not control for potential confounding.<br>• Case reports, case series, conference proceedings, abstracts, letters, white papers, cross-sectional studies<br>• Animal or cadaver studies

For Peer Review

Table 2: Criteria for grading the quality of individual studies.

**Rating**	**Description and Criteria**
**Good**	• Low risk of bias, most criteria for quality are met, and results are generally considered valid<br>• Valid methods for selection, inclusion, and treatment allocation; report similar baseline characteristics in different treatment groups; clearly describe attrition and have low attrition; appropriate means for preventing bias and use of appropriate analytic methods
**Fair**	• Some study flaws: may not meet all criteria for good quality, but no flaw is likely to cause major bias that would invalidate results; the study may be missing some information making it difficult to assess limitations and potential problems. This is a broad category; results from studies may or may not be valid.
**Poor**	• Significant flaws that imply biases of various kinds that may invalidate results; most criteria for a good quality study are not met and/or "fatal flaws" in design, analysis, or reporting are present; large amounts of missing information; discrepancies in reporting; or serious problems with intervention delivery

For Peer Review

Table 3: Contextual Question: Criteria/thresholds for intra-operative SCI and frequency

Deformity				
Author, Year	Definition	Indication of Surgery	Alert Definition/ Threshold	Timepoint
Kato 2018 (SCOLI)	Loss of LEMS at discharge in comparison to preoperative (baseline) status.	Complex adult spinal deformity	Major decline: LEMS loss of 5 points or more<br>Minor decline: LEMS loss of less than 5 points	LEMS compared to preoperative baseline
Fehlings 2018 (SCOLI)	Postoperative deterioration in ASIA LEMS compared with preoperative status	Complex adult spinal deformity	Postoperative deterioration in ASIA LEMS compared with preoperative status, threshold NR	ASIA LEMS at discharge
Lenke 2016 (SCOLI)	Decrease in ASIA LEMS postoperative compared to preoperative	Complex adult spinal deformity	Decrease in LEMS postop compared to preoperative, with normal LEMS= 50, abnormal LEMS <50.	ASIA LEMS at 6 weeks
Tumors				
Harel 2017	Decrease in McCormick grade	Intradural extramedullary tumors	Mean McCormick grade. Patients graded as either stable or deteriorated compared to preoperative state.	Immediate postoperative, further follow-up not defined.

For Peer Review

Kang 2017	Motor power as graded by the Medical Research Council System	Intradural extramedullary and epidural metastatic spinal tumors	Postoperative drop in patient's motor power by ≥1 grade	Acute period, 2 days postoperative, and before rehabilitation treatment
Korn 2014	Decrease in modified McCormick grade	Extramedullary spinal cord tumors	Mean McCormick grade. Patients graded as either stable or deteriorated compared to preoperative state.	3 hours postoperative, 3 months, 6 months follow-up
Lakomkin 2017	IONM signal change	Spinal cord tumors	True-positive defined when IONM signal change correlated either with a new, permanent postoperative motor and/or sensory deficit that remained present at a 6months follow-up	6 months
Sala 2006	Decrease in McCormick grade	Intramedullary spinal cord tumor	Patients given a value of 0 if they remained stable after surgery, and -1, -2, or -3 if they deteriorated after surgery by 1, 2, or 3 grade(s) on the McCormick scale, and +1, +2, +3 if they improved	Discharge and 3+ month follow-up
Skinner 2005	Intra-operative warning to surgeon	Intramedullary spinal cord lesions	True-positive case defined as a new or worsened postoperative motor deficit that was predicted by means	Postoperative, long-term follow-up varied by patient

For Peer Review

			of an intra-operative warning to the surgeon	(range: 1-28 months)

For Peer Review

Table 4: Frequency of deficits spanning pathology type and level of surgery.

Condition	Number of studies (Range of sample size)	New deficit % (n/N)	Resolved Deficit % (n/N)	Persistent Deficit % (n/N)
**Deformity**	13 studies (28 to 1121)	0% (0/144, 0/452, and 0/97) to 17.8% (5/28)	NR to 92% (200/217)	NR to 8% (17/217)
**Tumor**	13 studies (13 to 1017)	0% (0/19 and 0/68) to 61% (8/13)	34% (17/50) to 67% (681/1017)	7.4% (15/203) to 26.9% (14/52)
**Level**	**Number of studies (Range of sample size)**	**New deficit % (n/N)**	**Resolved Deficit % (n/N)**	**Persistent Deficit % (n/N)**
**Cervical**	11 studies (52 to 1445)	0.09 (1/1039) to 5.7% (10/175)	1.8% (1/57) to 100% (246/246)	NR
**Thoracolumbar**	2 studies (173 to 295)	0.7% (2/295) to 5.8% (10/173)	NR	NR
**Thoracic**	3 studies (r 44 to 871)	0.6% (5/871) to 13.4% (11/82)	NR to 100% (44/44)	NR
**Lumbar**	3 study (35 to 113)	0 (0/113) to 28.5% (10/35)	NR	NR
**Mixed**	16 studies (5 to 2069)	0% (0/2069) to 9.4 (6/64)	2.2% (9/408) to 6.3% (4/64)	0.5% (1/176) to 4.9% (20/408)

For Peer Review

Table 5: Data abstraction for included studies looking at risk factors for intraoperative neurological deficits.

Author<br>Design	Population	Exclusion Criteria	Surgery	Intraoperative SCI definition and Incidence	Risk Factors Assessed*<br>Effect Estimate (95% CI)†	Funding<br>COI
Deformity						
Fehlings 2018<br><br>Prospective cohort<br><br>Study quality: Good	Surgical Indication: ASD with an apex of the major deformity in the cervicothoracic or the thoracolumbar region between C7 and L2 inclusive undergoing complex ASD surgery‡<br>Eligible: N = 272<br>Analyzed: N = 265<br>Mean age: 56.8 ± 15.4 years<br>Males: 32%	• Substance dependency<br>• Psychosocial disturbance<br>• Active malignancy<br>• Active bacterial infection<br>• Recent history of significant spinal trauma or malignancy<br>• Complete long-term paraplegia<br>• Pregnancy<br>• Prisoners<br>• Institutionalize	• Three-column osteotomy between C7 and L5 inclusive (76%)<br>• Corrective osteotomies for revision of spinal deformity (61%) or for congenital spinal deformity (5%)<br>• Corrective surgery for curvature with major Cobb angle of ≥80˚ in the coronal or sagittal plane (29%)<br>• Reconstruction for deformity-related	Decline in ASIA lower extremity motor score at discharge compared to preoperative status.<br><br>Incidence: 23% (61/265)	Multivariable analysis<br>• Age (per 10 years): OR = 1.53 (95% CI: 1.13 to 2.06)<br>• Coronal deformity angular ratio (per 1 unit): OR = 1.10 (95% CI: 1.01 to 1.19)<br>• Number of spinal levels involved (per 1 level): OR = 1.08 (95% CI: 0.99 to 1.17)<br>• Lumbar-level osteotomy (yes vs. no): OR = 3.30 (95% CI: 1.18 to 9.17)<br>• Prevalence of 3CO (yes vs. no): OR = 2.16 (95% CI: 0.77 to 6.08)<br>• Estimated blood loss (per 500 cc): OR = 1.06 (95% CI: 0.97 to 1.15)<br><br>Univariate analysis<br>• Previous history spine surgery (yes vs. no)<br>• Preoperative neurological deficits (yes vs. no)<br>• Sagittal deformity angular ratio (per 1 unit)<br>• Surgical approach (anterior-posterior vs.	Funding: Scoliosis Research Society and AOSpine International, Norton Healthcare<br><br>COI: None

For Peer Review

		d individuals	myelopathy (5%)<br>• Deformity reconstruction with concomitant spinal cord decompression for OLF or OPLL (2%)<br>• Osteotomy level:<br>Lumbar: 73%<br>Thoracic: 43%<br>• Surgical approach:<br>Posterior only: 77%<br>Anterior-posterior: 23%		posterior only)	
Zhang 2017<br><br>Retrospective cohort<br><br>Study quality: Fair	Surgical Indication:<br>Congenital scoliosis (19.3%), kyphoscoliosis (74.2%), kyphosis (6.5%)<br>Eligible: N = NR<br>Analyzed: N = 62<br>Mean age (range):	NR	• Thoracic posterior vertebral column resection	Decrease in ASIA grade at discharge. Not explicitly defined.<br><br>Incidence: 16.1% (10/62)	Multivariable analysis<br>• Age (≥18 vs. <18 years): OR = 8.27 (95% CI: 1.17 to 58.71)<br>• Pulmonary function (normal vs. abnormal): OR = 2.10 (95% CI: 0.99 to 4.48)<br>• Pre-operative neurological status (normal vs. abnormal): OR = NR, p>0.05<br>• Blood loss (>50% vs. <50%): OR = 3.05	Funding: Science and technology innovation project in Shaanxi Province of China, Natural Science Foundation of China, Natural

For Peer Review

	16.3 (6 to 46) years<br>Males: 45.2%				(95% CI: 1.16 to 8.05)<br><u>Univariate analysis</u><br>• Cobb (main curve) (≥90 vs. <90)<br>• Operative time (≥480 vs. <480 minutes)<br>• BMI (normal vs. abnormal)<br>• Malformation type (kyphoscoliosis vs. scoliosis + kyphosis)<br>• Number of vertebrae fused (≥10 vs. ≤10)<br>• Number of vertebrae resected (≥2 vs. 1)<br>• Use of titanium mesh/cage (yes vs. no)<br>• Intraspinal deformity (yes vs. no)	Science Basic Research Plan in Shaanxi Province in China, and the Youth Development Project of the Army Medical Technology<br><br>COI: None
Mixed surgical indications						
Chen 2012<br><br>Retrospective cohort (matched pairs)[§]<br><br>Study quality: Good	Surgical Indication:<br>Spinal degeneration (35%), spinal tumor (23%), spinal trauma (22%), spinal deformity (16%), and spinal inflammation (4%)<br>Eligible: NR<br>Analyzed: N = 316	• Major and current psychiatric illnesses or cognitive deficits<br>• Neurological function of spinal cord could not be measured correctly	• Decompression<br>• Internal fixation<br>• Bone graft<br>• Reduction<br><br>(No other information provided)	Iatrogenic SCI defined as a decrease in postoperative ASIA grade. No further details given.<br><br>Incidence: 25% (79/316)[**]	<u>Multivariable analysis</u><br>• Age (continuous[§§]): OR = 1.08 (95% CI: 1.03 to 1.13)<br>• Sex (male vs. female): OR = 5.22 (95% CI: 1.86 to 14.62)<br>• Hypertension (yes vs. no): OR = 15.18 (95% CI: 4.50 to 51.17)<br>• Preoperative spinal cord function (AIS A-D; better vs. worse): OR = 0.35 (95% CI: 0.18 to 0.66)<br>• Involved segments (more vs. less): OR =	Funding: Ministry of Science and Technology of the People's Republic of China<br><br>COI: None

For Peer Review

	Mean age: 33.33 ± 7.69 years<br>Males: 70%	• Significant traumatic brain injuries<br>• Major medical diseases<br>• Major neurological deficits or diseases<br>• Pregnancy<br>• Life-threatening injuries or other concurrent injuries which prevent spinal surgery			3.28 (95% CI: 1.55 to 6.92)<br>Univariate analysis<br>• Diabetes mellitus<br>• Extent of compression to spinal cord on MRI (normal, decompression without ischemia and ischemia)	
Romero-Munoz 2019<br><br>Retrospective cohort<br><br>Study	Cases were of intra-op SCI from elective surgery, control population appear to be SCI from other presentation	• SCI following primary or metastatic cancer surgery<br>• SCI secondary to diagnostic procedures,	Cervical<br>• Discectomy and arthrodesis<br>• Corpectomy and arthrodesis<br><br>Lumbar	Decrease in ASIA grade at discharge. Not explicitly defined.<br><br>Incidence: 9%	Multivariable analysis (all adjusted for age)<br>• Age (continuous[§§], median): OR = 1.004 (95% CI: 0.98 to 1.03)<br>• Diabetes mellitus (yes vs. no): OR = 0.70 (95% CI: 0.20 to 2.38)<br>• Obesity (yes vs. no): OR = 0.52 (95% CI: 0.16 to 1.69)	Funding: None received<br><br>COI: None

For Peer Review

quality: Fair	Surgical Indication: Spinal degeneration (i.e., stenosis, disc herniation, spondylolisthesis w/w/o spinal stenosis), primarily presenting with pain and radiculopathy without deficit (75%); spinal deformity (i.e., scoliosis or kyphosis) (17.5%); spine fracture (3.5%); other[‡‡] (4%)<br>Location of SCI: Cervical (30%), Thoracic (43%), and Lumbar (27%)<br>Eligible: N = 1282	epidural puncture or procedures performed during childbirth	• Laminectomy and discectomy<br>Laminectomy and arthrodesis	(114/1282)[***]	• Hypertension (yes vs. no): OR = 1.47 (95% CI: 0.56 to 3.86)<br>• Dyslipidemia (yes vs. no): OR = 0.48 (95% CI: 0.16 to 1.47)[††]<br>• Depression (yes vs. no): OR = 2.69 (95% CI: 0.95 to 7.59)<br><br><u>Univariate analysis</u><br>• Sex (female vs. male)<br>• Spinal Cord Independence Measure at baseline (continuous, median)<br>• AIS scale at baseline (absolute frequency, %)	

For Peer Review

	Analyzed: N = 1282<br>Mean age (IQR): 58 (45 to 69) years<br>Males: 54.4%					
Degenerative spine disease						
Kim 2021<br><br>Retrospective cohort<br><br>Study quality: Poor	Surgical Indication: OPLL<br>Eligible: N = 210<br>Analyzed: N = 196<br>Mean age: NR<br>Males: 67.3	• Other indications for ACDF than OPLL (infection, fracture, tumor and/or inflammatory and congenital musculoskeletal disorders<br>• Patients undergoing other concurrent surgery (e.g., posterior cervical fusion, occipito-	• Anterior cervical discectomy with fusion with/without corpectomy	Postoperative neurological complications defined as "any new limb motor or sensory neurological deficits observed immediately post-operation". No further details given.<br><br>Incidence: 7.1% (14/196)	<u>Multivariable analysis</u><br>• Sex (male vs. female): OR = 1.378 (95% CI: 0.33 to 5.79)<br>• Age (continuous§§): OR = 0.97 (95% CI: 0.89 to 1.05)<br>• BMI (continuous§§): OR = 1.11 (95% CI: 0.96 to 1.29)<br>• Compressive myelopathy prior to surgery (yes vs. no): OR = 8.24 (95% CI: 1.57 to 43.38)<br>• CCI score (categorical, 0, 1, 2, or ≥3; lower vs. higher): OR = 1.02 (95% CI: 0.49 to 2.14)<br>• Operation type (emergency vs. elective): OR = 0.00 (95% CI: NR), p=0.999<br>• Operative time (continuous§§): OR = 1.004 (95% CI: 0.99 to 1.01)<br>• Number of levels fused (1 to 2 vs. ≥3): OR = 1.36 (95% CI: 0.59 to 3.11)<br>• Blood loss (continuous§§): OR = 1.00 (95% CI: 0.99 to 1.002)	Funding: None<br><br>COI: None

For Peer Review

		cervical fusion, atlanto-axial fusion, cranial surgery)<br>• Inadequate medical records to confirm postoperative neurological state			• Use of intraoperative neuromonitoring (yes vs. no): OR = 0.14 (95% CI: 0.04 to 0.52)<br><br>Univariate analysis<br>• Race (Asian vs. other)	

3CO = 3 column osteotomy; AIS = ASIA Impairment Scale; ASD = adult spinal deformity; ASIA = American Spinal Injury Association; CCI = Charlson Comorbidity Index; COI = conflict of interest; CI = confidence interval; ICD-9 = International Classification of Diseases, Ninth Revision; IQR = interquartile range; NR = Not reported; OLF = ossification of the ligamentum flavum; OPLL = ossification of the posterior longitudinal ligament; OR = Odds Ratio; ROB = Risk of bias; SCI = Spinal cord injury.

* If no definition of variable is listed, the article did not provide one.

† Risk factors represent all variables in a given study's univariate and multivariable analyses. Only those with effect sizes were included in multivariable analyses; all others were explicitly stated to have not been used beyond univariate analyses due to non-significance, but were otherwise assessed for an association using univariate statistical methods.

‡ Patients were selected based on the procedure performed: had to be one of the surgeries in the surgery column.

For Peer Review

§ Matched-pairs. Patient with SCI (n=79) were matched with patients without SCI (n=237) in a 1:3 ratio on the following factors: primary disease, hospital, and similar procedure.

** All cases, as controls were included based on lack of SCI.

†† Text in results says dyslipidemia is only factor to show significance (OR=0.34, 95% CI: 0.12 to 0.96, p=0.04), however table 9 summarizing the results of all factors in multivariable regression lists that dyslipidemia is not significant (OR=0.48, 95% CI: 0.16 to 1.47, p=0.197).

‡‡ Arnold Chiari, vertebroplasty, cervical epidural electrode, coccygodynia.

§§ Authors do not report details, assumed continuous.

*** 114 cases of SCI following elective surgery.

For Peer Review

Table 6: Data abstraction for included studies looking at risk factors for intraoperative monitoring warnings.

Author Design	Population	Exclusion Criteria	Surgery	Intraoperative SCI definition	Risk Factors Assessed* Effect Estimate (95% CI)†	Funding COI
Deformity						
Buckland 2018<br><br>Retrospective cohort<br><br>Study quality: Poor	Surgical Indication: Adolescent idiopathic scoliosis<br>Eligible: N = 2210<br>Analyzed: N = 2210<br>Mean age: 14.7 ± 2.1 years<br>Males: 19.4%	NR	• Ponte osteotomy<br>• No Ponte osteotomy	Perioperative nerve root or SCI as identified by surgeon. Intraoperative neuromonitoring alerts as outcome. No further detail given.<br><br>Intraoperative neuromonitoring warnings defined as:<br>SSEP: Decrease ≥50% amplitude<br><br>TcMEP: Decrease ≥50% amplitude	Multivariable analysis<br>• Ponte osteotomy (yes vs. no): OR = NR, p<0.001<br>• Curve magnitude (continuous‡): OR = NR, p<0.001<br><br>Univariate analysis<br>Unclear	Funding: DePuy Synthes Spine<br><br>COI: Board membership, consultancy, royalties, grants, stocks, employment, payment for lecture, patients. No further details given.

For Peer Review

				Incidence: 0.3% (7/2210)		

3CO = 3 column osteotomy; AIS = ASIA Impairment Scale; ASIA = American Spinal Injury Association; CCI = Charlson Comorbidity Index; COI = conflict of interest; CI = confidence interval; IQR = interquartile range; NR = Not reported; OR = Odds Ratio; ROB = Risk of bias; SCI = Spinal cord injury; SSEP = Somatosensory evoked potentials; TcMEP = Transcranial motor evoked potentials.

* If no definition of variable is listed, the article did not provide one.

† Risk factors represent all variables in a given study's univariate and multivariable analyses. Only those with effect sizes were included in multivariable analyses; all others were explicitly stated to have not been used beyond univariate analyses due to non-significance, but were otherwise assessed for an association using univariate statistical methods.

‡ Authors do not report details, assumed continuous.

For Peer Review

Table 7: Demographic, clinical, surgical, and radiographic factors potentially associated with intra-operative SCI in studies that conducted multivariable analyses. *

	Risk factors for Neurological Deficits					
	**Deformity**		**Mixed surgical indication**		**Degeneration**	**Deformity**
	Fehlings 2018	Zhang 2017	Chen 2012	Romero-Muñoz 2019	Kim 2021	Buckland 2018
Intraoperative SCI definition	ASIA	ASIA	ASIA	ASIA	Unclear†	IONM‡
Surgical indication	Scoliosis	Congenital scoliosis, kyphoscoliosis, kyphosis	Spinal degeneration, tumor, trauma, deformity, inflammation	Cervical, lumbar spine injuries	OPLL	Adolescent idiopathic scoliosis
ROB:	Low	Moderate	Low	Moderate	High	High
Risk Factor						

For Peer Review

Patient characteristics	Age	Yes	Yes	Yes	No	No	
	Sex			Yes		No	
	Hypertension			Yes	No		
	Diabetes				No		
	BMI/Obesity				No	No	
	Depression				No		
	CCI score					No	
	Dyslipidemia				No		
	Pulmonary function		Yes[§]				
Clinical	Preoperative AIS/neurological status		No	Yes			

	Combined myelopathy					Yes	
	Blood loss	No	Yes			No	
	No. of spinal levels/involved segments	No		Yes		No	
	Operation type					No	
	Operation time					No	
	Use of intra-operative monitoring					Yes	
	Lumbar-level osteotomy	Yes					
Surgical	Ponte-osteotomy						Yes

	Prevalence of 3CO	No					
Radiographic	Coronal DAR	Yes					
	Curve magnitude						Yes

3CO = 3 column osteotomy; ASIA = American Spinal Cord Injury Association; AIS = ASIA Impairment Scale; BMI = Body mass index; CCI = Charlson Comorbidity Index; DAR = deformity angular ratio; ICD-9 = International Classification of Diseases, Ninth Revision; IONM = Intra-operative neuromonitoring; OPLL = ossification of posterior longitudinal ligament; SCI = Spinal cord injury, ROB = risk of bias.

* “Yes” indicates that a given factor was associated with the outcome in multivariate analysis; “no” indicates that a given factor was not associated in multivariate analysis.

† Kim 2021 defined new neurological deficits as “any new limb motor or sensory neurological deficits observed immediately post-operation”. Every included patient was checked for their neurological status preoperatively, immediately after awaking from anesthesia, 1 day after operation, discharge period and follow up periods at outpatient clinics. No further details given.

‡ Authors report that a neuromonitoring alert was defined as a reduction in amplitude of 50% or more in SSEPs and/or tcMEPs. Increases in response latency were not included based on prior literature suggesting that it was not an independent sign of neurological injury in spine surgery.

For Peer Review

§ Marginal association. OR=2.10 (95% CI 0.99 to 4.48), $p=0.054$

For Peer Review

Table 8: All demographic, clinical, surgical, and radiographic factors explored/evaluated as prognostic factors for intraoperative SCI in univariate analyses[*]

	Risk factors for Neurological Deficits					Risk factors for IONM[‡]	Expert Consensus
	Deformity		Mixed surgical indication		Degeneration	Deformity	
	Fehlings 2018	Zhang 2017	Chen 2012	Romero-Muñoz 2019	Kim 2021	Buckland 2018	Iyer 2022[§]
Intraop SCI definition	ASIA	ASIA	ASIA	ASIA	Unclear[†]	IONM[‡]	IONM
Surgical indication	Scoliosis	Congenital scoliosis, kyphoscoliosis, kyphosis	Spinal degeneration, tumor, trauma, deformity, inflammation	Cervical, lumbar spine injuries	OPLL	Adolescent idiopathic scoliosis	Complex spinal deformity
ROB:	Low	Moderate	Low	Moderate	High	High	NA
Risk Factor							
Age	M	M	M	M	M		√
Sex			M	U	M		
Race					U		
Hypertension			M	M			
Diabetes			U	M			
BMI/Obesity		U		M	M		√

For Peer Review

	Depression				M			
	Previous surgery	U						
	CCI score					M		
	SCIM				U			
	Dyslipidemia				M			
	Intraspinal deformity		U					√
	Malformation type		U					
	Non-idiopathic etiology							√
	Congenital scoliosis							√
	Congenital kyphosis							√
	Syndromic etiology							√
	Neurological comorbidity							√
	Current tethered cord							√
	Split cord malformation							√
	Presence of syrinx ≥4mm							√

	Preoperative myelopathy							√
	Skeletal dysplasia							√
	Cardiopulmonary comorbidity							√
	Neuromuscular etiology							√
	Chronic anemia							√
	Presence of syrinx of any size							√
Clinical	Preoperative AIS/neurological status	U	M	M	U			
	Combined myelopathy					M		
	Blood loss	M	M			M		
	Pulmonary function		U					
	No. of spinal levels/involved segments	M		M		M		
	No. of levels/vertebrae fused		U					

	No. of vertebrae resected		U					
	Operation type	U				M		
	Operation time		U			M		
	Use of intraoperative monitoring					M		√
	Use of MRI			U				
	Use of titanium mesh/cage		U					
	Lumbar-level osteotomy	M						
	Ponte-osteotomy						M	
	Prevalence of 3CO	M						
	Revision surgery							√
	Prior ASF with vessel ligation							√
	Prior intradural surgery							√
	Absence of baseline IONM data, or poor-quality data							√
	Revision surgery							√

	Prior ASF with vessel ligation							√
	Prior intradural surgery							√
	Absence of baseline IONM data, or poor-quality data							√
	Hematocrit <28							√
	Higher ASA class							√
	Lack of preoperative HGT in an appropriate patient							√
	Prior fusion							√
	Intraoperative adjustment/movement of patient							√
Radiographic	Coronal DAR	M						√
	Sagittal DAR	U						√
	Cobb curve		U					
	Curve magnitude						M	√
	Total DAR							√

	Scoliosis with hyperkyphosis							√
	Bayoneted spine							√
	High rate of deformity progression							√
	Type 2 spinal cord shape							√
	Type 3 spinal cord shape							√
	Structural curve							√

3CO = 3 column osteotomy; ASIA = American Spinal Cord Injury Association; AIS = ASIA Impairment Scale; BMI = Body mass index; CCI = Charlson Comorbidity Index; DAR = deformity angular ratio; ICD-9 = International Classification of Diseases, Ninth Revision; OPLL = ossification of posterior longitudinal ligament; SCI = Spinal cord injury; SCIM = Spinal Cord Independence Measure.

* U represents factors explored only in univariate analyses; M represents factors explored at the multivariable level. Checkmarks indicate factors considered amongst a consensus survey.

† Kim 2021 defines new neurological deficits as "any new limb motor or sensory neurological deficits observed immediately post-operation". No further details given.

‡ Authors report that a neuromonitoring alert was defined as a reduction in amplitude of 50% or more in SSEPs and/or tcMEPs, but that increases in response latency were not included based on prior literature suggesting that it was not an independent sign of neurological injury in spine surgery.

§ Article included for context. Reports survey consensus amongst 15 experts and is not a patient-based multivariable assessment of factors associated with SCI. Checkmarks indicate all risk factors considered.

Table 9: Summary of Effect Estimates for Risk Factors for Intra-operative SCI in Studies Using Multivariable Analysis.

Prognostic Factor	Number of Studies (Number of patients)<br><br>Factor details: OR (95% CI)
**Demographic**	
Age	**Deformity**<br><br>Fehlings, 2018 (N=272)<br><br>Age (per 10 years): OR=1.53 (95% CI 1.13 to 2.06), p=0.05<br><br>Mean age: 56.8 ± 15.4 years<br><br>Zhang, 2017 (N=62)<br><br>Age (≥18 vs. <18 years): OR=8.27 (95% CI 1.17 to 58.71), p=0.035<br><br>Mean age: 16.3 ± 6.4 years<br><br>**Mixed**<br><br>Chen, 2012 (N=316)<br><br>Age (continuous*): OR=1.08 (95% CI 1.03 to 1.13), p<0.001<br><br>Mean age: 43.15 ± 6.47 years

For Peer Review

	Romero-Muñoz, 2019 (N=1282)<br><br>Age (continuous*): OR=1.004 (95% CI 0.98 to 1.03), p=0.759<br><br>Median age (IQR): 58 (45 to 69) years<br><br>**Degeneration**<br><br>Kim, 2021 (N=210)<br><br>Age (continuous*): OR=0.97 (95% CI 0.89 to 1.05), p=0.446<br><br>Mean age: 57 ± 12.2 vs. 58 ±12 years§
Sex	**Mixed**<br><br>Chen, 2012 (N=316)<br><br>Sex (male vs. female): OR= 5.22 (95% CI 1.86 to 14.62), p=0.002<br><br>**Degeneration**<br><br>Kim, 2021 (N=196)<br><br>Sex (male vs. female): OR=1.378 (95% CI 0.33 to 5.79), p=0.661
Hypertension	**Mixed**

For Peer Review

	Chen, 2012 (N=316)<br><br>Hypertension (yes vs. no): OR=15.18 (95% CI 4.50 to 51.17), p<0.001<br><br>Romero-Muñoz, 2019 (N=1282)<br><br>Hypertension (yes vs. no): OR=1.47 (95% CI 0.56 to 3.86), p=0.436
Diabetes	**Mixed**<br><br>Romero-Muñoz, 2019 (N=1282)<br><br>Diabetes mellitus (yes vs. no): OR=0.70 (95% CI 0.20 to 2.38), p=0.562
BMI/Obesity	**Mixed**<br><br>Romero-Muñoz, 2019 (N=1282)<br><br>Obesity (yes vs. no): OR=0.52 (95% CI 0.16 to 1.69), p=0.276<br><br>**Degeneration**<br><br>Kim, 2021 (n=196)<br><br>BMI (continuous*): OR=1.11 (95% CI 0.96 to 1.29), p=0.154
Depression	**Mixed**<br><br>Romero-Muñoz, (N=1282)<br><br>Depression (yes vs. no): OR=2.69 (95% CI 0.95 to 7.59), p=0.061

For Peer Review

CCI score	**Degeneration**<br><br>Kim, 2021 (n=196)<br><br>CCI score (categorical, 0, 1, 2, or ≥3; lower vs. higher): OR=1.02 (95% CI 0.49 to 2.14), p=0.953
Dyslipidemia	**Mixed**<br><br>Romero-Muñoz, (N=1282)<br><br>Dyslipidemia (yes vs. no): OR=0.48 (95% CI 0.16 to 1.47), p=0.197
Pulmonary Function	**Deformity**<br><br>Zhang, 2017 (N=62)<br><br>Pulmonary Function (abnormal vs. normal): OR=2.10 (95% CI 0.99 to 4.48), p=0.054
**Clinical**	
Preoperative AIS/Neurological status	**Deformity**<br><br>Zhang, 2017 (N=62)<br><br>Pre-operative neurological status (normal vs abnormal): OR=NR, p>0.05<br><br>**Mixed**<br><br>Chen, 2012 (N=316)<br><br>Pre-operative AIS status (A-D; better vs. worse): OR=0.35 (95% CI 0.18 to 0.66), p=0.001

For Peer Review

Combined Myelopathy	**Degeneration**<br><br>Kim, 2021 (N=196)<br><br>Combined myelopathy prior to surgery (yes vs. no): OR=8.24 (95% CI 1.57 to 43.38), p=0.013
Blood loss	**Deformity**<br><br>Fehlings, 2018 (N=265)<br><br>Estimated blood loss (per 500 cc): OR=1.06 (95% CI 0.97 to 1.15), p=0.179<br><br>Zhang, 2017 (N=62)<br><br>Estimated blood loss† (>50% vs. <50%): OR=3.05 (95% CI 1.16 to 8.05), p=0.024<br><br>**Degeneration**<br><br>Kim, 2021 (N=196)<br><br>Estimated blood loss (continuous*): OR=1.00 (95% CI 0.99 to 1.002), p=0.862
**Surgical**	
Number of spinal levels	**Deformity**<br><br>Fehlings, 2018 (N=265)

For Peer Review

	Number of spinal levels involved (per 1 level): OR=1.08 (95% CI 0.99 to 1.17), p=0.091<br><br>**Degeneration**<br><br>Kim, 2021 (N=196)<br><br>Number of levels fused (1 to 2 vs. ≥3): OR=1.36 (95% CI 0.59 to 3.11), p=0.470<br><br>**Mixed**<br><br>Chen, 2012 (N=316)<br><br>Number of involved segments (more vs. less): OR=3.28 (95% CI 1.55 to 6.92), p=0.002
Operation type	**Degeneration**<br><br>Kim, 2021 (N=196)<br><br>Operation type (emergency vs. elective): OR=0.00 (95% CI NR), p=0.999
Operation time	**Degeneration**<br><br>Kim, 2021 (N=196)<br><br>Operation time (continuous*): OR=1.004 (95% CI 0.99 to 1.01), p=0.238
Use of intra-operative monitoring	**Degeneration**<br><br>Kim, 2021 (N=196)

For Peer Review

	Use of intra-operative neuromonitoring (yes vs. no): OR=0.14 (95% CI 0.04 to 0.52), p=0.003
Lumbar-level osteotomy	**Deformity**<br>Fehlings, 2018 (N=265)<br>Lumbar-level osteotomy (yes vs. no): OR=3.30 (95% CI 1.18 to 9.17), p=0.022
Ponte-osteotomy	**Deformity***<br>Buckland, 2018 (N=2210)<br>Ponte osteotomy (yes vs. no): OR=NR, p<0.001
Prevalence of 3 column osteotomy	**Deformity**<br>Fehlings, 2018 (N=265)<br>Prevalence of 3CO (yes vs. no): OR=2.16 (95% CI 0.77 to 6.08), p=0.143
**Radiographic**	
Coronal Deformity Angular Ratio	**Deformity**<br>Fehlings, 2018 (N=265)<br>Coronal deformity angular ratio (per 1 unit): OR=1.10 (95% CI 1.01 to 1.19), p=0.037
Curve Magnitude	**Deformity***<br>Buckland, 2018 (N=2210)<br>Curve magnitude (continuous*) OR=NR, p<0.001

For Peer Review

3CO = 3 Column Osteotomy; CCI = Charlson Comorbidity Index; IQR = Interquartile range; OR = odds ratio; CI = confidence interval; AIS = ASIA Impairment Scale; BMI = body mass index; NR = not reported

* Authors do not report details, variable is assumed to be continuous.

† EBL, the ratio between circulating and lost blood

‡ Risk factors for intra-operative neuromonitoring defined as a reduction in amplitude of 50% or more in SSEPs and/or tcMEPs. Increases in response latency were not included based on prior literature suggesting that it was not an independent sign of neurological injury in spine surgery.

§ Represents patients that received intra-operative monitoring group vs. patients that did not receive intra-operative monitoring.

For Peer Review

Table 10: Strength of evidence (SOE) table assessing risk factors for intra-operative SCI in prognostic studies.

Prognostic factors	No. Studies (no. patients)	Overall Quality	Conclusions*	Study Limitations	Serious Inconsistency	Serious Indirectness	Serious Imprecision	Publication Bias
**Demographic**								
Age	**Deformity**<br>2 (N=327)<br>Fehlings 2018<br>Zhang 2017	MODERATE	**Deformity**<br>Increasing age was associated with increased odds of experiencing ISCI	Low	Unknown†	No	No	Unknown
	**Mixed**<br>2 (N=1,598)<br>Chen 2012	LOW	**Mixed**<br>Increasing age was associated with increased odds in one	Moderate	No	No	No	Unknown

For Peer Review

	Romero-Muñoz 2019		study; no association in a second, larger study					
	**Degeneration** 1 (N=196) Kim 2021	VERY LOW	**Degeneration** Age was not associated with increased odds of experiencing an ISCI	High	Unknown	No	No	Unknown
Sex	**Mixed** 1 (N=316) Chen 2012	LOW	**Mixed** Males had increased odds of experiencing an ISCI	Low	Unknown	No	Yes	Unknown
	**Degeneration** 1 (N=196)	VERY LOW	**Degeneration**	High	Unknown	No	Yes	Unknown

For Peer Review

	Kim 2021		Sex was not associated with increased odds of experiencing an ISCI					
Hypertension	**Mixed**<br>2 (N=1,598)<br>Chen 2012<br>Romero-Muñoz 2019	LOW	**Mixed**<br>Hypertension was not associated with increased odds of experiencing an ISCI in a larger study; hypertension was associated with a large increase in odds in a second study, but confidence intervals were extremely wide	Moderate	Yes	No	Yes	Unknown

For Peer Review

Diabetes	**Mixed**<br><br>1 (N=1,282)<br><br>Romero-Muñoz 2019	LOW	**Mixed**<br><br>Diabetes was not associated with increased odds of experiencing an ISCI	Moderate	Unknown	No	No	Unknown
BMI/Obesity	**Mixed (obesity)**<br><br>1 (N=1,282)<br><br>Romero-Muñoz 2019	LOW	**Mixed**<br><br>BMI/obesity was not associated with increased odds of experiencing an ISCI	Moderate	Unknown	No	Yes	Unknown
	**Degeneration (BMI)**	VERY LOW	**Degeneration**<br><br>BMI/obesity was not associated with	Yes	Unknown	No	No	Unknown

For Peer Review

	1 (N=196)<br><br>Kim 2021		increased odds of<br><br>experiencing an ISCI					
Depression	**Mixed**<br><br>1 (N=1,282)<br><br>Romero-<br><br>Muñoz 2019	LOW	**Mixed**<br><br>Depression was not<br><br>associated with<br><br>increased odds of<br><br>experiencing a ISCI	Moderate	Unknown	No	Yes	Unknown
CCI score	**Degeneration**<br><br>1 (N=196)<br><br>Kim 2021	VERY LOW	**Degeneration**<br><br>CCI scores at baseline<br><br>was not associated with<br><br>increased odds of<br><br>experiencing an ISCI	High	Unknown	No	No	Unknown
Dyslipidemia	**Mixed**<br><br>1 (N=1,282)	LOW	**Mixed**	Moderate	Unknown	No	No	Unknown

For Peer Review

	Romero-Muñoz 2019		Dyslipidemia was not associated with increased odds of experiencing an ISCI					
Pulmonary Function	**Deformity** 1 (N=62) Zhang 2017	VERY LOW	**Deformity** Abnormal pulmonary function was associated with increased odds of experiencing an ISCI	Yes	Unknown	No	Yes	Unknown
**Clinical**								
Preoperative AIS/Neurological status	**Deformity** 1 (N=62) Zhang 2017	VERY LOW	**Deformity** Preoperative neurological status (normal vs. abnormal) was not associated with	Moderate	Unknown	No	Yes (CI not provided)	Unknown

For Peer Review

			increased odds of experiencing an ISCI					
	**Mixed**‡<br>1 (N=316)<br>Chen 2012	MODERATE	**Mixed**‡<br>Better preoperative AIS was associated with decreased odds of experiencing an ISCI	Low	Unknown	No	No	Unknown
Combined Myelopathy	**Degeneration**<br>1 (N=196)<br>Kim 2021	VERY LOW	**Degeneration**<br>Presence of compressive myelopathy prior to surgery was associated with a large, increased odds of experiencing	High	Unknown	No	Yes	Unknown

For Peer Review

			an ISCI; confidence interval was extremely wide					
Estimated blood loss	**Deformity**<br><br>2 Studies (N=327)<br><br>Fehlings 2018<br><br>Zhang 2017	MODERATE	**Deformity**<br><br>EBL (per 500 cc) was not associated with increased odds of experiencing an ISCI in the larger, good-quality study; the fair-quality study found EBL ≥50% vs. <50% (i.e., ratio between circulating and lost blood) was associated with increased odds of	Low	Unknown[§]	No	No	Unknown

For Peer Review

			experiencing an ISCI, but confidence interval was wide.					
	**Degeneration** 1 (N=196) Kim 2021	VERY LOW	**Degeneration** EBL (continuous) was not associated with increased odds of experiencing an ISCI	High	Unknown	No	No	Unknown
**Surgical**								
Number of spinal levels	**Deformity** 1 (N=265) Fehlings 2018	MODERATE	**Deformity** Number of spinal levels involved (1 per level) was not associated with	Low	Unknown	No	No	Unknown

For Peer Review

			increased odds of experiencing an ISCI					
	**Degeneration** 1 (N=196) Kim 2021	VERY LOW	**Degeneration** Number of levels fused (continuous) was not associated with increased odds of experiencing an ISCI	High	Unknown	No	Yes	Unknown
	**Mixed** 1 (N=316) Chen 2012	LOW	**Mixed** Increasing number of involved segments was associated with a large increased odds of	Low	Unknown	No	Yes	Unknown

For Peer Review

			experiencing an ISCI, but the confidence interval was wide					
Lumbar-level osteotomy	**Deformity** 1 (N=265) Fehlings 2018	LOW	**Deformity** Lumbar-level osteotomy was associated with large increased odds of experiencing an ISCI, but the confidence interval was wide	Low	Unknown	No	Yes	Unknown
Ponte-osteotomy	**Deformity** 1 (N=2,210) Buckland 2018	VERY LOW	**Deformity** Ponte-osteotomy was associated with	High	Unknown	No	Yes (OR/CI not provided)	Unknown

For Peer Review

			increased odds of an IONM alert					
Prevalence of 3CO	**Deformity** 1 (N=265) Fehlings 2018	LOW	**Deformity** 3-column osteotomy was not associated with increased odds of experiencing an ISCI; the confidence interval was wide	Low	Unknown	No	Yes	Unknown
Operation type	**Degeneration** 1 (N=196) Kim 2021	VERY LOW	**Degeneration** Operation type (emergency vs. elective) was not associated with	High	Unknown	No	Yes (CI not provided)	Unknown

For Peer Review

			increased odds of experiencing an ISCI					
Operation time	**Degeneration**<br>1 (N=196)<br>Kim 2021	VERY LOW	**Degeneration**<br>Operation time (continuous) was not associated with increased odds of experiencing an ISCI	High	Unknown	No	No	Unknown
Use of intra-operative monitoring	**Degeneration**<br>1 (N=196)<br>Kim 2021	VERY LOW	**Degeneration**<br>Use of intra-operative monitoring was associated with a large decrease in odds of experiencing an ISCI	High	Unknown	No	No	Unknown
**Radiographic**								

For Peer Review

Coronal DAR	**Deformity**<br><br>1 (N=265)<br><br>Fehlings 2018	MODERATE	**Deformity**<br><br>Coronal DAR (per 1 unit) was associated with increased odds of experiencing an ISCI	Low	Unknown	No	No	Unknown
Curve Magnitude	**Deformity**<br><br>1 (N=2,210)<br><br>Buckland 2018	VERY LOW	**Deformity**<br><br>Curve magnitude (coronal and sagittal Cobb) was associated with increased odds of IONM alerts	High	Unknown	No	Yes<br><br>(OR/CI not provided)	Unknown

3CO = 3 Column Osteotomy; CCI = Charlson Comorbidity Index; CI = confidence interval; DAR = Deformity Angular Ratio; OR = odds ratio

* Conclusions addressed association between prognostic factor and increased risk of intra-operative/immediate postoperative neurological deficit or decline (measured via ASIA grade) in all studies except for Buckland 2018 which evaluated the association is between the factor and the risk of intra-operative monitoring alerts.

For Peer Review

† Age modeled differently: Fehlings et al. evaluated age per 10-year increments and Zhang et al. evaluated age dichotomized into ≥18 vs. <18 years. Also, the mean age of the study populations differed: 56.8 years (SD 15.4, range 18–81) for Fehlings et al. and 16.3 years (SD 6.4, range 6–46 years) for Zhang et al.

‡ Large proportion (almost 50%) of patients had AIS grade B.

§ Different ways of measuring estimated blood loss in the two studies: per 500 cc (Fehlings et al.) and as the ratio between circulating and lost blood dichotomized into ≥50% vs. <50% (Zhang et al.). Populations also appear to be different.

For Peer Review

Table 11: Factors which increase the risk for intra-operative spinal cord injury.

High Risk Deformity: Rigid Thoracic Curve with High deformity Angular Ratio (DAR)
Revision Congenital Deformity with Significant Cord Compression & Myelopathy
Extrinsic Lesion with Cord Compression & Myelopathy
Intramedullary Tumor
Unstable Fractures: B/L Facet Dislocation and Disc Herniation
Extension Distraction Injury with Ankylosing Spondylitis
OPLL with severe cord compression and moderate to severe myelopathy

For Peer Review

Appendix A. Excluded studies.

List of Select Excluded Studies and Rationale

	Citation	Reason for exclusion
1	Ahn H, Fehlings MG. Prevention, identification, and treatment of perioperative spinal cord injury. Neurosurg Focus. 2008;25(5):E15. doi: 10.3171/FOC.2008.25.11.E15. PMID: 18980475.	Does not use multivariable regression to assess risk factors
2	Alosh H, Parker SL, McGirt MJ, Gokaslan ZL, Witham TF, Bydon A, Wolinsky JP, Sciubba DM. Preoperative radiographic factors and surgeon experience are associated with cortical breach of C2 pedicle screws. J Spinal Disord Tech. 2010 Feb;23(1):9-14. doi: 10.1097/BSD.0b013e318194e746. PMID: 20068474.	Does not use multivariable regression to assess risk factors
3	Bejjani GK, Nora PC, Vera PL, Broemling L, Sekhar LN. The predictive value of intraoperative somatosensory evoked potential monitoring: review of 244 procedures. Neurosurgery. 1998 Sep;43(3):491-8; discussion 498-500. doi: 10.1097/00006123-199809000-00050. PMID: 9733304.	Ineligible study design for Key Question, e.g., case series, modeling (e.g., prediction models, thresholds/ROC, etc.)
4	Bridwell KH, Lenke LG, Baldus C, Blanke K. Major intraoperative neurologic deficits in pediatric and adult spinal deformity patients. Incidence and etiology at one institution. Spine (Phila Pa 1976). 1998 Feb 1;23(3):324-31. doi: 10.1097/00007632-199802010-00008. PMID: 9507620.	Does not use multivariable regression to assess risk factors

For Peer Review

5	Chen J, Shao XX, Sui WY, Yang JF, Deng YL, Xu J, Huang ZF, Yang JL. Risk factors for neurological complications in severe and rigid spinal deformity correction of 177 cases. BMC Neurol. 2020 Nov 28;20(1):433. doi: 10.1186/s12883-020-02012-8. PMID: 33246421; PMCID: PMC7697368.	Does not use multivariable regression to assess risk factors
6	Clark AJ, Ziewacz JE, Safaee M, Lau D, Lyon R, Chou D, Weinstein PR, Ames CP, Clark JP 3rd, Mummaneni PV. Intraoperative neuromonitoring with MEPs and prediction of postoperative neurological deficits in patients undergoing surgery for cervical and cervicothoracic myelopathy. Neurosurg Focus. 2013 Jul;35(1):E7. doi: 10.3171/2013.4.FOCUS13121. PMID: 23815252.	Does not use multivariable regression to assess risk factors
7	De la Garza Ramos R, Goodwin CR, Abu-Bonsrah N, Jain A, Miller EK, Huang N, Kebaish KM, Sponseller PD, Sciubba DM. Patient and operative factors associated with complications following adolescent idiopathic scoliosis surgery: an analysis of 36,335 patients from the Nationwide Inpatient Sample. J Neurosurg Pediatr. 2016 Dec;25(6):730-736. doi: 10.3171/2016.6.PEDS16200. Epub 2016 Aug 26. PMID: 27564784.	Ineligible population
8	Feng B, Qiu G, Shen J, Zhang J, Tian Y, Li S, Zhao H, Zhao Y. Impact of multimodal intraoperative monitoring during surgery for spine deformity and potential risk factors for neurological monitoring changes. J Spinal Disord Tech. 2012 Jun;25(4):E108-14. doi: 10.1097/BSD.0b013e31824d2a2f. PMID: 22367467.	Does not use multivariable regression to assess risk factors

For Peer Review

9	Ghadirpour R, Nasi D, Iaccarino C, Romano A, Motti L, Sabadini R, Valzania F, Servadei F. Intraoperative neurophysiological monitoring for intradural extramedullary spinal tumors: predictive value and relevance of D-wave amplitude on surgical outcome during a 10-year experience. J Neurosurg Spine. 2018 Nov 9;30(2):259-267. doi: 10.3171/2018.7.SPINE18278. PMID: 30497134.	Ineligible study design for Key Question, e.g., case series, modeling (e.g., prediction models, thresholds/ROC, etc.)
10	Glennie RA, Ailon T, Yang K, Batke J, Fisher CG, Dvorak MF, Vaccaro AR, Fehlings MG, Arnold P, Harrop JS, Street JT. Incidence, impact, and risk factors of adverse events in thoracic and lumbar spine fractures: an ambispective cohort analysis of 390 patients. Spine J. 2015 Apr 1;15(4):629-37. doi: 10.1016/j.spinee.2014.11.016. Epub 2014 Nov 28. PMID: 25450658.	Ineligible study design for Key Question, e.g., case series, modeling (e.g., prediction models, thresholds/ROC, etc.)
11	Guest JD, Vanni S, Silbert L. Mild hypothermia, blood loss and complications in elective spinal surgery. Spine J. 2004 Mar-Apr;4(2):130-7. doi: 10.1016/j.spinee.2003.08.027. PMID: 15016389.	Does not use multivariable regression to assess risk factors
12	Huang ZF, Chen L, Yang JF, Deng YL, Sui WY, Yang JL. Multimodality Intraoperative Neuromonitoring in Severe Thoracic Deformity Posterior Vertebral Column Resection Correction. World Neurosurg. 2019 Jul;127:e416-e426. doi: 10.1016/j.wneu.2019.03.140. Epub 2019 Apr 11. PMID: 30981802.	Does not use multivariable regression to assess risk factors
13	Kashkoush A, Mehta A, Agarwal N, Nwachuku EL, Fields DP, Alan N, Kanter AS, Okonkwo DO, Hamilton DK, Thirumala PD. Perioperative Neurological Complications Following Anterior Cervical Discectomy and Fusion: Clinical Impact on 317,789 Patients from the National Inpatient Sample. World Neurosurg. 2019	Ineligible study design for Key Question, e.g., case series, modeling (e.g., prediction models, thresholds/ROC, etc.), ineligible population

For Peer Review

	Aug;128:e107-e115. doi: 10.1016/j.wneu.2019.04.037. Epub 2019 Apr 10. PMID: 30980979.	
14	Kato S, Fehlings MG, Lewis SJ, Lenke LG, Shaffrey CI, Cheung KMC, Carreon LY, Dekutoski MB, Schwab FJ, Boachie-Adjei O, Kebaish KM, Ames CP, Qiu Y, Matsuyama Y, Dahl BT, Mehdian H, Pellisé F, Berven SH. An Analysis of the Incidence and Outcomes of Major Versus Minor Neurological Decline After Complex Adult Spinal Deformity Surgery: A Subanalysis of Scoli-RISK-1 Study. Spine (Phila Pa 1976). 2018 Jul 1;43(13):905-912. doi: 10.1097/BRS.0000000000002486. PMID: 29894429.	Does not use multivariable regression to assess risk factors
15	Kelly MP, Lenke LG, Godzik J, Pellise F, Shaffrey CI, Smith JS, Lewis SJ, Ames CP, Carreon LY, Fehlings MG, Schwab F, Shimer AL. Retrospective analysis underestimates neurological deficits in complex spinal deformity surgery: a Scoli-RISK-1 Study. J Neurosurg Spine. 2017 Jul;27(1):68-73. doi: 10.3171/2016.12.SPINE161068. Epub 2017 May 5. PMID: 28475019.	Does not use multivariable regression to assess risk factors
16	Kim DH, Zaremski J, Kwon B, Jenis L, Woodard E, Bode R, Banco RJ. Risk factors for false positive transcranial motor evoked potential monitoring alerts during surgical treatment of cervical myelopathy. Spine (Phila Pa 1976). 2007 Dec 15;32(26):3041-6. doi: 10.1097/BRS.0b013e31815d0072. PMID: 18091499.	Does not use multivariable regression to assess risk factors
17	Lee JY, Hilibrand AS, Lim MR, Zavatsky J, Zeiller S, Schwartz DM, Vaccaro AR, Anderson DG, Albert TJ. Characterization of neurophysiologic alerts during anterior	Does not use multivariable regression to assess risk factors

For Peer Review

	cervical spine surgery. Spine (Phila Pa 1976). 2006 Aug 1;31(17):1916-22. doi: 10.1097/01.brs.0000228724.01795.a2. PMID: 16924208.	
18	Lenke LG, Fehlings MG, Shaffrey CI, Cheung KM, Carreon L, Dekutoski MB, Schwab FJ, Boachie-Adjei O, Kebaish KM, Ames CP, Qiu Y, Matsuyama Y, Dahl BT, Mehdian H, Pellisé-Urquiza F, Lewis SJ, Berven SH. Neurologic Outcomes of Complex Adult Spinal Deformity Surgery: Results of the Prospective, Multicenter Scoli-RISK-1 Study. Spine (Phila Pa 1976). 2016 Feb;41(3):204-12. doi: 10.1097/BRS.0000000000001338. PMID: 26866736.	Does not use multivariable regression to assess risk factors
19	Lewis ND, Keshen SG, Lenke LG, Zywiel MG, Skaggs DL, Dear TE, Strantzas S, Lewis SJ. The Deformity Angular Ratio: Does It Correlate With High-Risk Cases for Potential Spinal Cord Monitoring Alerts in Pediatric 3-Column Thoracic Spinal Deformity Corrective Surgery? Spine (Phila Pa 1976). 2015 Aug 1;40(15):E879-85. doi: 10.1097/BRS.0000000000000984. PMID: 26222664.	Does not use multivariable regression to assess risk factors
20	Lewis SJ, Gray R, Holmes LM, Strantzas S, Jhaveri S, Zaarour C, Magana S. Neurophysiological changes in deformity correction of adolescent idiopathic scoliosis with intraoperative skull-femoral traction. Spine (Phila Pa 1976). 2011 Sep 15;36(20):1627-38. doi: 10.1097/BRS.0b013e318216124e. PMID: 21897186.	Does not use multivariable regression to assess risk factors
21	Montalva-Iborra A, Alcanyis-Alberola M, Grao-Castellote C, Torralba-Collados F, Giner-Pascual M. Risk factors in iatrogenic spinal cord injury. Spinal Cord. 2017 Sep;55(9):818-822. doi: 10.1038/sc.2017.21. Epub 2017 Apr 4. PMID: 28374810.	Does not use multivariable regression to assess risk factors

For Peer Review

22	Park T, Park J, Park YG, Lee J. Intraoperative Neurophysiological Monitoring for Spinal Cord Tumor Surgery: Comparison of Motor and Somatosensory Evoked Potentials According to Tumor Types. Ann Rehabil Med. 2017 Aug;41(4):610-620. doi: 10.5535/arm.2017.41.4.610. Epub 2017 Aug 31. PMID: 28971046; PMCID: PMC5608669.	Does not use multivariable regression to assess risk factors
23	Rocos B, Strantzas S, Zeller R, Lewis S, Tan T, Lebel D. What is the Optimal Surgical Method for Achieving Correction and Avoiding Neurological Complications in Pediatric High-grade Spondylolisthesis? J Pediatr Orthop. 2021 Mar 1;41(3):e217-e225. doi: 10.1097/BPO.0000000000001707. PMID: 33165266.	Does not use multivariable regression to assess risk factors
24	Sadashivam S, Abraham M, Kesavapisharady K, Nair SN. Long-term outcome and prognostic factors of intramedullary spinal hemangioblastomas. Neurosurg Rev. 2020 Feb;43(1):169-175. doi: 10.1007/s10143-018-1025-2. Epub 2018 Aug 31. PMID: 30171501.	Does not use multivariable regression to assess risk factors
25	Saiwai H, Okada S, Hayashida M, Harimaya K, Matsumoto Y, Kawaguchi KI, Kobayakawa K, Maeda T, Ohta H, Shirasawa K, Tsuchiya K, Terada K, Kaji K, Arizono T, Saito T, Fujiwara M, Iwamoto Y, Nakashima Y. Surgery-related predictable risk factors influencing postoperative clinical outcomes for thoracic myelopathy caused by ossification of the posterior longitudinal ligament: a multicenter retrospective study. J Neurosurg Spine. 2019 Dec 27:1-7. doi: 10.3171/2019.10.SPINE19831. Epub ahead of print. PMID: 31881534.	Does not use multivariable regression to assess risk factors

For Peer Review

26	Shlobin NA, Raz E, Shapiro M, Clark JR, Hoffman SC, Shaibani A, Hurley MC, Ansari SA, Jahromi BS, Dahdaleh NS, Potts MB. Spinal neurovascular complications with anterior thoracolumbar spine surgery: a systematic review and review of thoracolumbar vascular anatomy. Neurosurg Focus. 2020 Sep;49(3):E9. doi: 10.3171/2020.6.FOCUS20373. PMID: 32871559.	Does not use multivariable regression to assess risk factors
27	Thuet ED, Padberg AM, Raynor BL, Bridwell KH, Riew KD, Taylor BA, Lenke LG. Increased risk of postoperative neurologic deficit for spinal surgery patients with unobtainable intraoperative evoked potential data. Spine (Phila Pa 1976). 2005 Sep 15;30(18):2094-103. doi: 10.1097/01.brs.0000178845.61747.6a. PMID: 16166902.	Does not use multivariable regression to assess risk factors
28	Toll BJ, Samdani AF, Janjua MB, Gandhi S, Pahys JM, Hwang SW. Perioperative complications and risk factors in neuromuscular scoliosis surgery. J Neurosurg Pediatr. 2018 Aug;22(2):207-213. doi: 10.3171/2018.2.PEDS17724. Epub 2018 May 11. PMID: 29749884.	Does not use multivariable regression to assess risk factors
29	Ushirozako H, Yoshida G, Hasegawa T, Yamato Y, Yasuda T, Banno T, Arima H, Oe S, Yamada T, Ide K, Watanabe Y, Kurita T, Matsuyama Y. Characteristics of false-positive alerts on transcranial motor evoked potential monitoring during pediatric scoliosis and adult spinal deformity surgery: an "anesthetic fade" phenomenon. J Neurosurg Spine. 2019 Nov 22:1-9. doi: 10.3171/2019.9.SPINE19814. Epub ahead of print. PMID: 31756712.	Does not use multivariable regression to assess risk factors
30	Vitale MG, Moore DW, Matsumoto H, Emerson RG, Booker WA, Gomez JA, Gallo EJ, Hyman JE, Roye DP Jr. Risk factors for spinal cord injury during surgery for spinal	Does not use multivariable regression to assess risk factors

For Peer Review

	deformity. J Bone Joint Surg Am. 2010 Jan;92(1):64-71. doi: 10.2106/JBJS.H.01839. PMID: 20048097.	
31	Watanabe T, Kanayama M, Takahata M, Oda I, Suda K, Abe Y, Okumura J, Hojo Y, Iwasaki N. Perioperative complications of spine surgery in patients 80 years of age or older: a multicenter prospective cohort study. J Neurosurg Spine. 2019 Dec 17:1-9. doi: 10.3171/2019.9.SPINE19754. Epub ahead of print. PMID: 31846935.	Does not use multivariable regression to assess risk factors
32	Wilson TJ, Hamrick F, Alzahrani S, Dibble CF, Koduri S, Pendleton C, Saleh S, Ali ZS, Mahan MA, Midha R, Ray WZ, Yang LJS, Zager EL, Spinner RJ. Analysis of the effect of intraoperative neuromonitoring during resection of benign nerve sheath tumors on gross-total resection and neurological complications. J Neurosurg. 2021 Feb 12:1-10. doi: 10.3171/2020.8.JNS202885. Epub ahead of print. PMID: 33578389.	Ineligible study design for Key Question, e.g., case series, modeling (e.g., prediction models, thresholds/ROC, etc.)
33	Yoo M, Park YG, Cho YE, Lim CH, Chung SY, Kim D, Park J. Intraoperative evoked potentials in patients with ossification of posterior longitudinal ligament. J Clin Monit Comput. 2022 Feb;36(1):247-258. doi: 10.1007/s10877-020-00646-0. Epub 2021 Feb 6. PMID: 33548015.	Does not use multivariable regression to assess risk factors

For Peer Review
