# Supplementary material for: The 2023 AO Spine-Praxis Guidelines in Acute Spinal Cord Injury: What Have We Learned? What Are the Critical Knowledge Gaps and Barriers to Implementation?
Source: Global Spine J. 2024 Mar 25;14(3 Suppl):223S–230S. doi: 10.1177/21925682231196825 (PMC10964887; doi:10.1177/21925682231196825)
Supplement: Supplemental Material - The 2023 AO Spine-Praxis Guidelines in Acute Spinal Cord Injury: What Have We Learned? What Are the Critical Knowledge Gaps and Barriers to Implementation? [file sj-pdf-2-gsj-10.1177_21925682231196825.pdf]

An Update of a Clinical Practice Guideline for the Management of Patients with Acute Spinal Cord Injury: Recommendations on the Role and Timing of Decompressive Surgery

Journal:	*Global Spine Journal*
Manuscript ID	Draft
Manuscript Type:	Special Issue- Knowledge Forum SCI Guidelines
Keywords:	spinal cord injury, decompression, trauma

Abstract:

Study Design
Clinical practice guideline development

Objectives
Acute spinal cord injury (SCI) can result in devastating motor, sensory and autonomic impairment, loss of independence and reduced quality of life. Preclinical evidence suggests that early decompression of the spinal cord may help to limit secondary injury, reduce damage to the neural tissue and improve functional outcomes. Emerging evidence indicates that "early" surgical decompression completed within 24 hours of injury also improves neurological recovery in patients with acute SCI. The objective of this clinical practice guideline (CPG) is to update the 2017 recommendations on the timing of surgical decompression and to evaluate the evidence with respect to ultra-early surgery (in particular, but not limited to <12 hrs after acute SCI).

Methods
A multidisciplinary, international, guideline development group (GDG) was formed that consisted of spine surgeons, neurologists, critical care specialists, emergency medicine doctors, physical medicine and rehabilitation professionals and individuals living with SCI. A systematic review was conducted based on accepted methodological standards to evaluate the impact of early (within 24 hours of acute SCI) or ultra-early (in particular, but not limited to within 12 hours of acute SCI) surgery on neurologic recovery, functional outcomes, administrative outcomes, safety and cost-effectiveness. The GRADE approach was used to rate the overall strength of evidence across studies for each primary outcome. Using the "evidence-to-recommendation" framework, recommendations were then developed that considered the balance of benefits and harms, financial impact, patient values, acceptability and feasibility. The guideline was internally appraised using the...

SCHOLARONE™
Manuscripts

Abstract

Study Design

Clinical practice guideline development

Objectives

Acute spinal cord injury (SCI) can result in devastating motor, sensory and autonomic impairment, loss of independence and reduced quality of life. Preclinical evidence suggests that early decompression of the spinal cord may help to limit secondary injury, reduce damage to the neural tissue and improve functional outcomes. Emerging evidence indicates that “early” surgical decompression completed within 24 hours of injury also improves neurological recovery in patients with acute SCI. The objective of this clinical practice guideline (CPG) is to update the 2017 recommendations on the timing of surgical decompression and to evaluate the evidence with respect to ultra-early surgery (in particular, but not limited to <12 hrs after acute SCI).

Methods

A multidisciplinary, international, guideline development group (GDG) was formed that consisted of spine surgeons, neurologists, critical care specialists, emergency medicine doctors, physical medicine and rehabilitation professionals and individuals living with SCI. A systematic review was conducted based on accepted methodological standards to evaluate the impact of early (within 24 hours of acute SCI) or ultra-early (in particular, but not limited to within 12 hours of acute SCI) surgery on neurologic recovery, functional outcomes, administrative outcomes, safety and cost-effectiveness. The GRADE approach was used to rate the overall strength of evidence across studies for each primary outcome. Using the “evidence-to-recommendation” framework, recommendations were then developed that considered the balance of benefits and harms, financial impact, patient values, acceptability and feasibility. The guideline was internally appraised using the Appraisal of Guidelines for Research and Evaluation (AGREE) II tool.

Results

The GDG recommended that early surgery (≤24 hours after injury) be offered as the preferred option for adult patients with acute SCI regardless of level. This recommendation was based on moderate evidence that suggested that patients were two times more likely to recover by ≥2 ASIA Impairment Score (AIS) grades at 6- (RR: 2.76, 95% CI 1.60 to 4.98) and 12-months (RR: 1.95, 95% CI 1.26 to 3.18) if they were decompressed within 24 hours compared to after 24 hours. Furthermore, patients undergoing early surgery improved by an additional 4.50 (95% 1.70 to 7.29) points on the ASIA Motor Score compared to

For Peer Review

patients undergoing surgery after 24 hours post-injury. The GDG also agreed that a recommendation for ultra-early surgery could not be made on the basis of the current evidence because of the small sample sizes, variable definitions of what constituted ultra-early in the literature and the inconsistency of the evidence.

Conclusions

It is recommended that patients with an acute SCI, regardless of level, undergo surgery within 24 hrs after injury when medically feasible. Future research is required to determine the differential effectiveness of early surgery in different subpopulations and the impact of ultra-early surgery on neurological recovery. Moreover, further work is required to define what constitutes effective spinal cord decompression and to individualize care. It is also recognized that a concerted international effort will be required to translate these recommendations into policy.

For Peer Review

Summary of Recommendations

Population Description: Adult patients with acute SCI

Key Question 1: Should we recommend early decompressive surgery (≤24 hours after injury) for adult patients with acute spinal cord injury regardless of injury severity and neurological level?

Recommendation: We recommend that early surgery be offered as an option for adult patients with acute SCI regardless of level.

Quality of Evidence: Moderate

Strength of Recommendation: Strong

Population: Adult patients with acute SCI

Key Question 2: Should we recommend ultra-early decompressive surgery for adult patients with acute SCI regardless of injury severity and neurological level?

Statement: A recommendation for ultra-early surgery could not be made on the basis of the current evidence because of the small sample sizes, variable definitions of what constituted ultra-early and the inconsistency of the evidence.

For Peer Review

Introduction

Traumatic spinal cord injury (SCI) can result in devastating motor, sensory and autonomic impairment, loss of independence and reduced quality of life[1]. The acute and long-term management of SCI requires substantial health care resources and can impose significant financial burden on patients, families and communities[2]. The treatment of SCI has evolved over time due to the emergence of several preclinical and clinical studies on injury mechanisms, disease pathophysiology, the role of surgical intervention and novel neuroprotective strategies.

Primary injury refers to the initial mechanical forces applied to the spinal cord by vertebral fractures, disc debris or disruption of the supporting ligaments[3,4]. Within minutes to hours of injury, significant pathophysiological changes occur that can damage the ascending and descending neural pathways and induce spinal shock[5,6]. These changes include vasospasm, ischemia, disruption of the blood-spinal cord barrier, ion imbalance and accumulation of neurotransmitters[5]. Secondary injury begins within minutes of the initial trauma and may cause progressive spinal cord damage for weeks to

months through demyelination, Wallerian degeneration, and formation of a glial scar[4]. Preclinical evidence suggests that early decompression of the spinal cord may help to limit this secondary injury, reduce damage to the neural tissue and improve functional outcomes[7-9]. This concept of timely surgical intervention has also been investigated by a number of clinical trials and cohort studies, allowing for the development of an evidence-based clinical practice guideline (CPG) in 2017[10].

This 2017 CPG provided recommendations for the timing of surgical intervention in patients with SCI, using 24 hours as the threshold between early and late decompression[10]. Based on a systematic review of the literature and a guideline development framework, a weak recommendation was formulated to suggest early surgery be offered as an option for adult SCI patients regardless of injury level[10,11]. Since the publication of this CPG, several studies have emerged that assess the impact of early versus late surgical decompression on neurologic recovery, functional outcomes and quality of life[12-21]. Given the availability of new evidence, it is advised that the CPG from 2017 be updated as the results of recent studies may change the strength of previous recommendations and impact clinical decision-making. Furthermore, as care pathways for patients with SCI become more streamlined, it may be possible to intervene even earlier, especially if beneficial. It is therefore the endeavor of this CPG to explore different time cut-offs (e.g. <4, <8 or <12 hours) in order to establish the ideal timing of surgical intervention in terms of impact on outcome, cost-effectiveness and feasibility.

The overarching aim of this CPG is to standardize care, improve outcomes and reduce morbidity and mortality in patients with SCI by creating recommendations on the timing of surgical intervention. The specific aims of this CPG are to address the following two questions:

1. Should we recommend early decompressive surgery (≤24 hours after injury) for adult patients with acute spinal cord injury regardless of injury severity and neurological level?
2. Should we recommend very early decompressive surgery for adult patients with acute spinal cord injury regardless of injury severity and neurological level?

Methods

The development of this CPG was sponsored by AO Spine and Praxis Spinal Cord Institute. A multidisciplinary guideline development group (GDG) was established that consisted of neurosurgeons, orthopedic surgeons, neurologists, critical care specialists, emergency medicine doctors, first responders, physical medicine and rehabilitation professionals and individuals living with SCI. Each member of the

For Peer Review

GDG was required to disclose both financial and intellectual conflicts of interest. Methodologists at Aggregate Analytics Inc. were responsible for providing the expertise for both the systematic review of the literature and the development of the CPG. The protocol for this CPG was formulated using the Conference on Guideline Standardization (COGS) framework and the Checklist for Reporting the Updating Process (CheckUp) and is published in a separate article in this focus issue[22,23].

A systematic review was conducted based on accepted methodological standards to address the following key questions:

1. What is the evidence for the effectiveness of early decompression (≤ 24 hours) compared with late decompression (>24 hours) or conservative therapy based on clinically important change in neurological status? What is the effectiveness of ultra-early decompression compared with other "early" time frames up to 24 hours (for example, but not limited to < 12 hours vs ≥ 12 hours but <24 hours)?
2. Does timing of decompression influence other functional outcomes or administrative outcomes?
3. What is the safety profile of early decompression compared with late decompression?
4. Does early decompression have differential efficacy or safety in specific subgroups of patients?
5. What is the cost-effectiveness of early decompression compared with late decompression?

The approach outlined by the Grading of Recommendation, Assessment, Development and Evaluation (GRADE) Working Group was used to rate the overall strength of evidence across studies for each primary outcome[24]. The results of the systematic review and the evidence tables were presented to the GDG. The GRADE "evidence-to-recommendation" framework was used to support the guideline development process and ensure that each recommendation considered other factors, including benefits and harms, resource use, impact on health inequities, acceptability and feasibility[25-27]. The last step in the framework was to balance the desirable and undesirable consequences and determine the strength of each recommendation. The Appraisal of Guidelines for Research and Evaluation (AGREE) II was used to internally appraise the CPG[28]. Finally, this document was distributed to a multidisciplinary group of clinicians as well as prominent societies in spine surgery for external review. A complete summary of the methods used to create this CPG are provided in a separate article in this focus issue entitled "An Overview of the Methodology Used to Develop Clinical Practice Guidelines for the Management of Acute and Intraoperative Spinal Cord Injury."

Clinical Recommendations

For Peer Review

Part 1. Timing of Decompressive Surgery (≤ 24 hours After Injury) in Adult Patients with Acute Spinal Cord Injury

Population Description: Adult patients with acute SCI.

Key Question 1: Should we recommend early decompressive surgery (≤24 hours after injury) for adult patients with acute spinal cord injury regardless of injury severity and neurological level?

Recommendation: We recommend that early surgery be offered as an option for adult patients with acute SCI regardless of level.

Quality of Evidence: Moderate

Strength of Recommendation: Strong

For Peer Review

Evidence Summary

A systematic review of the literature was conducted in order to summarize and appraise the available evidence on the effectiveness and safety of early (≤24 hours post-injury) surgical decompression compared to late (>24 hours post-injury) surgical decompression after acute SCI.

Evidence Related to Benefits

Nine studies reported on change in ASIA Impairment Scale (AIS) at 6- or 12-months following injury[13,15,16,19-21,29-31]. Of these, five studies compared the rate of improvement by ≥2 AIS grades at 6-months between patients undergoing early versus late surgery [20,21,29,30,32]. Based on pooled estimates, patients were 2.76 (95% CI 1.60 to 4.98) times more likely to recover by ≥2 AIS grades if they were decompressed within 24 hours compared to after 24 hours. Four studies evaluated the impact of timing of surgery on improving by ≥2 AIS grades at 12-months[13,15,16,19]. Similarly, patients were approximately two (95% CI 1.26 to 3.18,) times more likely to exhibit a ≥2 grade improvement on the AIS at 12-months if treated within 24 hours compared to after 24 hours. The overall strength of evidence for these outcomes (≥2 grade improvement in AIS at 6- and 12-months) was moderate.

Five studies explored the change in ASIA motor score (AMS) between individuals operated on early versus late[12,14,16,31,32]. The results from the two studies that reported on AMS at 6-months could not be pooled due to substantial clinical heterogeneity and limited reporting of data[31,32]. A single study identified that patients undergoing early (<24 hours post-injury) surgery for acute central cord

syndrome gained an additional 7.47 points on the AMS compared to those treated late[31]. A second study demonstrated that early surgery confers a 13-point improvement in AMS in individuals with cervical, thoracic or lumbosacral SCI[32]. Based on the results of these two studies, there is very low evidence that early surgery improves AMS in the short term. The remaining three studies explored change in AMS at >6-12 months and were evaluated in a meta-analysis[14,16,31]. Based on pooled estimates, patients undergoing early surgery improved by an additional 4.50 (95% 1.70 to 7.29) points on the AMS compared to patients treated late. The overall strength of evidence was moderate for this outcome. In summary, the evidence is very low that early surgery impacts AMS in the short term (<6 months) but moderate that it results in motor improvement in the longer term (>6-12 months).

A single prospective observational study evaluated the impact of early decompression on Functional Independence Measure (FIM) motor and total scores [31]. Based on the results, patients undergoing early surgery for acute central cord syndrome were more likely to exhibit improvements in Functional Independence Measure (FIM) total scores (MD 7.79, 95% CI 0.09 to 15.49, p=0.0474) than those treated late[31]. However, the overall level of evidence for this outcome was rated as very low due to serious imprecision in the estimate of effect.

Evidence Related to Harms

Several studies discussed rates of complications between patients treated surgically within 24 hours of injury and those decompressed after 24 hours [13,15,16,20,21,29,30,33]. Based on the results of the systematic review, there were no statistically significant differences in critical harm outcomes between early and late surgery groups. Furthermore, rates of the majority of reported complications tended to be higher in the late surgery group, including mortality, decubitus ulcers, cardiopulmonary events and need for tracheostomy[15,16,20,29,30,33]. In contrast, the frequency of neurological deterioration and sepsis secondary to systemic infection tended to be higher in the early surgery groups[13,29,30]. Unfortunately, the majority of studies were underpowered to detect a difference in rates of complications based on timing of surgery.

Based on a single study and moderate evidence, there was no difference in the rate of major complications between early (24.2%) and late surgery (30.5%, RR 0.79, 95% CI 0.55 to 1.14)[29]. Two randomized controlled trials and four observational studies evaluated mortality rates in patients treated early versus late[16,20,29,30,33]. Mortality was uncommon and did not significantly differ between groups: 2.6% in

For Peer Review

the early group and 3.6% in the late group (RR 0.68, 95% CI 0.29 to 1.50). Although results across studies were consistent, the overall quality of evidence was rated as low due to moderate risk of bias and imprecision. Three studies examined differences in rates of fixation or construct failure and one study assessed differences in rates of screw revision or pull-out according to timing of surgery[16,20,29]. Based on low evidence, there were no differences in rates of surgical-device related complications between patients decompressed early compared to those treated late (fixation or construct failure: 1.5% vs 1.4%, RR 1.21, 95% CI 0.21 to 5.87; revision of lateral screws or screw pull-out: 8.1% vs 8.3%, RR 0.97, 95% CI 0.21 to 4.51). Based on low evidence, there were no differences in the frequency of sepsis between patients treated early (1.7%) compared to late (0.8%, RR 1.96, 95% CI 0.50 to 7.60)[15,29]. Four studies assessed the rate of decubitus or pressure ulcers based on timing of surgery and concluded that patients treated early had fewer decubitus or pressure ulcers than those treated late (3.8% vs. 6.9%, RR 0.81, 95% CI 0.46 to 1.37)[15,16,20,33]. However, this difference did not reach statistical significance and the overall rating of the evidence was low due to moderate risk of bias. Three studies reported no difference in the rate of neurological deterioration following early (4.7%) versus late (0.7%) surgery in patients with cervical SCI (RR 3.51, 95% CI 0.73 to 17.23)[13,29,30]. The overall rating of the evidence for this outcome was very low. Finally, based on the results of single studies and low evidence, there were fewer cardiopulmonary complications (17.6% vs. 25.9%, RR 0.68, 95% CI 0.44 to 1.04) and tracheostomies (45% vs. 55%, RR 0.82, 95% CI 0.54 to 1.25) in the early surgery group compared to the late surgery group; however, this difference did not reach statistical significance[20,29].

<u>Rationale for Recommendation</u>

The outcomes ranked as critical for decision-making were improvement of AIS by ≥ 2 grades, AMS, and FIM as well as any major complications, mortality, surgical device related complications, sepsis secondary to systemic infection, pressure ulcers, neurological deterioration, cardiopulmonary events and need for tracheostomy. It was also decided that the proposed recommendation would not distinguish between patients with complete and incomplete injuries or specify the level of injury. The reason behind this decision was based on the results of the meta-analysis that assessed whether injury severity or level modified the effects of timing of surgery with respect to improvement by ≥2 grades on the AIS. Based on the results, the risk ratio estimates in both the complete and incomplete injury groups suggested benefit of early decompression. Furthermore, the confidence intervals for the estimates of effect overlapped and the test for interaction was not significant. Based on these results, it can be concluded that injury severity does not modify the treatment effect of early surgery in patients with SCI. Unfortunately, data were insufficient to hypothesize whether the level of SCI alters the effect of early surgery. However, given the

For Peer Review

similarity of point estimates and overlapping confidence intervals when comparing the impact of early surgery on outcomes across injury levels, there is likely no modification of treatment effect. Furthermore, the risk ratios suggest potential benefit of early surgery across all levels of SCI.

An important first step in the guideline development process was to identify the minimum clinically important difference (MCID) for each critical outcome. The MCID refers to the smallest change in a score that a patient would perceive as beneficial or meaningful[34,35]. It is postulated that neurological improvement is only meaningful to a patient living with SCI if it translates to functional benefits and enhancement in quality of life. Furthermore, the MCIDs for neurological recovery and functional outcomes are likely dependent on the level and severity of SCI. The International Standards for Neurological Classification of SCI Patients (ISNCSCI) is the preferred tool to assess the severity and level of SCI and quantify motor and sensory dysfunction[36-38]. It is an ordinal scale that is not linear, meaning that a one-grade change carries different value depending on patient characteristics[39]. Using distribution-based methods, the MCID for the upper extremity motor score (UEMS), lower extremity motor score (LEMS) and total motor score (TMS) was computed as 2.72, 3.66 and 4.48, respectively in a population of acute or subacute traumatic or nontraumatic SCI[40]. For individuals with cervical SCI, the MCID for the UEMS ranged from 1.9 for AIS D patients to 2.91 for ASIA A patients[40]. Similarly, the MCID of the LEMS was lower in patients with AIS D injuries (1.5) compared to individuals with more severe AIS grades (2.45 AIS C, 2.35 AIS B). Finally, the MCID of the TMS was 2.22 in AIS D, 4.16 in AIS C, 3.23 in AIS B and 2.91 in AIS A patients[40]. In other studies, the minimum detectable change of the UEMS was estimated to be two or three[41,42], with six points being the average spontaneous improvement seen in acute cervical sensorimotor complete injuries[39]. Despite extensive efforts to define the MCID for UEMS, LEMS, and TMS in SCI patients, it remains a clinical challenge because the myotomal distribution of improvement may be more important than the absolute change in the motor score. For example, trace flicker (score of 1) in two myotomes on each side of the body is not as clinically meaningful to a patient as obtaining a 4-point improvement across two myotomes in a single extremity. In a study by Scivoletto et al (2013), the effect-size based estimate for a substantial change was also calculated[40]. Based on their results, a 7.45-point change was considered substantial for UEMS, 8.4 for LEMS and 10.65 for TMS. These results were presented to the GDG who were asked to vote on the MCID for AMS using either clinical expertise or personal experiences. Forty-five percent of participants voted that any change in AMS was meaningful for individuals living with SCI, while 18% stated 2 points, 5% selected 3 points, 23% agreed 4 points and 9% chose 5 points as the likely MCID. While there was a lack of consensus, it is important to note that the majority of the GDG (91%) agreed that an improvement in AMS of four points (and sometimes less than this) is likely to be clinically meaningful for patients with

For Peer Review

SCI. While this method for defining what constituted a "clinically meaningful change" may not be grounded in a formal statistical analysis of empirical evidence, it did incorporate the opinions of a diverse set of stakeholders, including individuals living with SCI.

A two-grade improvement in the AIS was considered a critical outcome for decision-making, while a one grade improvement was deemed important but not critical. The GDG agreed that a two-grade improvement is likely to be clinically meaningful in the majority of patients. While a one grade improvement may still lead to some functional improvements in select patients[43], it does not reliably translate to improved functional status, reduced disability or enhanced quality of life. For example, a change in AIS from A to B will likely not significantly impact overall function, nor would improving from ASIA B to C if the only gain is a trace flicker of movement in the lower extremities. In patients with ASIA C or D injuries, however, a one grade change in AIS may translate to significant improvements in function. Given the variable clinical significance of a one grade change in AIS, the GDG agreed that a two-grade improvement should be the main outcome driving the clinical recommendation.

As highlighted above, the overall quality of evidence for these critical outcomes was rated as "moderate," "low" or "very low.' The GDG agreed that the main outcomes driving the recommendation on timing of surgery are improvement in AIS by ≥2 grades and improvement in AMS. In contrast, the GDG acknowledged that outcomes related to harm should not drive the recommendation as early surgery does not increase the risk of major complications or mortality, and because the majority of studies comparing early and late surgery were underpowered to detect any difference in rates of adverse events. The GDG also agreed that neurological outcomes at >6 to 12 months are more relevant than at <6-month follow-up as patients often continue to demonstrate improvement up to and sometimes after a year following injury. Based on these discussions, 95% of the GDG voted that the overall certainty of evidence was moderate.

The GDG agreed that there was either probably no (65%) or no (30%) important uncertainty or variability with respect to how much key stakeholders value the main outcomes. Based on professional opinion, it is likely that clinicians and patients would similarly value the outcomes related to neurological and functional improvement and serious adverse events. While these outcomes would also be valued by payers, this stakeholder group would also value length of stay, resource use and cost-effectiveness.

The anticipated desirable effects of early surgical decompression include clinically meaningful improvements in AIS and AMS. Based on moderate evidence, patients were approximately two times more likely to improve by ≥ 2 AIS grades at 6 months (RR 2.76, 95% CI 1.60 to 4.98) or 12 months

For Peer Review

(1.95, 95% CI 1.26 to 3.18) if they were decompressed within 24 hours of injury compared to after 24 hours. An effect size greater than 2 is considered large. In addition, moderate evidence suggested that early surgery resulted in a 4.50 (95% CI 1.70 to 7.29) point improvement in AMS at > 6 to 12 months.

Ninety percent of the GDG agreed that the anticipated desirable effects of early surgical decompression were either moderate (48%) or large (43%). Participants who voted “moderate” commented that while a two-point improvement in AIS and a 4.5 improvement in AMS are likely clinically meaningful, the MCID of these outcome tools have not been rigorously assessed in a traumatic SCI population. Furthermore, the GDG acknowledged that neurological recovery on the AIS and AMS may inconsistently translate to functional benefit and often depends on the myotomal distribution of improvements (as described above) or whether changes are appreciated in the upper or lower extremities. In contrast, participants who selected “large” emphasized that any improvement in neurological function has the potential to significantly impact the quality of life of an individual living with SCI.

The anticipated undesirable effects of early surgical decompression include any major complication, mortality, surgical device related complications, sepsis secondary to systemic infection, pressure ulcer, neurological deterioration, cardiopulmonary dysfunction and need for tracheostomy. Based on very low to moderate evidence, there was no difference in the rate of complications between patients treated surgically within 24 hours and those treated after 24 hours. However, most studies were underpowered to detect a difference in rates of complications based on timing of surgery. Furthermore, it is often difficult to distinguish complications associated with the underlying SCI from those related to surgery. Fifty-five percent of the GDG voted that the undesirable effects of early surgery were likely trivial and 45% agreed they were small. The anticipated undesirable effects were considered trivial not because these complications are trivial but because there are no additional risks following early compared to late surgical intervention. The GDG unanimously agreed that the balance between the desirable and undesirable effects favor early surgical decompression given the potential neurological benefit without an increased risk of complications.

The financial burden of SCI is significant due to costs associated with acute and chronic medical care, in addition to costs related to loss of productivity. Individuals with SCI have a higher rate of rehospitalizations, visits to primary care physicians and home services compared to a matched cohort of individuals without SCI[44,45]. Based on a systematic review of the literature, the mean cost of SCI management in the first year was $119,870 in Canada and between $300,880 and $634,400 in the United

For Peer Review

States[46]. Furthermore, based on a study by Krueger et al (2013), lifetime expenses range from $2,105,811 to $3,026,028 CAD for individuals with incomplete and complete tetraplegia, respectively[2].

Although the financial burden of SCI is well known, there was limited data on whether costs differ based on timing of surgical intervention. A study by Mac-Thiong et al (2012) compared the cost of acute management between individuals receiving early versus late surgery for SCI[47]. Based on their results, the hospitalization costs were $20,525 ± 13,791 in the <24-hour surgery group and $25,036 ± 17,886 in the ≥24-hour surgery group. There were, however, significant baseline differences confounding these results: patients treated within 24 hours of injury were younger, more likely to have a lower AIS and more likely to have sustained a thoracolumbar neurological injury[47]. A cost utility analysis was conducted by Furlan et al (2016) to compare the cost-effectiveness of early (≤24 hours) versus late (>24 hours) surgery in patients with acute traumatic cervical SCI[48]. In patients with motor complete SCI, early decompression cost $524,483.81 USD/QALY whereas late surgery cost $544,851.71 USD/QALY. Furthermore, the potential savings of early surgery was estimated to be $58,368,024 USD/QALY. However, using a Monte-Carlo simulation, early decompression was more cost-effective 26.3% of the time but less cost-effective 23.4% of the time. These results indicate that no strategy was clearly dominant in motor complete SCI. In a group of patients with motor incomplete SCI, early decompression cost $83,009.19 USD/QALY, whereas late decompression cost $81,233.10 USD/QALY. Early surgery was deemed to be more cost-effective than late surgery with the potential to save $536,217.33 USD/QALY in individuals with motor incomplete SCI. Using Monte-Carlo simulation, early decompression was more cost-effective in 32.6% but less cost-effective in 18.2% of patients with motor incomplete SCI. Again, these findings indicate that no strategy is clearly dominant. This study concluded that early decompression is more cost-effective than late decompression in patients with complete and incomplete SCI even if there was clearly no dominant strategy[48]. The level of evidence was not graded for these results.

Length of stay was also considered when assessing overall cost and resource requirement. Six studies were identified that compared hospital length of stay between patients treated early versus late for acute SCI [32,49] [50] [15,20,21]. In a meta-analysis, pooled estimates across five studies indicated that patients undergoing early surgery had a shorter length of stay than those treated late (mean difference -3.52, 95% CI -4.08 to -2.95) [15,20,21,32,50]. The overall level of evidence for this outcome was low. While a shorter length of stay can drastically reduce costs, this difference likely reflects only minor savings when considering the financial burden of SCI. However, institutions often use length of stay as a metric of performance[51]. Furthermore, a shorter time spent in the hospital reduces the risk of hospital-

For Peer Review

acquired complications (e.g. infection, delirium, falls, pressure ulcers and respiratory complications) which can further prolong length of stay, increase cost and impact outcomes. Fifty-eight percent of the GDG agreed the costs and savings associated with early surgical decompression are negligible given that the resource requirements are similar for early and late surgery. Sixteen percent either selected large (11%) or moderate (5%) savings with early surgical decompression as the neurological improvements associated with early surgery may result in significant lifelong savings. An additional 16% of the group selected moderate costs associated with early surgery, 5% stated the resource requirements vary and 5% voted that they did not know given the absence of strong literature.

The GDG agreed that the certainty of evidence related to resource requirements was very low (64%) or low (18%). They voted that cost-effectiveness probably favors (64%) or favors (27%) early surgical decompression given that the resource requirement is likely similar between early versus late surgery and because early surgery is associated with improved neurological outcomes and a shorter length of stay.

The votes varied with respect to the impact of a recommendation of early surgery on health inequities: 38% uncertain, 48% probably reduced, 5% reduced and 10% don't know. If it is assumed that standardized pathways are implemented in order to ensure direct transfer from the site of injury to a trauma center capable of urgently performing surgical decompression, the GDG agreed that health inequities would probably be reduced. If policy makers funded initiatives to ensure patients with traumatic SCI are triaged appropriately and have better access to early surgical intervention, then there would be less disparity across socioeconomic groups and geographic regions.

The GDG agreed that a recommendation for early surgery would be acceptable to key stakeholders (probably yes: 45%, yes 50%) due to the potential improvement in AIS and AMS with no added risks. The GDG acknowledged that patients with devastating injuries may value small neurological improvements as these may translate to clinically meaningful changes in functional status and quality of life. Furthermore, a recommendation for early surgery would likely be acceptable to payers due to no added costs and the potential for significant long-term savings. The GDG also agreed that a recommendation for early surgery would probably be feasible to implement (probably yes 63%, yes: 26%, varies: 11%) but that this would depend on the clinical setting, geographic region and local guidelines.

Considering these factors, 95% of the GDG voted that the desirable consequences clearly outweigh the undesirable consequences in most settings. This consensus led to the formation of a strong recommendation for early decompression in patients with traumatic SCI (100%).

For Peer Review

The final wording that a recommendation be made for surgical decompression within 24 hours post-injury was subjected to a final vote of all the GDG members and consensus was achieved for its approval (>90%). There was one 'dissenting' concern raised in the final voting that the recommendation should be stronger given the quality of the evidence.

Part 2. Ultra-Early Decompressive Surgery in Adult Patients with Acute Spinal Cord Injury

Population: Adult patients with acute SCI
Key Question 2: Should we recommend ultra-early decompressive surgery for adult patients with acute SCI regardless of injury severity and neurological level?
Statement: A recommendation for ultra-early surgery could not be made on the basis of the current evidence because of the small sample sizes, variable definitions of what constituted ultra-early and the inconsistency of the evidence.

Evidence Summary

A systematic review of the literature was conducted in order to summarize and appraise the available evidence on the effectiveness and safety of ultra-early surgical intervention.

Evidence Related to Benefits

Four studies explored the impact of ultra-early surgery on neurological outcomes using the following cutoffs: (i) ≤ 4 versus 4 to 24 hours; (ii) <5 versus 5 to 24 hours; (iii) <8 versus 8 to 24 hours; and (iv) <12 versus 12 to 24 hours[13,18,52,53]. Using an 8-hour threshold, patients treated ultra-early were 4.55 (95% CI 1.13 to 18.29) times more likely to improve by ≥2 grades on the AIS at 6-months than those treated early (8 to 24 hours)[53]. In contrast, a single study failed to detect a difference in the rate of improvement by ≥2 AIS grades at 6 months between patients treated within 4 hours of injury and those decompressed between 4 and 24 hours (RR 0.50, 95% CI 0.16 to 1.50)[52]. Given the limited evidence, it was not possible to make firm conclusions on the effectiveness of ultra-early surgery (<4 or <8 hours) in improving AIS by ≥2 grades at 6 months. The overall level of evidence for this outcome was very low. Two studies explored whether ultra-early surgery was associated with a ≥2 grade improvement on AIS at 12-months[13,18]. Using a 5-hour threshold, patients treated ultra-early were less likely to improve by ≥2 grades on AIS than those treated early (5 to 24 hours) (RR 0.24, 95%CI 0.07 to 0.85)[18]. Using a 12-

hour threshold, there was no difference in the likelihood of achieving ≥2 AIS grades at 12-moths between the ultra-early (<12 hours) and early (12 to 24 hours) groups (RR 1.09, 95% CI 0.39 to 3.04)[13]. Given the results of these two studies, it was not possible to make definitive conclusions on the effectiveness of ultra-early surgery (<5 or <12 hours) in improving AIS by ≥2 grades by 12 months. The overall level of evidence for this outcome was also very low.

A single study compared the median improvement in AMS at 6-months between patients treated ultra-early (<8 hours) and those treated early (8 to 24 hours)[53]. Based on their results, the median improvement in AMS was 38.5 (95% CI 10.0 to 61.0) in the ultra-early group and 15.0 (95% CI 9.9 to 34.0) in the early surgery group.

Since the completion of the systematic review, an additional study was published investigating outcomes of decompression surgery performed ≤12 hours after SCI versus >12 hours [54]. This study found a trend in favor of ultra-early decompression (≤12 hours) for LEMS change at 12 months, however, several methodological limitations render the results of this study challenging to interpret. Specifically, there were significant imbalances in early and late surgical groups, particularly the baseline ASIA score, the study was underpowered, and there was a lack of reporting of clinically relevant outcome measures including upper extremity motor scores for cervical SCI cases. It is therefore unlikely that results from this study would have changed the conclusions or recommendations.

Evidence Related to Harms

Two prospective cohort studies evaluated mortality rates in patients treated ultra-early (<4 hours or <8 hours) versus early (4 or 8 to 24 hours)[52,53]. In the study that evaluated a 4-hour cut-off, the mortality rate was 0 in both the ultra-early and early surgical groups[52]. In the second study, mortality was also infrequent and did not differ between groups: 2/26 in the ultra-early (<8 hours) and 1/22 in the early (8 to 24 hours, RR 1.69, 95% CI 0.16 to 17.44)[53]. The overall level of evidence was low. A single study evaluated rates of neurological deterioration in patients undergoing surgery within 12 hours of injury compared to 12 to 24 hours[13]. Only three patients experienced neurological deterioration in a cohort of 57: two in the ultra-early and one in the early group. There was no significant difference in the frequency of neurological deterioration based on the timing of surgical decompression (RR 1.56, 95% CI 0.15 to 16.27). The overall level of evidence was very low.

For Peer Review

Rationale for Recommendation

The outcomes ranked as critical for decision making were improvement of AIS by ≥ 2 grades and AMS as well as any major complication, including mortality and neurological deterioration. As highlighted above, the overall quality of evidence for these critical outcomes was rated as very low. Similar to part 1, the GDG agreed that the main outcomes driving the recommendation for ultra-early surgery are improvement in AIS grade ≥2 and improvement in AMS at >6 to 12 months. The GDG acknowledged that there is inconsistency with respect to the impact of ultra-early surgery on these critical outcomes depending on what time threshold was evaluated. Based on these discussions, the GDG voted that the overall certainty of the evidence was either very low (87%) or low (13%).

Similar to part 1, the GDG agreed that there was probably no (44%) or no (31%) important uncertainty or variability with respect to how much key stakeholders value the main outcomes.

The anticipated desirable effects of ultra-early surgical decompression include improvement of AIS by ≥2 grades and improvement of AMS. Based on very low evidence, it was not possible to make firm conclusions on the effectiveness of ultra-early surgery (<4, <5, <8, <12) in improving AIS by ≥2 grades at 6- or 12-months[13,18,52,53]. Given the limitations in the literature, 75% of the GDG stated that they did not know how substantial the desirable anticipated effects were for ultra-early surgery.

The anticipated undesirable effects of ultra-early surgery include mortality and neurological deterioration. Based on very low evidence, there were no differences in rates of mortality or neurological deterioration between patients treated ultra-early (<4, <8 or <12 hours) compared to early (4, 8 or 12 to 24 hours)[13,52,53]. Unfortunately, the majority of studies were underpowered to detect a difference in complications based on timing of surgery. Fifty-three percent of the GDG voted that the undesirable effects of very early surgery were likely trivial, 20% agreed they were small and 27% stated they did not know. Given the desirable effects of ultra-early surgery were unknown and the undesirable effects were either trivial or small, the GDG agreed that the balance between the desirable and undesirable effects was unknown.

Although the management of SCI requires substantial resources, there were no studies that compared costs between patients treated ultra-early and early. The GDG, however, agreed that the resources required for ultra-early surgery were likely similar to the resources required for early surgery. Length of hospital stay was also considered when evaluating overall cost and resource requirement of ultra-early

For Peer Review

surgery. A single study by Jug et al (2015) determined that patients undergoing ultra-early (<8 hours) surgery had a shorter length of hospital stay (38.8 ± 24.0 days) than patients treated early (8 to 24 hours) (48.8 ± 40.3 days); however, this difference was not statistically significant (MD 10.0, 95% CI -30.31 to 10.31 days)[53]. Sixty-nine percent of the GDG agreed that the costs and savings associated with ultra-early surgery were negligible given that the resource requirements are likely similar for ultra-early and early surgery. An additional 23% of participants stated they did not know given the absence of strong literature. Ninety-two percent of the GDG voted that there were no studies that compared the resource requirements between ultra-early and early surgical decompression. Similarly, the cost-effectiveness of ultra-early compared to early surgery was largely unknown given the unknown neurological benefit associated with ultra-early surgery and the uncertainty surrounding the resource requirement. Sixty-percent of the GDG stated that they did not know the cost-effectiveness of ultra-early surgery while 40% voted that it does not favor either the intervention or the comparison.

Fifty-four percent of the GDG stated that they did not know the impact of a recommendation of ultra-early surgery on health inequities and 15% noted that they were uncertain. Furthermore, the GDG was uncertain whether a recommendation for ultra-early surgery would be acceptable to clinicians and patients with traumatic SCI due to unclear neurological benefit (uncertain: 62%, don't know: 15%). Furthermore, it was uncertain whether payers would accept a recommendation of ultra-early surgery due to unclear costs and unknown potential for long-term savings. Finally, recommendations for some, if not all, of the time thresholds would be difficult to implement into clinical practice. In a study by Wilson et al (2016), the mean time from injury to arrival at a definitive care center was 8.1 ± 25.5 hours, while the mean time to surgery was 49.4 ± 65.0 hours[55]. Moreover, only 34.2% reached the operating room within 12 hours. As such, pathways of care for SCI would need to be further streamlined in order to successfully implement a recommendation for ultra-early surgery. In addition, some patients require hemodynamic stabilization prior to undergoing surgery, creating further barriers to adopting a recommendation of ultra-early surgery. The voting with respect to the feasibility of ultra-early surgery was therefore split: 21% no, 21% probably no, 50% uncertain and 7% varies.

Considering these factors, 100% of the GDG voted that the balance between the desirable and undesirable consequences was closely balanced or uncertain. As a result of this uncertainty, the GDG agreed that a recommendation for very early surgery could not be made at this time.

The final wording that a recommendation for ultra-early surgical decompression could not be made with the current body of evidence was subjected to a final vote of all the GDG members and consensus was

For Peer Review

achieved for its approval (>80%). Those who dissented either believed that the evidence did support the benefit of ultra-early decompression, or had concerns about whether ultra-early decompression was feasible to achieve or practical.

Evidence Gaps and Future Recommendations

Through the development of these CPGs, we identified several important knowledge gaps and areas of future research:

1. *The impact of surgical timing across different SCI phenotypes, specifically central cord syndrome (CCS)*. Little of the current evidence base on timing of surgery for SCI has specifically focused on the unique entity of CCS. Based on current evidence for other injury patterns and the few studies examining timing of surgery for CCS, it is reasonable to extrapolate that a similar benefit of early surgery is afforded in this patient population. However, as CCS is the most common pattern of incomplete SCI, with an increasing incidence particularly among the growing elderly population, further research on the impact of surgical timing on neurological recovery in these patients is required. It is recognized that since the completion of the systematic reviews and guidelines process, at least two high quality studies on the timing of surgery for CCS and incomplete cervical SCI have been published[56,57]. Future efforts will need to address this new knowledge to update the current guidelines.
2. *The influence of SCI severity or level of injury on the treatment effect of early surgery.* While there are several studies that incorporate patients with various levels and severities of SCI, estimates have typically been pooled and thus conclusions cannot be made in specific patient subgroups. Future studies should be designed to determine the impact of timing of surgical decompression on neurological outcomes in different patient subgroups in order to better establish personalized treatment approaches and correctly triage patients.
3. *The influence of ultra-early surgery on patient related outcome measures.* While there are a number of studies that have attempted to determine the impact of surgery performed earlier than 24 hours, the lack of consistent time frames in the literature has significantly hindered pooling of the evidence. Establishing consistent timing thresholds to be used in future studies is critical in order to definitively determine whether ultra-early surgery provides additional benefits to patients. Careful consideration must be made in future studies to the timing of the neurological examination during this ultra-early period. This indeed is an important limitation in the interpretation of neurologic benefit after ultra-early surgery. Patients undergoing ultra-early surgery must be neurologically assessed very early

after their injury, when they still may be in spinal shock and when their examination is inherently less reliable at predicting outcome. And so, a person deemed "AIS A" when examined 2 hours post-injury who then has an immediate surgical decompression at 4 hours post-injury is not necessarily comparable to a person deemed "AIS A" when examined 14 hours post-injury who then has surgery at 20 hours post-injury. Accounting for this will be important in future studies that try to establish the true neurologic benefit of ultra-early surgery. Furthermore, studies investigating barriers to early surgical intervention and strategies to address them, particularly in under-resourced settings, are critical in ensuring adherence to established CPGs.

4. *Establishment of what constitutes a clinically meaningful improvement on the outcome measures used to evaluate neurological and functional status.* The development and incorporation of new metrics to assess functional improvement in future studies will aid in establishing clearer benchmarks for clinically meaningful improvements.
5. *The cost-effectiveness of early versus late surgery.* Since publication of the last set of guidelines in 2017, there has been only one additional study investigating the cost-effectiveness of early surgery. Nevertheless, there remains a paucity of evidence in this area which necessitates further research.
6. *The issue of what constitutes an effective decompression is an area of evolving interest.* The role of duroplasty, extent of spinal column decompression (e.g. number of levels of laminectomy), surgical approach and the use of intraoperative ultrasound to validate the effectiveness of decompressive surgery are areas of ongoing investigation that will require high quality prospective comparative effectiveness studies to move the field forward.

<u>Implementation Considerations</u>

Dissemination of this guideline will be accomplished at multiple levels in order to ensure effective implementation into clinical practice:

- Presentation at international spine surgery, critical care, neurology, and anesthesiology conferences.
- Scientific and educational courses.
- Online webinar that engage a broad audience in an interactive format.
- Publication of a focus issue in the Global Spine Journal.
- Submission to the National Guideline Clearinghouse.

<u>Internal Appraisal and External Review</u>

For Peer Review

The leader of the GDG and a methodologist from Aggregate Analytics completed an internal appraisal of the final guideline using the AGREE II checklist. A multidisciplinary group of stakeholders were invited to externally review this guideline document prior to publication. This guideline was also reviewed by several professional societies (see Appendix). The methods paper published elsewhere in this focus issue summarizes additional details of these processes and highlights the conflicts of interest of both internal and external reviews.

Plans for Updating

This guideline with be reviewed by AO Spine, Praxis Spinal Cord Institute and members of the leadership group at three to five years following publication. The guideline will be updated at this time, or earlier, if there are changes in (i) the evidence related to benefits and harms, (ii) outcomes deemed critical for decision-making; (iii) knowledge related to resource requirements and cost-effectiveness; and (iv) available interventions and resources.

For Peer Review

References

1. Witiw CD, Fehlings MG. Acute Spinal Cord Injury. J Spinal Disord Tech 2015; 28: 202-210 DOI: 10.1097/BSD.0000000000000287
2. Krueger H, Noonan VK, Trenaman LM et al. The economic burden of traumatic spinal cord injury in Canada. Chronic Dis Inj Can 2013; 33: 113-122
3. Alizadeh A, Dyck SM, Karimi-Abdolrezaee S. Traumatic Spinal Cord Injury: An Overview of Pathophysiology, Models and Acute Injury Mechanisms. Front Neurol 2019; 10: 282 DOI: 10.3389/fneur.2019.00282
4. Dumont RJ, Okonkwo DO, Verma S et al. Acute spinal cord injury, part I: pathophysiologic mechanisms. Clin Neuropharmacol 2001; 24: 254-264 DOI: 10.1097/00002826-200109000-00002
5. Wilson J, Fehlings M. Emerging Approaches to the surgical management of acute traumatic Spinal Cord Injury. Neurotherapeutics 2011; 8: 197-194
6. Ahuja CS, Nori S, Tetreault L et al. Traumatic Spinal Cord Injury-Repair and Regeneration. Neurosurgery 2017; 80: S9-S22 DOI: 10.1093/neuros/nyw080
7. Guha A, Tator C, Endrenyi L. Decompression of the spinal cord improves recovery after acute experimental spinal cord compression injury. Paraplegia 1987; 25: 324-339
8. Dimar J, Glassman S, Raque G et al. The influence of spinal canal narrowing and timing of decompression on neurologic recovery after spinal cord contusion in a rat model. Spine 1999; 24: 1623-1633
9. Fehlings MG, Perrin RG. The timing of surgical intervention in the treatment of spinal cord injury: a systematic review of recent clinical evidence. Spine (Phila Pa 1976) 2006; 31: S28-35; discussion S36 DOI: 10.1097/01.brs.0000217973.11402.7f
10. Fehlings MG, Tetreault LA, Wilson JR et al. A Clinical Practice Guideline for the Management of Patients With Acute Spinal Cord Injury and Central Cord Syndrome: Recommendations on the Timing (</=24 Hours Versus >24 Hours) of Decompressive Surgery. Global Spine J 2017; 7: 195S-202S DOI: 10.1177/2192568217706367
11. Wilson JR, Tetreault LA, Kwon BK et al. Timing of Decompression in Patients With Acute Spinal Cord Injury: A Systematic Review. Global Spine J 2017; 7: 95S-115S DOI: 10.1177/2192568217701716
12. Badhiwala JH, Wilson JR, Witiw CD et al. The influence of timing of surgical decompression for acute spinal cord injury: a pooled analysis of individual patient data. Lancet Neurol 2021; 20: 117-126 DOI: 10.1016/s1474-4422(20)30406-3
13. Aarabi B, Akhtar-Danesh N, Chryssikos T et al. Efficacy of Ultra-Early (< 12 h), Early (12-24 h), and Late (>24-138.5 h) Surgery with Magnetic Resonance Imaging-Confirmed Decompression in American Spinal Injury Association Impairment Scale Grades A, B, and C Cervical Spinal Cord Injury. Journal of Neurotrauma 2020; 37: 448-457 DOI: 10.1089/neu.2019.6606
14. Aarabi B, Akhtar-Danesh N, Simard JM et al. Efficacy of early (≤ 24 Hours), Late (25-72 Hours), and Delayed (>72 Hours) surgery with magnetic resonance imaging-confirmed decompression in american spinal injury association impairment scale grades C and D acute traumatic central cord syndrome caused by spinal stenosis. Journal of Neurotrauma 2021; 38: 2073-2083 DOI: 10.1089/neu.2021.0040
15. Du JP, Fan Y, Liu JJ et al. Decompression for Traumatic Thoracic/Thoracolumbar Incomplete Spinal Cord Injury: Application of AO Spine Injury Classification System to Identify the Timing of Operation. World Neurosurgery 2018; 116: e867-e873 DOI: 10.1016/j.wneu.2018.05.118
16. Haghnegahdar A, Behjat R, Saadat S et al. A Randomized Controlled Trial of Early versus Late Surgical Decompression for Thoracic and Thoracolumbar Spinal Cord Injury in 73 Patients. Neurotrauma Rep 2020; 1: 78-87 DOI: 10.1089/neur.2020.0027
17. Lee S, Kim C, Ha JK et al. Comparison of Early Surgical Treatment With Conservative Treatment of Incomplete Cervical Spinal Cord Injury Without Major Fracture or Dislocation in

For Peer Review

Patients With Pre-existing Cervical Spinal Stenosis. Clin Spine Surg 2021; 34: E141-e146 DOI: 10.1097/bsd.0000000000001065
18. Mattiassich G, Gollwitzer M, Gaderer F et al. Functional Outcomes in Individuals Undergoing Very Early (< 5 h) and Early (5-24 h) Surgical Decompression in Traumatic Cervical Spinal Cord Injury: Analysis of Neurological Improvement from the Austrian Spinal Cord Injury Study. Journal of Neurotrauma 2017; 34: 3362-3371 DOI: 10.1089/neu.2017.5132
19. Qadir I, Riew KD, Alam SR et al. Timing of Surgery in Thoracolumbar Spine Injury: Impact on Neurological Outcome. Global Spine Journal 2020; 10: 826-831 DOI: 10.1177/2192568219876258
20. Sewell MD, Vachhani K, Alrawi A et al. Results of Early and Late Surgical Decompression and Stabilization for Acute Traumatic Cervical Spinal Cord Injury in Patients with Concomitant Chest Injuries. World Neurosurg 2018; 118: e161-e165 DOI: 10.1016/j.wneu.2018.06.146
21. Ter Wengel PV, de Gendt EEA, Martin E et al. Impact of Surgical Timing on Motor Level Lowering in Motor Complete Traumatic Spinal Cord Injury Patients. J Neurotrauma 2022: DOI: 10.1089/neu.2021.0428
22. Shiffman RN, Shekelle P, Overhage JM et al. Standardized reporting of clinical practice guidelines: a proposal from the Conference on Guideline Standardization. Ann Intern Med 2003; 139: 493-498 DOI: 10.7326/0003-4819-139-6-200309160-00013
23. Vernooij RW, Alonso-Coello P, Brouwers M et al. Reporting Items for Updated Clinical Guidelines: Checklist for the Reporting of Updated Guidelines (CheckUp). PLoS Med 2017; 14: e1002207 DOI: 10.1371/journal.pmed.1002207
24. Balshem H, Helfand M, Schunemann HJ et al. GRADE guidelines: 3. Rating the quality of evidence. J Clin Epidemiol 2011; 64: 401-406
25. Li SA, Alexander PE, Reljic T et al. Evidence to Decision framework provides a structured "roadmap" for making GRADE guidelines recommendations. J Clin Epidemiol 2018; 104: 103-112 DOI: 10.1016/j.jclinepi.2018.09.007
26. Alonso-Coello P, Oxman AD, Moberg J et al. GRADE Evidence to Decision (EtD) frameworks: a systematic and transparent approach to making well informed healthcare choices. 2: Clinical practice guidelines. BMJ 2016; 353: i2089 DOI: 10.1136/bmj.i2089
27. Alonso-Coello P, Schunemann HJ, Moberg J et al. GRADE Evidence to Decision (EtD) frameworks: a systematic and transparent approach to making well informed healthcare choices. 1: Introduction. BMJ 2016; 353: i2016 DOI: 10.1136/bmj.i2016
28. Brouwers MC, Kho ME, Browman GP et al. Development of the AGREE II, part 2: assessment of validity of items and tools to support application. CMAJ 2010; 182: E472-478 DOI: 10.1503/cmaj.091716
29. Fehlings MG, Vaccaro A, Wilson JR et al. Early versus delayed decompression for traumatic cervical spinal cord injury: results of the Surgical Timing in Acute Spinal Cord Injury Study (STASCIS). PLoS One 2012; 7: e32037 DOI: 10.1371/journal.pone.0032037
30. Umerani MS, Abbas A, Sharif S. Clinical Outcome in Patients with Early versus Delayed Decompression in Cervical Spine Trauma. Asian spine journal 2014; 8: 427-434 DOI: 10.4184/asj.2014.8.4.427
31. Lenehan B, Fisher CG, Vaccaro A et al. The urgency of surgical decompression in acute central cord injuries with spondylosis and without instability. Spine (Phila Pa 1976) 2010; 35: S180-186 DOI: 10.1097/BRS.0b013e3181f32a44
32. Wilson JR, Singh A, Craven C et al. Early versus late surgery for traumatic spinal cord injury: the results of a prospective Canadian cohort study. Spinal Cord 2012; 50: 840-843 DOI: 10.1038/sc.2012.59
33. Bourassa-Moreau É, Mac-Thiong JM, Ehrmann Feldman D et al. Complications in acute phase hospitalization of traumatic spinal cord injury: does surgical timing matter? J Trauma Acute Care Surg 2013; 74: 849-854 DOI: 10.1097/TA.0b013e31827e1381

For Peer Review

34. Copay AG, Subach BR, Glassman SD et al. Understanding the minimum clinically important difference: a review of concepts and methods. Spine J 2007; 7: 541-546 DOI: 10.1016/j.spinee.2007.01.008
35. Jaeschke R, Singer J, Guyatt GH. Measurement of health status. Ascertaining the minimal clinically important difference. Control Clin Trials 1989; 10: 407-415 DOI: 10.1016/0197-2456(89)90005-6
36. Kirshblum S, Waring W, 3rd. Updates for the International Standards for Neurological Classification of Spinal Cord Injury. Phys Med Rehabil Clin N Am 2014; 25: 505-517, vii DOI: 10.1016/j.pmr.2014.04.001
37. Kirshblum SC, Burns SP, Biering-Sorensen F et al. International standards for neurological classification of spinal cord injury (revised 2011). J Spinal Cord Med 2011; 34: 535-546 DOI: 10.1179/204577211X13207446293695
38. Rupp R, Biering-Sorensen F, Burns SP et al. International Standards for Neurological Classification of Spinal Cord Injury: Revised 2019. Top Spinal Cord Inj Rehabil 2021; 27: 1-22 DOI: 10.46292/sci2702-1
39. Wu X, Liu J, Tanadini LG et al. Challenges for defining minimal clinically important difference (MCID) after spinal cord injury. Spinal Cord 2015; 53: 84-91 DOI: 10.1038/sc.2014.232
40. Scivoletto G, Tamburella F, Laurenza L et al. Distribution-based estimates of clinically significant changes in the International Standards for Neurological Classification of Spinal Cord Injury motor and sensory scores. Eur J Phys Rehabil Med 2013; 49: 373-384
41. Marino RJ, Jones L, Kirshblum S et al. Reliability and repeatability of the motor and sensory examination of the international standards for neurological classification of spinal cord injury. J Spinal Cord Med 2008; 31: 166-170 DOI: 10.1080/10790268.2008.11760707
42. Kramer JL, Lammertse DP, Schubert M et al. Relationship between motor recovery and independence after sensorimotor-complete cervical spinal cord injury. Neurorehabil Neural Repair 2012; 26: 1064-1071 DOI: 10.1177/1545968312447306
43. Kirshblum S, Botticello A, Lammertse DP et al. The impact of sacral sensory sparing in motor complete spinal cord injury. Arch Phys Med Rehabil 2011; 92: 376-383 DOI: 10.1016/j.apmr.2010.07.242
44. Skelton F, Hoffman JM, Reyes M et al. Examining health-care utilization in the first year following spinal cord injury. J Spinal Cord Med 2015; 38: 690-695 DOI: 10.1179/2045772314Y.0000000269
45. Dryden DM, Saunders LD, Rowe BH et al. Utilization of health services following spinal cord injury: a 6-year follow-up study. Spinal Cord 2004; 42: 513-525 DOI: 10.1038/sj.sc.3101629
46. Malekzadeh H, Golpayegani M, Ghodsi Z et al. Direct Cost of Illness for Spinal Cord Injury: A Systematic Review. Global Spine J 2022; 12: 1267-1281 DOI: 10.1177/21925682211031190
47. Mac-Thiong JM, Feldman DE, Thompson C et al. Does timing of surgery affect hospitalization costs and length of stay for acute care following a traumatic spinal cord injury? J Neurotrauma 2012; 29: 2816-2822 DOI: 10.1089/neu.2012.2503
48. Furlan JC, Craven BC, Massicotte EM et al. Early Versus Delayed Surgical Decompression of Spinal Cord after Traumatic Cervical Spinal Cord Injury: A Cost-Utility Analysis. World Neurosurg 2016; 88: 166-174 DOI: 10.1016/j.wneu.2015.12.072
49. Dvorak MF, Noonan VK, Fallah N et al. The influence of time from injury to surgery on motor recovery and length of hospital stay in acute traumatic spinal cord injury: an observational Canadian cohort study. J Neurotrauma 2015; 32: 645-654 DOI: 10.1089/neu.2014.3632
50. Rahimi-Movaghar V, Niakan A, Haghnegahdar A et al. Early versus late surgical decompression for traumatic thoracic/thoracolumbar (T1-L1) spinal cord injured patients. Primary results of a randomized controlled trial at one year follow-up. Neurosciences (Riyadh) 2014; 19: 183-191
51. Brasel KJ, Lim HJ, Nirula R et al. Length of stay: an appropriate quality measure? Arch Surg 2007; 142: 461-465; discussion 465-466 DOI: 10.1001/archsurg.142.5.461

For Peer Review

52. Biglari B, Child C, Yildirim TM et al. Does surgical treatment within 4 hours after trauma have an influence on neurological remission in patients with acute spinal cord injury? Ther Clin Risk Manag 2016; 12: 1339-1346 DOI: 10.2147/tcrm.S108856
53. Jug M, Kejžar N, Vesel M et al. Neurological Recovery after Traumatic Cervical Spinal Cord Injury Is Superior if Surgical Decompression and Instrumented Fusion Are Performed within 8 Hours versus 8 to 24 Hours after Injury: A Single Center Experience. J Neurotrauma 2015; 32: 1385-1392
54. Hosman AJF, Barbagallo G, van Middendorp JJ. Neurological recovery after early versus delayed surgical decompression for acute traumatic spinal cord injury. Bone Joint J 2023; 105-b: 400-411 DOI: 10.1302/0301-620x.105b4.Bjj-2022-0947.R2
55. Wilson JR, Voth J, Singh A et al. Defining the Pathway to Definitive Care and Surgical Decompression after Traumatic Spinal Cord Injury: Results of a Canadian Population-Based Cohort Study. J Neurotrauma 2016; 33: 963-971 DOI: 10.1089/neu.2015.4258
56. Badhiwala JH, Wilson JR, Harrop JS et al. Early vs Late Surgical Decompression for Central Cord Syndrome. JAMA Surg 2022: DOI: 10.1001/jamasurg.2022.4454
57. investigators O, Chikuda H, Koyama Y et al. Effect of Early vs Delayed Surgical Treatment on Motor Recovery in Incomplete Cervical Spinal Cord Injury With Preexisting Cervical Stenosis: A Randomized Clinical Trial. JAMA Netw Open 2021; 4: e2133604 DOI: 10.1001/jamanetworkopen.2021.33604

For Peer Review
