# Supplementary material for: The 2023 AO Spine-Praxis Guidelines in Acute Spinal Cord Injury: What Have We Learned? What Are the Critical Knowledge Gaps and Barriers to Implementation?
Source: Global Spine J. 2024 Mar 25;14(3 Suppl):223S–230S. doi: 10.1177/21925682231196825 (PMC10964887; doi:10.1177/21925682231196825)
Supplement: Supplemental Material - The 2023 AO Spine-Praxis Guidelines in Acute Spinal Cord Injury: What Have We Learned? What Are the Critical Knowledge Gaps and Barriers to Implementation? [file sj-pdf-3-gsj-10.1177_21925682231196825.pdf]

An Overview of the Methodology Used to Develop Clinical Practice Guidelines for the Management of Acute and Intraoperative Spinal Cord Injury

Journal:	*Global Spine Journal*
Manuscript ID	Draft
Manuscript Type:	Special Issue- Knowledge Forum SCI Guidelines
Keywords:	spinal cord injury, trauma, decompression

Abstract:

Study Design
An overview of the methods used to develop clinical practice guidelines (CPGs).

Objectives
CPGs help to translate the current evidence into actionable recommendations, standardize care across centers, optimize patient outcomes, and reduce costs and unnecessary interventions. Furthermore, they can be used by patients to assist in making decisions about certain treatments and by policy makers to inform allocation of resources. The objective of this article is to summarize the methods used to develop CPG for the timing of surgery and hemodynamic management of acute spinal cord injury (SCI) and the identification and treatment of intraoperative SCI (ISCI).

Methods
The CPGs were developed using standards established by the Institute of Medicine (now the National Academy of Medicine), the Guideline International Network and several other organizations. Systematic reviews were conducted according to accepted methodological standards (e.g., Institute of Medicine, Agency for Healthcare Research and Quality and Patient-Centered Outcomes Research Institute) in order to summarize the current body of evidence and inform the guideline development process. Protocols for each guideline were created. A multidisciplinary guideline development group (GDG) was formed that included individuals living with SCI as well as clinicians from the broad range of specialties that encounter patients with SCI: spine or trauma surgeons, critical care physicians, rehabilitation specialists, neurologists, anesthesiologists and other healthcare professionals. Individuals living with SCI were also included in the GDG. The Grading of Recommendations, Assessment, Development and Evaluations (GRADE) approach was used to rate the certainty of the evidence for each critical outcome...

SCHOLARONE™
Manuscripts

Abstract

Study Design

An overview of the methods used to develop clinical practice guidelines (CPGs).

Objectives

CPGs help to translate the current evidence into actionable recommendations, standardize care across centers, optimize patient outcomes, and reduce costs and unnecessary interventions. Furthermore, they can be used by patients to assist in making decisions about certain treatments and by policy makers to inform allocation of resources. The objective of this article is to summarize the methods used to develop CPG for the timing of surgery and hemodynamic management of acute spinal cord injury (SCI) and the identification and treatment of intraoperative SCI (ISCI).

Methods

The CPGs were developed using standards established by the Institute of Medicine (now the National Academy of Medicine), the Guideline International Network and several other organizations. Systematic reviews were conducted according to accepted methodological standards (e.g., Institute of Medicine, Agency for Healthcare Research and Quality and Patient-Centered Outcomes Research Institute) in order to summarize the current body of evidence and inform the guideline development process. Protocols for each guideline were created. A multidisciplinary guideline development group (GDG) was formed that included individuals living with SCI as well as clinicians from the broad range of specialties that encounter patients with SCI: spine or trauma surgeons, critical care physicians, rehabilitation specialists, neurologists, anesthesiologists and other healthcare professionals. Individuals living with SCI were also included in the GDG. The Grading of Recommendations, Assessment, Development and Evaluations (GRADE) approach was used to rate the certainty of the evidence for each critical outcome. The "evidence to recommendation" framework was then used to translate the evidence obtained from the systematic review to an actionable recommendation. This framework provides structure when assessing the body of evidence and considers several additional factors when rating the strength of the recommendation, including the magnitude of benefits and harms, patient preferences, resource use, health equities, acceptability and feasibility. Finally, the CPGs were appraised both internally and externally.

Results

NA

For Peer Review

Conclusions

Development of these CPGs followed the methodology proposed by the Institute of Medicine the Guideline International Network and the GRADE Working Group. It is anticipated that these CPGs will assist clinicians implement the best evidence into practice and facilitate shared-decision making with patients.

For Peer Review

Introduction

The Institute of Medicine (IOM, now the National Academy of Medicine) defines clinical practice guidelines (CPGs) as "statements intended to optimize patient care that are informed by a systematic review of the literature and an assessment of the benefits and harms of alternative care options.[1]" CPGs help to translate the current evidence into actionable recommendations, standardize care across centers, optimize patient outcomes, and reduce costs and unnecessary interventions[2]. Furthermore, they can be used by patients to assist in decision making and by policy makers to inform allocation of healthcare resources. In contrast, CPGs should not encourage "cookbook" medicine, trump clinical judgement or be used for reimbursement policies, performance measures, or legal precedents.

The IOM, the Guideline International Network and several other international organizations have proposed standards for guideline development in order to ensure that recommendations are reliable and implementable[1,3]. Important principles for generating CPGs are that they must:

- Be based on a methodologically sound systematic review of the literature that synthesizes the best available evidence[1,3].
- Be developed by a panel that includes representation from key stakeholder groups affected by the recommendations (I.e. a multidisciplinary group of clinicians, individuals with lived experience and their caregivers and policy makers).
- Consider the values and preferences of providers, patients and policy makers.
- Include a rating of both the quality of evidence and the strength of the recommendation.
- Provide a clear explanation of the balance between the benefits and risks, alternative care options and resource use.
- Outline implementation strategies that consider personal, guideline-related and external factors that may impede knowledge translation.
- Be critically appraised by both internal and external reviewers.
- Be updated when new evidence arises related to benefits or harms of proposed or alternative interventions.

Furthermore, the process for developing a CPG must be transparent and publicly accessible and minimize intellectual and financial conflicts of interest.

For Peer Review

Guideline developers have increasingly adopted the Grading of Recommendations, Assessment, Development and Evaluations (GRADE) approach to rate the certainty of the evidence and the strength of the recommendations. This system provides structure when assessing the body of evidence and considers several additional factors when rating the strength of the recommendation, including the magnitude of benefits and harms, patient preferences, resource use, health equities, acceptability and feasibility. This article highlights the methodology used to develop CPGs on the timing of surgical decompression and hemodynamic management of spinal cord injury (SCI) and the identification and management of intraoperative SCI (ISCI).

Overview of the Guideline Development Process

Figure 1 highlights the four steps involved in developing and disseminating CPGs: (i) identify critical knowledge gaps and define the clinical problem; (ii) conduct systematic reviews of the literature to synthesize the available evidence and assess the risk of bias; (iii) translate the evidence into recommendations using the GRADE framework; and (iv) implement the recommendations into clinical practice by identifying and addressing important barriers.

Identify Critical Knowledge Gaps and Define the Clinical Problem

This CPG aimed to develop recommendations on (i) the timing of surgical decompression in acute SCI; (ii) the hemodynamic management of acute SCI; and (iii) the identification and management of ISCI in patients undergoing surgery for spine-related pathology. The leadership group considered these topics to be high-priority as recommendations have the potential to improve healthcare outcomes, enhance quality of life and reduce mortality in individuals with acute or intraoperative SCI. Furthermore, SCI in general is a condition with significant disease burden, cost of management and variability in practice standards. Finally, for the two CPGs being updated, the leadership group agreed that there was new evidence available that may impact current recommendations and help resolve controversy or uncertainty around these topics.

The key questions addressed by the CPG included:

Timing of Surgical Decompression:

For Peer Review

- Should we recommend early decompressive surgery (≤24 hours after injury) for adult patients with acute SCI regardless of injury severity and neurological level?
- Should we recommend ultra-early decompressive surgery for adult patients with acute SCI regardless of injury severity and neurological level?

Hemodynamic Management:

- Should we recommend the augmentation of MAP to at least 75-80mmHg and not higher than 90-95mmHg in order to optimize spinal cord perfusion in acute SCI?
- Should we recommend the augmentation of MAP for a duration of 3-7 days in order to optimize spinal cord perfusion in acute SCI?
- Should we recommend the use of a specific vasopressor in order to achieve MAP-directed goals in patients with acute SCI?

Intraoperative Spinal Cord Injury:

- Should we recommend intraoperative neurophysiologic monitoring for patients undergoing spine surgery deemed to be "high risk"?
- Should we recommend that patients at "high risk" for ISCI during spine surgery be proactively identified, that after identification of such patients, multi-disciplinary team discussions be undertaken to manage patients, and that an intraoperative protocol including the use of intraoperative neuromonitoring (IONM) be implemented?

The Systematic Review Team and Guideline Development Group

The leadership group consisted of two chairs, a leader of the guideline development group (GDG) and a leader of the systematic review team. The responsibilities of this group are summarized in Table 1.

The systematic review team was multidisciplinary and consisted of spine surgeons, neurologists, critical care physicians and rehabilitation medicine specialists. Members of the systematic review team were responsible for providing clinical input and expertise and drafting the introduction and discussion sections of each manuscript. An independent organization specializing in systematic and comparative effectiveness reviews (Aggregate Analytics, Inc.) guided the process and assisted in rating the strength of evidence. There was some overlap between the systematic review team and the GDG.

For Peer Review

The GDG included clinicians from the broad range of specialties that encounter patients with SCI: spine or trauma surgeons, critical care physicians, rehabilitation specialists, neurologists, anesthesiologists and other healthcare professionals (Table 2). Patient advocates and individuals living with SCI were also represented in the GDG. Members of the GDG had full editorial independence from the two sponsors and were required to disclose any intellectual or financial conflicts of interest. It was recognized that key opinion leaders and individuals who conduct research in these fields of SCI likely have their own perspectives. It was required that members of the GDG were open to putting these opinions aside, engaging in robust discussions and embracing the perspectives of others in order to formulate consensus-based recommendations. Participants of the GDG were responsible for developing the guideline protocol, participating in online meetings, reviewing the systematic reviews of the literature, creating evidence-based recommendations using the GRADE approach, and creating the guideline document. Aggregate Analytics, Inc provided methodological support for guideline development.

Methodologists from Aggregate Analytics Inc. were non-voting members of the GDG and provided expertise in conducting the systematic reviews of the literature and applying GRADE methodology to formulate recommendations.

Developer

This guideline was developed under the auspices of AO Spine and Praxis Spinal Cord Institute. These funding bodies did not control or influence the editorial content of the articles or the guidelines process and were completely independent from the GDG.

AO Spine is an academic professional society and a Clinical Division of the AO Foundation based in Davos Switzerland. In its vision statement, AO Spine describes itself as a “leading global academic community for innovative education and research in spine care, inspiring lifelong learning and improving patients' lives.” Further, it as “an international community of spine surgeons generating, distributing, and exchanging knowledge to advance science and the spine care profession through research, education, and community development.” The funding from AO Spine comes from intramural and extramural sources. The intramural funding consists of core and additional funding, both of which come from the AO Foundation. The extramural funding comes from various external sources. Praxis Spinal Cord Institute is a Canadian-based not-for-profit organization that aims to accelerate the translation of scientific discoveries into improved treatments for individuals with SCI. In its mission statement, Praxis wishes to

For Peer Review

"lead collaboration across the global SCI community by providing resources, infrastructure and knowledge." It is also the purpose of this organization to "accelerate the translation of evidence and best practices to reduce the incidence and severity of SCI, reduce long-term costs, and enhance the quality of life for those living with SCI." The funding from Praxis Spinal Cord Institute was from Western Economic Diversification Canada.

Systematic Review of the Literature

Systematic reviews were conducted according to accepted methodological standards (e.g. Institute of Medicine, Agency for Healthcare Research and Quality and Patient-Centered Outcomes Research Institute) in order to summarize the current body of evidence and inform the guideline development process. Methodologists from Aggregate Analytics Inc. worked with clinical experts from the systematic review team to ensure that the reviews were methodologically rigorous, clinically accurate, appropriate and relevant. Detailed methods were described in the individual reviews, including information on search strategy, inclusion and exclusion criteria, data extraction and evaluation of risk of bias. The electronic databases that were searched included MEDLINE, ClinicalTrials.gov, EMBASE and *The Cochrane Library.* Reference lists of included articles and previous systematic reviews were also searched. Protocols for each systematic review were published on PROSPERO (CRD42021292229: Interventions to Optimize Spinal Cord Perfusion in Patients with Acute Traumatic Spinal Cord Injuries: Systematic Review Update; CRD42021292237 Timing of Decompression in Patients with Acute Spinal Cord Injury: Systematic Review Update; CRD42022298841 Management of and risk factors for intraoperative spinal cord injury occurring as the result of spine surgery: Systematic review; CRD42023384158 Neuromonitoring for Detecting Intraoperative Spinal Cord Injury During Spinal Surgery: A systematic review and meta-analysis of diagnostic test accuracy studies). A scoping review was conducted to address the contextual questions pertaining to ISCI that could not be answered by a formal review, including the definition, frequency and management of ISCI (CRD4202229884). Methods provided by the U.S. Preventative Services Task Force for answering conceptual questions were used to guide the scoping review[4]. Table 3 summarizes the key questions for each systematic and scoping review.

For individual studies, risk of bias was assessed using pre-defined criteria: the Cochrane tool for risk of bias for randomized controlled trials[5-7], the Risk of Bias In Non-randomized Studies of Interventions (ROBINS-I) for observational studies[8] the National Institutes of Health quality assessment tool for non-comparative studies[9], the Quality in Prognosis Studies (QUIPS) for risk factor studies and the Quality Assessment of Diagnostic Accuracy Studies (QUADAS-2) for diagnostic studies[10,11]. Previous meta-

For Peer Review

analyses, pooled analyses or published systematic reviews were assessed using AMSTAR-2 criteria and/or guidance related to reporting of specialized analyses[12-16].

The strength of the evidence across studies for primary outcomes was determined using GRADE as described in the AHRQ Methods Guide[17]. Guidance provided by the GRADE Working Group was also used when synthesizing evidence on risk factors[18,19]. This process was used to determine how confident the GDG could be about the estimate of effects[20,21]. For the updated reviews, the strength of evidence was assessed across the *totality* of evidence available (i.e., across studies included in the original review as well as newly identified studies). The initial rating of the quality of evidence was determined by whether the studies were randomized controlled trials (baseline level = HIGH) or observational studies (baseline level = LOW)[21]. The quality of evidence was then upgraded or downgraded based on a number of factors. Criteria for downgrading the quality by 1 or 2 levels included limitations in the study design that introduce a risk of bias, inconsistency of results, indirectness of evidence, imprecision and publication/reporting bias[22-26]. Alternatively, reasons for upgrading the quality by 1 or 2 levels included a large magnitude of effect, if plausible confounding would reduce the demonstrated effect or increase the effect if no effect was observed, or if there was a dose-response gradient [27]. The strength of evidence was only upgraded if it was not downgraded in any of the five primary domains. Table 4 summarizes how to determine whether the quality of evidence should be upgraded or downgraded. Following this process, the quality of evidence is rated as high, moderate, low or very low [21]. Table 5 highlights how to interpret the rating of the quality of evidence.

Results from these systematic, scoping and narrative reviews as well as evidence tables were distributed to the GDG and were presented at an online meeting. The systematic review team leader was also a member of the GDG and participated in discussions in order to ensure understanding of the evidence and appropriate interpretation of the effect size for each outcome.

Updating and Developing Clinical Practice Guidelines Using the GRADE Approach

A guideline protocol was formulated using the Conference on Guidelines Standardization (COGS) checklist[28,29]. This checklist was created by a multidisciplinary group of individuals with considerable experience in guideline development, dissemination and implementation in order to standardize guideline reporting. The protocol summarizes the focus, rationale and objectives of the CPGs, defines important terms, and highlights the aspects of care covered by the CPG, the proposed users and settings and implementation strategies. The Checklist for Reporting the Updating Process (CheckUP) was also used to

For Peer Review

guide the update of the CPG on timing of surgical decompression and hemodynamic management of SCI[30]. The guideline protocol *A Clinical Practice Guideline on the Timing of Surgical Decompression and Hemodynamic Management of Acute Spinal Cord Injury and the Identification and Treatment of Intraoperative Spinal Cord Injury: Introduction, Rationale and Scope."*

The leadership group was responsible for ranking the outcomes reported in the systematic review based on importance in determining treatment options and influencing decision-making: critically important, important but not critical and of limited importance. The ranking was reviewed during the GDG meetings and any changes were voted on by the GDG. Each guideline document summarizes the outcomes that were deemed to be critically important when developing the recommendations.

The GRADE "evidence-to-recommendation" framework was used to support the guideline development process [31-33]. This tool is used to grade the strength of each recommendation by considering the overall certainty of the evidence for benefits and harms as well as available information for other factors, including patient values, resource use and cost-effectiveness, impact on health inequities, and the acceptability and feasibility of various treatment options. This framework ensures that discussions among participants are structured, disagreements are identified and that the recommendations consider are informed by the best available evidence. Where evidence was not available or sparse, members of GDG were asked to provide their expert opinions or personal experiences. Furthermore, this model can help the target audience determine how judgements were made by the panel and whether the recommendations should be adopted in specific settings. Ultimately, the GRADE criteria have been applied in numerous guidelines in order to increase transparency, ensure rigor of development and emphasize the importance of integrating the opinions of all stakeholders affected by the recommendations. Table 6 summarize the questions included in the "evidence-to-recommendation" framework as well as the response options. The GRADE handbook was referred to throughout the process when there were questions on how to interpret components of the framework[6]. After answering each question summarized in Table 6, the next step was to evaluate the balance between the desirable and undesirable consequences and determine the strength and direction of each recommendation. The four primary factors that influenced the strength of the recommendation are the balance between desirable and undesirable outcomes, the confidence in the magnitude of the estimate of effect, the values and preferences of key stakeholders, and resource use [34,35].

Three separate online meetings were held over Zoom in order to translate the evidence summarized in the systematic reviews into actionable recommendations. In preparation for the guideline development meeting, the leader of the GDG created "strawman" recommendations using the GRADE framework in

For Peer Review

order to facilitate initial discussions. This document was shared ahead of the meeting and reviewed during the meeting. Members of the GDG anonymously voted on each question in the framework. If there were discrepancies in voting, participants were asked to justify their response by sharing their perspectives, clinical expertise or personal experiences. A threshold of 80% was considered consensus. Results of the voting and discussions were documented throughout the process and are summarized in the "rationale for recommendation" section of each guideline. The final recommendations were created by the leadership group and distributed to the GDG via Redcap for voting. The wording of each recommendation was refined based on the feedback submitted by the GDG.

Interpretation of the Recommendations

GRADE has delineated four types of recommendations based on the confidence in the desirable and undesirable consequences. If the GDG was confident (based on overall strength of evidence for benefits and harms) that the desirable effects outweighed the undesirable effects or vice versa, a strong recommendation was generated either for or against a particular intervention. In contrast, if the GDG was less confident about the balance between the desirable and undesirable consequences, a weak recommendation was proposed. For the purpose of this CPG, the strength of the recommendation was reflected in its wording. For example, "we recommend" denotes that the recommendation is strong, while "we suggest" indicates that the recommendation is weak[35-37]. In situations where the evidence is insufficient or unavailable, expert consensus was required to formulate the recommendations.

The strength of the recommendation has different implications for patients, clinicians and policy makers. A strong recommendation indicates that most patients would want to and should receive the recommended course of action and that the recommendation can be adapted as policy in most situations [34,37]. Furthermore, formal decision aids are unlikely to be needed to assist patients in making a decision consistent with their values and preferences[34]. A weak recommendation reflects that (i) the majority of individuals would want the suggested course of action, but many would not, (ii) clinicians must recognize that different choices will be appropriate for different patients and should help a patient arrive at a decision consistent with his or her values or preferences, and (iii) policy making will require substantial debate and involvement of many stakeholders [34,37].

Internal Appraisal

For Peer Review

The Appraisal of Guidelines for Research and Evaluation (AGREE) II is an instrument designed to evaluate the quality of CPGs and provide a framework for development[38]. This tool can be used by healthcare providers, policy makers, administrators, professional organizations and patients in order to assess the validity of the CPG and evaluate whether the recommendations should be implemented into clinical practice or inform changes in policy. The AGREE II consists of 23 items (1= strongly disagree, 7=strongly agree) organized into the following domains: scope and purpose, stakeholder involvement, rigour of development, clarity of presentation, applicability and editorial independence [39]. This tool also asks the appraiser to rate the overall quality of the guideline and determine whether it should be used in clinical practice. Table 7 summarizes the key questions included in AGREE II. The leader of the GDG along with the methodologists used the AGREE II tool to internally review and appraise each CPG.

The CheckUP tool also facilitated internal review of the updated guidelines[30]. Given the overlap of items with AGREE-II, only the items specific to distinguishing the updated and previous versions were used to inform final editing. Using these tools, modifications were made to the guideline documents and approved by the GDG.

External Review

A multidisciplinary group of clinicians were invited to externally review the guideline document. These individuals were selected based on their clinical expertise and their willingness to participate. Each reviewer was required to disclose any relevant financial or intellectual conflicts of interest. The CPGs were also reviewed by prominent societies in the fields of spine surgery and critical care. Comments and feedback from these external reviewers were assessed by the GDG leadership and incorporated into the final draft. Substantial changes in the recommendations were subjected to approval by the GDG. The final CPGs were distributed to the AO Spine and Praxis Spinal Cord Institute for their endorsement.

Update Plan

The guidelines will be reviewed by the primary sponsor at three to five years following publication. A working group will monitor the body of literature and search for new evidence that may influence the proposed recommendations. The working group will discuss the need to update the guideline with the leadership of the sponsoring organization. An update to the CPG is recommended if there are changes in (i) the evidence related to harms and benefits; (ii) outcomes which would be considered important for

For Peer Review

decision-making; (iii) ranking of current critical and important outcomes; and (iv) available interventions and resources.[40]

Figure Legends

Figure 1. An Overview of the Guideline Development Process
Extracted from a previously published article in the Global Spine Journal by Tetreault et al (2019) entitled: Development and Implementation of Clinical Practice Guidelines: An Update and Synthesis of the Literature with a Focus in Application to Spinal Conditions.

For Peer Review

References

1. Institute of Medicine (U.S.). Committee on Standards for Developing Trustworthy Clinical Practice Guidelines., Graham R. *Clinical practice guidelines we can trust.* Washington, DC: National Academies Press; 2011.
2. Woolf SH, Grol R, Hutchinson A, Eccles M, Grimshaw J. Clinical guidelines: potential benefits, limitations, and harms of clinical guidelines. *BMJ.* 1999;318(7182):527-530.
3. Qaseem A, Forland F, Macbeth F, et al. Guidelines International Network: toward international standards for clinical practice guidelines. *Ann Intern Med.* 2012;156(7):525-531.
4. Force USPST. *U.S. Preventive Services Task Force Procedure Manual.* 2015.
5. Higgins JP, Altman DG. Assessing risk of bias in included studies. Cochrane handbook for systematic reviews of interventions. *Wiley.* 2008:187-241.
6. Higgins JP, Altman DG, Gotzsche PC, et al. The Cochrane Collaboration's tool for assessing risk of bias in randomised trials. *BMJ.* 2011;343:d5928.
7. Sterne JAC, Savovic J, Page MJ, et al. RoB 2: a revised tool for assessing risk of bias in randomised trials. *Bmj.* 2019;366:l4898.
8. Sterne JA, Hernan MA, Reeves BC, et al. ROBINS-I: a tool for assessing risk of bias in non-randomised studies of interventions. *BMJ.* 2016;355:i4919.
9. National Heart, Lung, and Blood Institute. (2019). Study Quality Assessment Tools. Quality Assessment Tool for Case Series Studies. https://www.nhlbi.nih.gov/health-topics/study-quality-assessment-tools. In.
10. Hayden JA, van der Windt DA, Cartwright JL, Cote P, Bombardier C. Assessing bias in studies of prognostic factors. *Ann Intern Med.* 2013;158(4):280-286.
11. Whiting PF, Rutjes AW, Westwood ME, et al. QUADAS-2: a revised tool for the quality assessment of diagnostic accuracy studies. *Ann Intern Med.* 2011;155(8):529-536.
12. Shea BJ, Reeves BC, Wells G, et al. AMSTAR 2: a critical appraisal tool for systematic reviews that include randomised or non-randomised studies of healthcare interventions, or both. *BMJ.* 2017;358:j4008.
13. Tierney JF, Vale C, Riley R, et al. Individual Participant Data (IPD) Meta-analyses of Randomised Controlled Trials: Guidance on Their Use. *PLoS Med.* 2015;12(7):e1001855.
14. Stewart LA, Clarke M, Rovers M, et al. Preferred Reporting Items for Systematic Review and Meta-Analyses of individual participant data: the PRISMA-IPD Statement. *JAMA.* 2015;313(16):1657-1665.
15. Dettori JR, Skelly AC, Brodt ED. Critically Low Confidence in the Results Produced by Spine Surgery Systematic Reviews: An AMSTAR-2 Evaluation From 4 Spine Journals. *Global Spine J.* 2020;10(5):667-673.
16. Dettori JR, Norvell DC. Discordant Systematic Reviews: Which to Believe? *Global Spine J.* 2020;10(2):237-239.
17. In: *Methods Guide for Effectiveness and Comparative Effectiveness Reviews.* Rockville (MD)2008.
18. Foroutan F, Guyatt G, Zuk V, et al. GRADE Guidelines 28: Use of GRADE for the assessment of evidence about prognostic factors: rating certainty in identification of groups of patients with different absolute risks. *J Clin Epidemiol.* 2020;121:62-70.
19. Iorio A, Spencer FA, Falavigna M, et al. Use of GRADE for assessment of evidence about prognosis: rating confidence in estimates of event rates in broad categories of patients. *Bmj.* 2015;350:h870.
20. Atkins D, Best D, Briss PA, et al. Grading quality of evidence and strength of recommendations. *Bmj.* 2004;328(7454):1490.
21. Balshem H, Helfand M, Schunemann HJ, et al. GRADE guidelines: 3. Rating the quality of evidence. *J Clin Epidemiol.* 2011;64(4):401-406.

For Peer Review

22. Guyatt GH, Oxman AD, Kunz R, et al. GRADE guidelines 6. Rating the quality of evidence--imprecision. *J Clin Epidemiol.* 2011;64(12):1283-1293.
23. Guyatt GH, Oxman AD, Kunz R, et al. GRADE guidelines: 8. Rating the quality of evidence--indirectness. *J Clin Epidemiol.* 2011;64(12):1303-1310.
24. Guyatt GH, Oxman AD, Kunz R, et al. GRADE guidelines: 7. Rating the quality of evidence--inconsistency. *J Clin Epidemiol.* 2011;64(12):1294-1302.
25. Guyatt GH, Oxman AD, Montori V, et al. GRADE guidelines: 5. Rating the quality of evidence--publication bias. *J Clin Epidemiol.* 2011;64(12):1277-1282.
26. Guyatt GH, Oxman AD, Vist G, et al. GRADE guidelines: 4. Rating the quality of evidence--study limitations (risk of bias). *J Clin Epidemiol.* 2011;64(4):407-415.
27. Guyatt GH, Oxman AD, Sultan S, et al. GRADE guidelines: 9. Rating up the quality of evidence. *J Clin Epidemiol.* 2011;64(12):1311-1316.
28. Shiffman RN, Michel G. Toward improved guideline quality: using the COGS statement with GEM. *Stud Health Technol Inform.* 2004;107(Pt 1):159-163.
29. Shiffman RN, Shekelle P, Overhage JM, Slutsky J, Grimshaw J, Deshpande AM. Standardized reporting of clinical practice guidelines: a proposal from the Conference on Guideline Standardization. *Ann Intern Med.* 2003;139(6):493-498.
30. Vernooij RW, Alonso-Coello P, Brouwers M, Martinez Garcia L, CheckUp P. Reporting Items for Updated Clinical Guidelines: Checklist for the Reporting of Updated Guidelines (CheckUp). *PLoS Med.* 2017;14(1):e1002207.
31. Li SA, Alexander PE, Reljic T, et al. Evidence to Decision framework provides a structured "roadmap" for making GRADE guidelines recommendations. *J Clin Epidemiol.* 2018;104:103-112.
32. Alonso-Coello P, Oxman AD, Moberg J, et al. GRADE Evidence to Decision (EtD) frameworks: a systematic and transparent approach to making well informed healthcare choices. 2: Clinical practice guidelines. *BMJ.* 2016;353:i2089.
33. Alonso-Coello P, Schunemann HJ, Moberg J, et al. GRADE Evidence to Decision (EtD) frameworks: a systematic and transparent approach to making well informed healthcare choices. 1: Introduction. *BMJ.* 2016;353:i2016.
34. Guyatt GH, Oxman AD, Kunz R, et al. Going from evidence to recommendations. *BMJ.* 2008;336(7652):1049-1051.
35. Andrews JC, Schunemann HJ, Oxman AD, et al. GRADE guidelines: 15. Going from evidence to recommendation-determinants of a recommendation's direction and strength. *J Clin Epidemiol.* 2013;66(7):726-735.
36. Tetreault LA, Skelly AC, Dettori JR, Wilson JR, Martin AR, Fehlings MG. Guidelines for the Management of Degenerative Cervical Myelopathy and Acute Spinal Cord Injury: Development Process and Methodology. *Global Spine J.* 2017;7(3 Suppl):8S-20S.
37. Andrews J, Guyatt G, Oxman AD, et al. GRADE guidelines: 14. Going from evidence to recommendations: the significance and presentation of recommendations. *J Clin Epidemiol.* 2013;66(7):719-725.
38. Brouwers MC, Kho ME, Browman GP, et al. Development of the AGREE II, part 2: assessment of validity of items and tools to support application. *CMAJ.* 2010;182(10):E472-478.
39. Brouwers M KM, Browman GP, Cluzeau F, feder G, Fervers B, Hanna S, Makarski J on behalf of the AGREE Next Steps Consortium. AGREE II: Advancing guideline development, reporting and evaluation in healthcare. 2010;182:E839-842. https://www.agreetrust.org/wp-content/uploads/2013/10/AGREE-II-Users-Manual-and-23-item-Instrument_2009_UPDATE_2013.pdf.
40. Shekelle P, Woolf S, Grimshaw JM, Schunemann HJ, Eccles MP. Developing clinical practice guidelines: reviewing, reporting, and publishing guidelines; updating guidelines; and the emerging issues of enhancing guideline implementability and accounting for comorbid conditions in guideline development. *Implement Sci.* 2012;7:62.

For Peer Review

For Peer Review

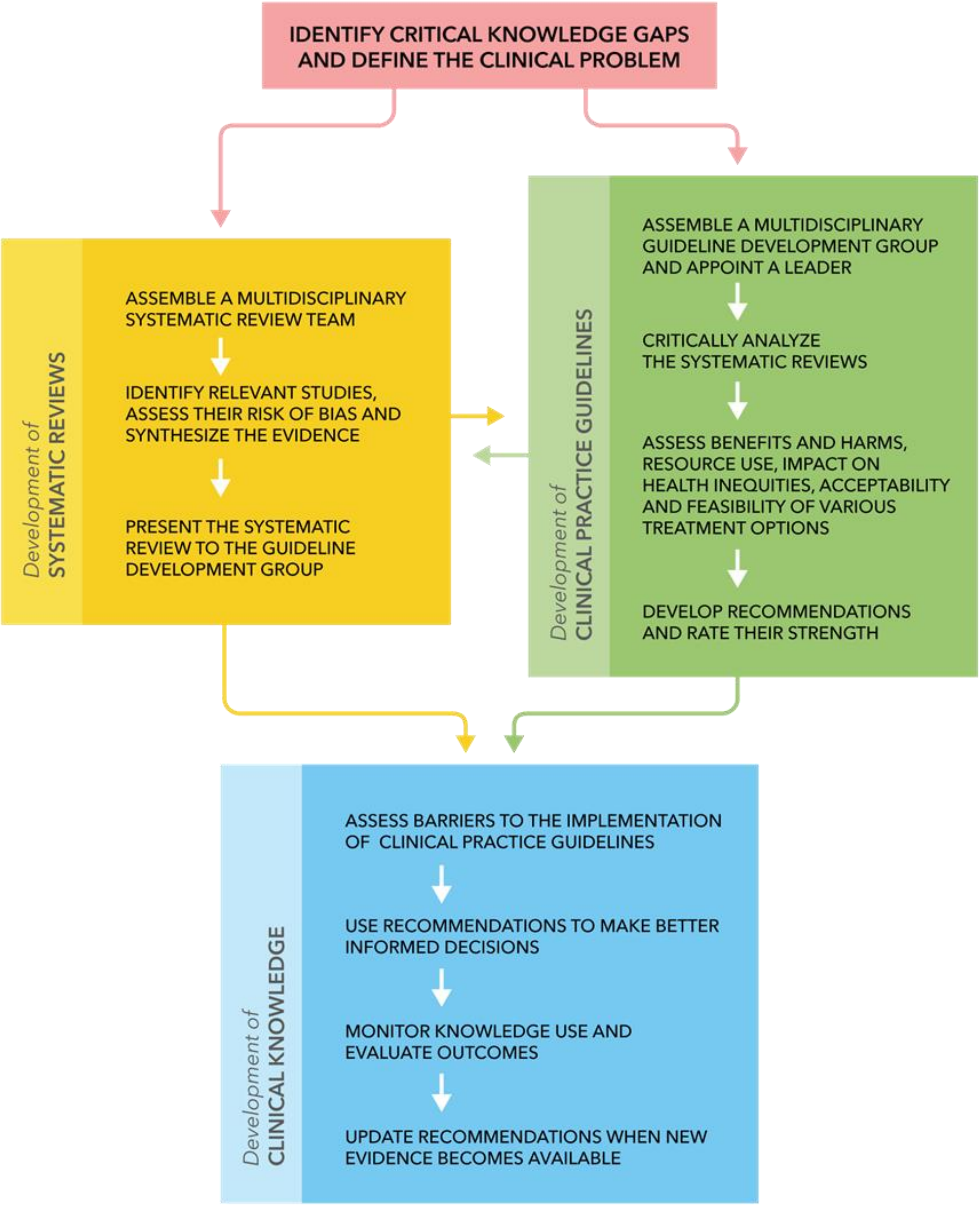
IDENTIFY CRITICAL KNOWLEDGE GAPS AND DEFINE THE CLINICAL PROBLEM
Development of SYSTEMATIC REVIEWS
ASSEMBLE A MULTIDISCIPLINARY SYSTEMATIC REVIEW TEAM
IDENTIFY RELEVANT STUDIES, ASSESS THEIR RISK OF BIAS AND SYNTHESIZE THE EVIDENCE
PRESENT THE SYSTEMATIC REVIEW TO THE GUIDELINE DEVELOPMENT GROUP
Development of CLINICAL PRACTICE GUIDELINES
ASSEMBLE A MULTIDISCIPLINARY GUIDELINE DEVELOPMENT GROUP AND APPOINT A LEADER
CRITICALLY ANALYZE THE SYSTEMATIC REVIEWS
ASSESS BENEFITS AND HARMS, RESOURCE USE, IMPACT ON HEALTH INEQUITIES, ACCEPTABILITY AND FEASIBILITY OF VARIOUS TREATMENT OPTIONS
DEVELOP RECOMMENDATIONS AND RATE THEIR STRENGTH
Development of CLINICAL KNOWLEDGE
ASSESS BARRIERS TO THE IMPLEMENTATION OF CLINICAL PRACTICE GUIDELINES
USE RECOMMENDATIONS TO MAKE BETTER INFORMED DECISIONS
MONITOR KNOWLEDGE USE AND EVALUATE OUTCOMES
UPDATE RECOMMENDATIONS WHEN NEW EVIDENCE BECOMES AVAILABLE

Table 1. An Overview of the Responsibilities of the Leadership Group

**Position**	**Responsibilities**	**Person**
Chair	• Oversee the guideline development process<br>• Chair and facilitate online and in-person meetings<br>○ Enforce adherence to the guideline protocol<br>○ Ensure balanced discussions<br>○ Facilitate consensus development<br>• Enforce and manage conflicts of interest throughout the process<br>• Ensure guidelines reflect the current body of evidence & range of stakeholder perspectives<br>• Ensure that the final product meets accepted standards<br>• Appropriately utilize panel expertise<br>• Oversee response to peer and external review<br>• Oversee the development of the executive summary<br>• Review final documents prior to submission	Michael Fehlings, Brian Kwon
Leader of Guideline Development Group	• Ensure that all participants submit relevant disclosures<br>• Develop strawman recommendations for each guideline topic<br>• Facilitate group discussions and lead the voting process using the GRADE “evidence to recommendation” framework<br>• Ensure all manuscripts are available for review by the GDG<br>• Manage guideline document and enforce adherence to the guideline protocol<br>• Assist with writing the guideline document and reviewing revisions proposed by the GDG<br>• Appraise the guidelines using AGREE II<br>• Assist with final edits of the guideline document	Lindsay Tetreault
Leader of Systematic Review Group	• Develop key clinical questions to be addressed by the systematic review<br>• Provide clinical expertise to support the methodologists<br>• Support the clinical authors and methodologists to ensure timelines are met<br>• Address any concerns regarding conflicts of interest<br>• Present the results of the systematic reviews to the larger guideline development group<br>• Assist with manuscript writing	Nathan Evaniew

For Peer Review

Table 2. Members of the Guideline Development Group

Name	Institution	Guideline
Spine Surgery (Neurosurgery, Orthopedic Surgery)		
Bizhan Aarabi	University of Maryland	Timing of Surgery, hemodynamic management
Paul Arnold	University of Illinois – Urbana-Champaign	Timing of Surgery, hemodynamic management, intraoperative spinal cord injury
Saumayajit Basu	Kothari Medical Center	Intraoperative spinal cord injury
Dean Chou	Columbia University	Intraoperative spinal cord injury
Nathan Evaniew	University of Calgary	Timing of Surgery, hemodynamic management, intraoperative spinal cord injury
Michael Fehlings	University of Toronto	Timing of Surgery, hemodynamic management, intraoperative spinal cord injury
Mario Ganau	Oxford University	Timing of Surgery, hemodynamic management
Yoon Ha	Yonsei University	intraoperative spinal cord injury
James Harrop	Thomas Jefferson University	Hemodynamic management
Gregory Hawryluk	Cleveland Clinic	Timing of Surgery, hemodynamic management, intraoperative spinal cord injury
Christoph Hofstetter	University of Washington	Timing of Surgery
Mark Kotter	University of Cambridge	Hemodynamic management
Shekar Kurpad	Froedtert and the Medical College of Wisconsin	Timing of Surgery
Brian Kwon	University of British Columbia	Timing of Surgery, hemodynamic management, intraoperative spinal cord injury
Ilya Laufer	New York University Langone Heath	Intraoperative spinal cord injury
Allan Martin	University of California, Davis Health	Hemodynamic management, intraoperative spinal cord injury
Yi Lu	Brigham and Women's Hospital	Timing of Surgery
Narihito Nagoshi	Keio University School of Medicine	Timing of Surgery, hemodynamic management
Hiroaki Nakashima	Nagoya University	Timing of Surgery
Chris Neal	Bethesda Maryland	Timing of Surgery, hemodynamic management
Vafa Rahimi-Movaghar	Tehran University of Medical Sciences	Timing of Surgery, hemodynamic management, intraoperative spinal cord injury
Ricardo Rodrigues Pinto	Universidade Do Porto	Timing of Surgery, intraoperative spinal cord injury
Rajiv Saigal	University of California – San Francisco	Timing of Surgery, hemodynamic management, intraoperative spinal cord injury
Uzma Samadani	University of Minnesota	Timing of Surgery
Valerie ter Wengel	Haaglanden Medisch Centrum	Timing of Surgery
Jefferson Wilson	University of Toronto	Timing of Surgery, hemodynamic management
Neurology		

For Peer Review

Samuel Strantzas	The Hospital for Sick Children	Intraoperative spinal cord injury
Lindsay Tetreault	New York University Langone Heath	Timing of Surgery, hemodynamic management, intraoperative spinal cord injury
Carl Zipser	Balgrist University Hospital	Timing of Surgery, hemodynamic management, intraoperative spinal cord injury
Critical or Neurocritical Care		
Nina Glass	University Hospital - Newark	Hemodynamic management
David Ethan Kahn	New York University Langone Heath	Timing of Surgery, hemodynamic management
Stephen McKenna	Stanford University	Timing of Surgery, hemodynamic management, intraoperative spinal cord injury
Emergency Medicine (and Neurocritical Care)		
Virginia Newcombe	University of Cambridge	Timing of Surgery, hemodynamic management, intraoperative spinal cord injury
Physical Medicine and Rehabilitation		
Steven Kirshblum	Kessler Institute for Rehabilitation	Timing of Surgery, Intraoperative spinal cord injury
Radha Korupolu	UTHealth Houston	Hemodynamic management
Individuals Living with Spinal Cord Injury		
Sam Douglas	Praxis Spinal Cord Institute	Timing of Surgery, hemodynamic management, intraoperative spinal cord injury
Rex Marco	Christopher and Dana Reeve Foundation	Timing of Surgery, hemodynamic management, intraoperative spinal cord injury

For Peer Review

Table 3. Key Questions Included in Each Systematic Review

Systematic, Scoping or Narrative Review Topic	Key Questions
Timing of Surgical Decompression	**Key Question 1**: What is the effectiveness of early decompression (≤ 24 hours) compared with late decompression (>24 hours) or conservative therapy based on clinically important changes in neurological status? What is the effectiveness of ultra-early decompression compared with other "early" time frames up to 24 hours (e.g., < 8 hours vs ≥ 8 hours but <24 hours)?<br>**Key Question 2:** How does timing of decompression influence other functional outcomes or administrative outcomes?<br>**Key Question 3:** What is the safety profile of early decompression compared with late decompression?<br>**Key Question 4**: Does early decompression have differential efficacy or safety in specific subgroups of patients?<br>**Key Question 5**: What is the cost-effectiveness of early decompression compared with late decompression?
Hemodynamic Management	**Key Question 1:** In patients with acute traumatic SCI, what are the effects of goal-directed interventions to optimize spinal cord perfusion on extent of neurological recovery and rates of adverse events at any time point of follow-up?<br>**Key Question 2:** In patients with acute traumatic SCI, what are the effects of particular monitoring techniques, perfusion ranges, pharmacological agents, and durations of treatment on extent of neurological recovery and rates of adverse events at any time point of follow-up?
Intraoperative Spinal Cord Injury	**Conceptual Question 1:** What definitions or monitoring thresholds have been used to define/determine ISCI and what is the reported frequency of ISCI?<br>**Key Question 1:** What are the risk factors for the development of an ISCI?
	**Key Question 1:** What is the accuracy of neurophysiological monitoring for diagnosis of ISCI compared with immediate postoperative clinical assessment?

For Peer Review

Table 4. The GRADE Approach for Assessing the Overall Quality of Evidence: Reasons for Downgrading and Upgrading

**Factor**	**Examples**	**Consequence**
Factors than can downgrade the quality of evidence		
Limitations in study design or execution (risk of bias)	• *RCT:* inadequate randomization sequence, lack of allocation concealment, lack of blinding, incomplete accounting of patients and outcome events, selective outcome reporting<br>• *Observational studies:* failure to develop and apply appropriate eligibility criteria, failure to control confounding, flawed measurement of both exposure and outcome, incomplete or inadequately short follow-up, selective outcome reporting	Downgrade 1 or 2 levels
Inconsistency of results	*Unexplained heterogeneity of results across studies:*<br>• Wide variance of point estimates across studies<br>• Effect estimates in the opposite directions leading to different clinical conclusions<br>• Minimal or no overlap of confidence intervals<br>• Statistical criteria (e.g. tests of heterogeneity)	Downgrade 1 or 2 levels
Indirectness of evidence*	*Sources of indirectness include*<br>• Directness of outcome measures (patient centered outcomes are considered direct, intermediate outcomes are not.)<br>• Indirect comparisons	Downgrade 1 or 2 levels
Imprecision	• Wide confidence intervals<br>• Confidence interval ranges that cross null and thresholds for clinically important effects<br>• Consideration of sample size to detect outcomes (e.g., rare versus common outcomes)	Downgrade 1 or 2 levels
Publication and reporting bias	• Selective reporting of outcomes/findings within studies<br>• Selective publication of "positive" results<br>• Selective rejection of manuscripts with "negative" results	Downgrade 1 or 2 levels
Factors that can upgrade the quality of evidence (observational studies, assuming no downgrade for the above)		
Large magnitude of effect	• *Very large*: RR>5 or <0.2<br>• *Large*: RR>2 or <0.5<br>• Decision includes consideration of confidence interval width and overlap and whether effects are smaller than a chosen threshold	Upgrade 1 or 2 levels
All plausible confounding would reduce the demonstrated effect or increase the effect if no effect was observed	• This is not common; most observational studies are likely to have unadjusted residual confounding/bias	Upgrade 1 level

For Peer Review

Dose-response gradient	• Consistent increase or decrease in effect estimate for an outcome based on "dose" of intervention (test for dose-response)	Upgrade 1 level

For Peer Review

Table 5. Interpretation of the Grading of the Evidence

Grade	Definition
High	High confidence in the effect estimate.<br>The true effect lies close to that of the estimate of effect.
Moderate	Moderate confidence in the effect estimate.<br>The true effect is likely to be close to the estimate of effect, but there is a possibility that it is substantially different.
Low	Limited confidence in the effect estimate.<br>The true effect may be substantially different from the estimate of effect.
Very Low	Very little confidence in the effect estimate.<br>The true effect is likely to be substantially different from the estimate of effect.

For Peer Review

Table 6. The GRADE Evidence to Recommendation Framework

Question	Response Options
Benefits and Harms	
What is the overall certainty of the evidence?	No included studies, very low, low, moderate, high
Is there important uncertainty about how much people value the main outcomes?	Important uncertainty or variability, possibly important uncertainty or variability, probably no important uncertainty or variability, no important uncertainty or variability, no known undesirable
How substantial are the desirable anticipated effects ?	Trivial, small, moderate, large, varies, don't know
How substantial are the undesirable anticipated effects ?	Trivial, small, moderate, large, varies, don't know
Does the balance between desirable and undesirable effects favor the intervention or the comparison?	Favors comparison, probably favors comparison, does not favor either the intervention or the comparison, probably favors intervention, favors intervention, varies, don't know
Resource Use	
How large are the resource requirements (costs)?	Large costs, moderate costs, negligible costs and savings, moderate savings, large savings, varies, don't know
What is the certainty of the evidence of resource requirements (costs)?	No included studies, very low, low, moderate, high
Does the cost-effectiveness of the intervention favor the intervention or the comparison?	Favors comparison, probably favors comparison, does not favor either the intervention or the comparison, probably favors intervention, favors intervention, varies, don't know
Equity	
What would be the impact on health inequities?	Increased, probably increased, uncertain, probably reduced, reduced, varies
Acceptability	
Is the option acceptable to key stakeholders?	No, probably no, uncertain, probably yes, yes, varies
Feasibility	
Is the option feasible to implement?	No, probably no, uncertain, probably yes, yes, varies
Balance of the Consequences	
What is the balance between undesirable and desirable consequences?	Undesirable consequences clearly outweigh desirable consequences, undesirable consequences probably outweigh desirable consequences in most settings, the balance between desirable and undesirable consequences is closely balanced or uncertain, desirable consequences probably outweigh undesirable consequences in most settings, desirable consequences clearly outweigh undesirable consequences in most settings
Type of Recommendation	
What is the strength and direction of the recommendation?	We recommend against offering this option, we suggest not offering this option, we suggest offering this option, we recommend offering this option
Justification	What is the justification for the recommendation, based on the criteria in the framework that drove the recommendation?

For Peer Review

For Peer Review

Table 7. The Appraisal of Guidelines for Research and Evaluation II

Domain	Key Items
1. Scope and Purpose	• The overall objective(s) of the guideline is (are) specifically described.<br>• The health question(s) covered by the guideline is (are) specifically described.<br>• The population to whom the guideline is meant to apply is specifically described.
2. Stakeholder Involvement	• The guideline development group includes individuals from all relevant professional groups.<br>• The views and preferences of the target population have been sought.<br>• The target users of the guideline are clearly defined.
3. Rigour of Development	• Systematic methods were used to search for evidence.<br>• The criteria for selecting the evidence are clearly described.<br>• The strengths and limitations of the body of evidence are clearly described.<br>• The methods for formulating the recommendations are clearly described.<br>• The health benefits, side effects, and risks have been considered in formulating the recommendations.<br>• There is an explicit link between the recommendations and the supporting evidence.<br>• The guideline has been externally reviewed by experts prior to its publication.<br>• A procedure for updating the guideline is provided.
4. Clarity of Presentation	• The recommendations are specific and unambiguous.<br>• The different options for management of the condition of health issue are clearly presented.<br>• Key recommendations are easily identifiable.
5. Applicability	• The guideline describes facilitators and barriers to its application.<br>• The guideline provides advice and/or tools on how the recommendations can be put into practice.<br>• The potential resource implications of applying the recommendations have been considered.<br>• The guideline presents monitoring and/or auditing criteria.
6. Editorial Independence	• The views of the funding body have not influenced the content of the guideline.<br>• Competing interests of guideline development group members have been recorded and addressed.

For Peer Review
