# Supplementary material for: The 2023 AO Spine-Praxis Guidelines in Acute Spinal Cord Injury: What Have We Learned? What Are the Critical Knowledge Gaps and Barriers to Implementation?
Source: Global Spine J. 2024 Mar 25;14(3 Suppl):223S–230S. doi: 10.1177/21925682231196825 (PMC10964887; doi:10.1177/21925682231196825)
Supplement: Supplemental Material - The 2023 AO Spine-Praxis Guidelines in Acute Spinal Cord Injury: What Have We Learned? What Are the Critical Knowledge Gaps and Barriers to Implementation? [file sj-pdf-4-gsj-10.1177_21925682231196825.pdf]

A Clinical Practice Guideline for the Management of Patients with Acute Spinal Cord Injury: Recommendations on Hemodynamic Management

Journal:	*Global Spine Journal*
Manuscript ID	Draft
Manuscript Type:	Special Issue- Knowledge Forum SCI Guidelines
Keywords:	spinal cord injury, neuro, trauma

Abstract:

Study Design
Clinical practice guideline development following the GRADE process.
Objectives
Hemodynamic management is one of the only available treatment options that likely improves neurologic outcomes in patients with acute traumatic spinal cord injury (SCI). Augmenting mean arterial pressure (MAP) aims to improve blood perfusion and oxygen delivery to the injured spinal cord in order to minimize secondary ischemic damage to neural tissue. The objective of this guideline was to update the 2013 AANS/CNS recommendations on the hemodynamic management of patients with acute traumatic SCI, acknowledging that much has been published in this area since its publication. Specifically, we sought to make recommendations on 1. the range of mean arterial pressure (MAP) to be maintained by identifying an upper and lower MAP limit; 2. the duration of such MAP augmentation; and 3. the choice of vasopressor. Additionally, we sought to make a recommendation on spinal cord perfusion pressure (SCPP) targets.
Methods
A multidisciplinary guideline development group (GDG) was formed that included healthcare professionals from a wide range of clinical specialities, patient advocates, and individuals living with SCI. The GDG reviewed the 2013 AANS/CNS guidelines and voted on whether each recommendation should be endorsed or updated. A systematic review of the literature, following PRISMA standards and registered in PROSPERO, was conducted to inform the guideline development process and address the following key questions: (i) what are the effects of goal-directed interventions to optimize spinal cord perfusion on extent of neurological recovery and rates of adverse...

SCHOLARONE™
Manuscripts

ABSTRACT

Study Design

Clinical practice guideline development following the GRADE process.

Objectives

Hemodynamic management is one of the only available treatment options that likely improves neurologic outcomes in patients with acute traumatic spinal cord injury (SCI). Augmenting mean arterial pressure (MAP) aims to improve blood perfusion and oxygen delivery to the injured spinal cord in order to minimize secondary ischemic damage to neural tissue. The objective of this guideline was to update the 2013 AANS/CNS recommendations on the hemodynamic management of patients with acute traumatic SCI, acknowledging that much has been published in this area since its publication. Specifically, we sought to make recommendations on 1. the range of mean arterial pressure (MAP) to be maintained by identifying an upper and lower MAP limit; 2. the duration of such MAP augmentation; and 3. the choice of vasopressor. Additionally, we sought to make a recommendation on spinal cord perfusion pressure (SCPP) targets.

Methods

A multidisciplinary guideline development group (GDG) was formed that included healthcare professionals from a wide range of clinical specialities, patient advocates, and individuals living with SCI. The GDG reviewed the 2013 AANS/CNS guidelines and voted on whether each recommendation should be endorsed or updated. A systematic review of the literature, following PRISMA standards and registered in PROSPERO, was conducted to inform the guideline development process and address the following key questions: (i) what are the effects of goal-directed interventions to optimize spinal cord perfusion on extent of neurological recovery and rates of adverse events at any time point of follow-up? and (ii) what are the effects of particular monitoring techniques, perfusion ranges, pharmacological agents, and durations of treatment on extent of neurological recovery and rates of adverse events at any time point of follow-up? The GDG combined the information from this systematic review with their clinical expertise in order to develop recommendations on a MAP target range (specifically an upper and lower limit to target), the optimal duration for MAP augmentation, and the use of vasopressors or inotropes. Using methods outlined by the GRADE working group, recommendations were formulated that

For Peer Review

considered the balance of benefits and harms, financial impact, acceptability, feasibility and patient preferences.

Results

The GDG suggested that MAP should be augmented to at least 75-80 mmHg as the "lower limit", but not actively augmented beyond an "upper limit" of 90-95 mmHg in order to optimize spinal cord perfusion in acute traumatic SCI. The quality of the evidence around the "target MAP" was very low, and thus the strength of this recommendation is weak. For duration of hemodynamic management, the GDG "suggested" that MAP be augmented for a duration of 3-7 days. Again, the quality of the evidence around the duration of MAP support was very low, and thus the strength of this recommendation is also weak. The GDG felt that a recommendation on the choice of vasopressor or the use of SCPP targets was not warranted, given the dearth of available evidence.

Conclusion

We provide new recommendations for blood pressure management after acute SCI that acknowledge the limitations of the current evidence on the relationship between MAP and neurologic recovery. It was felt that the low quality of existing evidence and uncertainty around the relationship between MAP and neurologic recovery justified a greater range of MAP to target, and for a broader range of days post-injury than recommended in previous guidelines. While important knowledge gaps still remain regarding hemodynamic management, these recommendations represent current perspectives on the role of MAP augmentation for acute SCI.

For Peer Review

SUMMARY OF RECOMMENDATIONS

For the Target Range of Mean Arterial Pressure Augmentation

Population: Adult patients with acute traumatic SCI.

Key question: Should we recommend the augmentation of mean arterial blood pressure to at least 75-80mmHg and not higher than 90-95 mmHg in order to optimize spinal cord perfusion in acute traumatic SCI?

Recommendation 1: We suggest the augmentation of mean arterial blood pressure to at least 75-80mmHg but not higher than 90-95 mmHg in order to optimize spinal cord perfusion in acute traumatic SCI.

Quality of Evidence: Very Low

Strength of Recommendation: Weak

For the Duration of Mean Arterial Pressure Augmentation

Population: Adult patients with acute traumatic SCI.

Key question: Should we recommend the augmentation of mean arterial blood pressure for a duration of 3-7 days in order to optimize spinal cord perfusion in acute traumatic SCI?

Recommendation 1: We suggest the augmentation of mean arterial pressure for a duration of 3-7 days in order to optimize spinal cord perfusion in acute traumatic SCI.

Quality of Evidence: Very Low

Strength of Recommendation: Weak

For the Vasopressor/Inotrope Used for Mean Arterial Pressure Augmentation

Population: Adult patients with acute traumatic SCI.

Key question: Should we recommend the use of a specific vasopressor in order to achieve MAP-directed goals in patients with acute traumatic SCI?

Statement: The decision should be left to the attending physician in terms of what vasopressor or inotrope to use in order to achieve MAP-directed goals in patients with acute traumatic SCI.

For Peer Review

INTRODUCTION

Acute traumatic spinal cord injury (SCI) can result in catastrophic loss of neurologic function. There are limited options for improving neurologic function in SCI patients outside of urgent surgical decompression (the subject of an accompanying guideline in this focus issue), and hemodynamic management. Ultimately, both of these treatments aim to enhance vascular perfusion and oxygen delivery to the injured spinal cord in an effort to minimize secondary ischemic damage to vulnerable neural tissue and thus improve neurologic recovery. From a purely practical perspective, there is a strong rationale to optimize tissue hemodynamics given that this is one of the only non-operative interventions available to clinicians managing patients with acute SCI.

It is widely accepted that monitoring of acute SCI patients should be done in an intensive care unit (ICU) or high acuity setting if available, where their cardiopulmonary status can be evaluated on an ongoing basis and managed judiciously.[1] Furthermore, systemic hypotension, defined as a systolic blood pressure of less than 90 mmHg, can be deleterious to the spinal cord (and for the patient in general) and so should be avoided or corrected. To some extent, these are basic principles of acute trauma management that are applicable to SCI patients who can also suffer additional injuries leading to hemorrhagic or cardiogenic shock, or may experience neurogenic shock in conjunction with their neurologic injury.

In addition to ICU monitoring and avoiding systemic hypotension, several studies and clinical practice guidelines have recommended that the mean arterial pressure (MAP) be augmented in patients with acute traumatic SCI. The works of Levi et al. and Vale et al. have been cited by many to substantiate a MAP target of at least 90 or above 85 mmHg, respectively.[2,3] Furthermore, initial guidelines by the Consortium of Spinal Cord Medicine in 2008 recommended that MAP be maintained above 85 mmHg for 7 days.[4] Similarly, the 2013 AANS/CNS guidelines reviewed the literature up to 2011 and recommended that the MAP be maintained "between 85 to 90 mmHg for 7 days".[1]

A number of factors were considered when revisiting the 2013 AANS/CNS recommendation that MAP be maintained between 85 to 90 mmHg for 7 days.[1] First, it was recognized that attention to this topic was warranted, given that MAP augmentation and hemodynamic management is one

For Peer Review

of the few treatment options available for acute SCI, and because several studies have been added to the literature since 2011 (the cut-off for the literature review in the 2013 AANS/CNS guideline). Specifically, many authors have reported on the relationship between MAP and neurologic outcome since 2011, and a new field of monitoring spinal cord perfusion pressure (SCPP) has emerged since the 2013 AANS/CNS guideline.[5-8]

Second, it was acknowledged that choosing a specific MAP target was important given that small changes in MAP and SCPP can significantly affect the human spinal cord, given its susceptibility to ischemia. The empirical evidence of this concept is embodied in our reaction to a loss of, or significant drop, in intraoperative neuromonitoring (IONM) signals, indicating potential spinal cord impairment and damage. In most cases, the first line of treatment is to immediately increase the MAP to facilitate spinal cord perfusion, which often improves spinal cord neurophysiologic function.[9] Virtually all spine surgeons have witnessed the exquisite sensitivity of the human spinal cord at some point in their career and have experienced profound relief after averting an impending neurologic deficit by simply increasing the blood pressure. Further, in thoracoabdominal aortic aneurysm repair surgery, there is an inherent risk of ischemic injury to the spinal cord due to disruption of the key segmental vasculature.[10] However, this risk of ischemic SCI is lowered by draining cerebrospinal fluid (CSF), reducing intrathecal pressure and improving SCPP.[11] This supports the concept that the human spinal cord is sensitive to small changes in blood flow and that maintaining its perfusion can prevent significant ischemic injury in some patients. Furthermore, investigators have demonstrated that the average MAP differed by only 2-3 mmHg over the first 3 days between SCI patients who failed to recover neurologically and those who improved by ≥1 grade on the ASIA Impairment Scale (AIS), again suggesting that the acutely injured spinal cord is highly sensitive to changes in perfusion.[12] Collectively, this empiric evidence supports the idea that the human spinal cord is very susceptible to ischemia generated by decreases in blood pressure and SCPP.

The third consideration for revising the 2013 AANS/CNS guideline on hemodynamic management was based on current challenges in both the interpretation and implementation of the proposed MAP target. The 2013 guideline did not recommend that the MAP be augmented to "at least 85 mmHg" but rather stated that the MAP be maintained "between 85 and 90 mmHg for the first 7 days" after SCI. The wording of the 2013 guidelines may have been unintentional given that the

For Peer Review

cited research at that time did not study such a narrow range. Moreover, this MAP target range of 5 mmHg – even if it were the "optimal MAP for the spinal cord" - is almost impossible to maintain, given the variability of blood pressures in acute SCI patients and because such precise control cannot be afforded by conventional vasopressors or inotropes.[13] As maintaining a MAP in the 5 mmHg range between 85 and 90 mmHg is often not feasible, clinicians have adopted their own practices, ostensibly "based on the guidelines," such as keeping the MAP at or above 85 mmHg (i.e. no upper limit)[14] or targeting the MAP between 85 and 95 mmHg.[15] Obviously, interpreting the 2013 AANS/CNS guidelines in these manners may not pose any specific harm to patients, but the variations in interpretation do reveal that the previous recommendations were not strictly adhered to, even by centers that are very involved in hemodynamic management research. Given the variability in implementation, revising the 2013 guidelines seemed warranted in order to develop recommendations that are clearer, more feasible, and thus more widely implemented.

Acknowledging the aforementioned points, we undertook an initiative to update the 2013 guidelines to determine if new recommendations on the hemodynamic management of SCI were warranted. With the support of AO Spine and the Praxis Spinal Cord Institute, we were able to establish the evidence base through a systematic review of the literature that adhered to current methodological standards and develop a clinical practice guideline using the GRADE approach. An introductory article in this focus issue summarizes the rationale, scope and specific aspects of care covered by this guideline and is entitled *"AO Spine/Praxis Clinical Practice Guidelines for the Management of Acute Spinal Cord Injury: An Introduction to a Focus Issue"*.[16]

METHODS

This guideline was developed under the auspices of AO Spine and the Praxis Spinal Cord Institute. A multidisciplinary guideline development group (GDG) was formed that included healthcare professionals from a wide range of clinical specialities (neurosurgery, orthopedic surgery, trauma surgery, neurocritical care, neurology, physiatry and anesthesiology), patient advocates, and individuals living with SCI. Members of the GDG were required to disclose any intellectual or financial conflicts of interest and were editorially independent from both funding sources. The GDG reviewed the 2013 AANS/CNS guideline[1] and voted on each of the three recommendations to decide whether they should be endorsed or updated. A guideline development protocol was

For Peer Review

created using the Conference on Guideline Standardization Checklist and is available in a separate article in this focus issue.[17,18]

A systematic review of the literature was conducted by Evaniew et al.[19] to inform the guideline process and to address the following key questions: *in patients with acute traumatic SCI (i) what are the effects of goal-directed interventions to optimize spinal cord perfusion on extent of neurological recovery and rates of adverse events at any time point of follow-up?* and (ii) *what are the effects of particular monitoring techniques, perfusion ranges, pharmacological agents, and durations of treatment on extent of neurological recovery and rates of adverse events at any time point of follow-up?* This review adhered to the methods provided by the Cochrane Handbook for Systematic Reviews of Interventions and the Agency for Healthcare Research and Quality and was reported according to the Preferred Reporting Items for Systematic Reviews and Meta-Analyses (PRISMA) statement.[20,21] Furthermore, the GRADE approach was used to evaluate the overall strength of the evidence for each included outcome.[22]

The results of the systematic review were presented to the members of the GDG during a Zoom online video-conference meeting held on November 5, 2022. During this meeting, the GRADE Guideline Development Tool was used to document the process, rank the outcomes most critical for decision-making, weigh the desirable and undesirable effects of various options and determine the strength of the recommendations.[23-25] Methodologists from Aggregate Analytics assisted clinical authors in conducting the systematic review of the literature and provided expertise during the guideline development process. A threshold of 80% was considered consensus. The guideline development meeting was conducted over the Zoom videoconferencing platform which allowed for participants to vote anonymously on each question in the GRADE framework. If there were discrepancies in voting, the GDG discussed their perspectives and provided clinical expertise in an attempt to reach consensus. A detailed summary of our methodology is provided in separate articles within this focus issue.[26,27]

RESULTS

Twenty members of the GDG participated in the guideline development meeting over Zoom: eleven spine surgeons, three critical or neurocritical care physicians, two neurologists, one physiatrist, one first responder and two individuals living with SCI.

For Peer Review

Voting on the 2013 AANS/CNS guideline for hemodynamic management

The following three recommendations were published in the 2013 AANS/CNS guideline on the hemodynamic management of SCI:

1. Use of cardiac, hemodynamic, and respiratory monitoring devices to detect cardiovascular dysfunction and respiratory insufficiency in patients following acute SCI is recommended.
2. Correction of hypotension in SCI (systolic blood pressure < 90 mmHg) when possible and as soon as possible is recommended.
3. Maintenance of MAP between 85 and 90 mmHg for the first 7 days following an acute SCI is recommended.

The first question posed to the GDG was whether to simply adopt the 2013 AANS/CNS hemodynamic guideline "as is," given that the updated systematic review highlighted that the overall quality of evidence was low. Ninety percent of the GDG agreed to separately address each of the three recommendations from the 2013 AANS/CNS guideline. Of note, this part of the guideline development process was based solely on expert opinion and did not abide by the standards proposed by the GRADE Working Group.

For recommendation #1, 89% of the GDG voted to accept and endorse this 2013 statement regarding the use of cardiac, hemodynamic and respiratory monitoring devices. The GDG agreed that patients with SCI often require higher level of care and close monitoring in an ICU setting given increased rates of respiratory insufficiency, cardiac dysfunction and systemic hypotension. Patients with cervical SCI may require mechanical ventilation. Early detection of cardiopulmonary dysfunction and hemodynamic instability may allow for timely implementation of effective and life-saving strategies. The GDG acknowledged that some patients with less severe SCI, such as those with a mild "central cord syndrome" pattern of incomplete tetraplegia, may be safely monitored and managed in a step down or acute care unit. Furthermore, it was recognized that providing ICU level care for every SCI patient might not be feasible in resource-limited clinical settings (e.g. low or middle-income countries).

For Peer Review

For recommendation #2, 84% of the GDG voted to accept and endorse this 2013 statement regarding the correction of hypotension to a systolic blood pressure above 90 mmHg. As stated previously, the injured spinal cord is particularly susceptible to decreases in systolic blood pressure given impaired vascular reactivity and loss of auto-regulation. Given that small changes in the perfusion of the spinal cord can worsen ischemia and propagate secondary injury, the GDG agreed that systemic hypotension should be avoided or corrected as soon as possible. Furthermore, the GDG recognized that maintaining a systolic blood pressure over 90 mmHg represents standard of care for most patients admitted to the hospital to ensure adequate systemic perfusion and limit end-organ damage.

For Peer Review

For recommendation #3 on the MAP target of 85-90 mmHg for 7 days, 84% of the GDG voted to revise this recommendation based on the reasons outlined in the introduction. The GDG then aimed to establish a new recommendation by addressing three key components of hemodynamic management: (i) the upper and lower limits of a MAP target range; (ii) the optimal duration of MAP augmentation; and (iii) the choice of vasopressor or inotrope for pharmacologic support of MAP.

PART 1. MAP AUGMENTATION TARGET RANGE

The first part of this guideline aims to address the following key question: *Should we recommend the augmentation of MAP to at least X mmHg and not higher than Y mmHg in order to optimize spinal cord perfusion in acute traumatic SCI?* The following section will summarize how the lower limit "X" and upper limit "Y" were decided by the GDG.

Defining the MAP Range

Our approach for establishing a "target MAP" was to review the literature identified by the systematic review and summarize the studies that reported on changes in neurologic function in relation to MAP (**Table 1**). Specifically, we focused on studies that examined and reported either the MAP below which there is worse neurological recovery or the MAP above which there is no further benefit or potential harm. It is important to recognize that none of the studies were considered high quality, and none concluded a "causation" between a specific MAP target and

neurologic improvement. Instead, these studies reported only on the relationship (or lack thereof) between MAP targets and neurologic recovery, functional impairment or disability. Each study was presented during the GDG meeting to summarize the evidence for the entire group prior to voting.

Establishing the "lower MAP limit"

Fourteen studies were identified from the systematic review that analyzed neurologic outcome in relation to a "lower limit" of MAP (**Table 1**). Lower limits of MAP ranged from 50-65 mmHg to 95-104 mmHg with many studies reporting on 70 mmHg, 75 mmHg, 80 mmHg and 85 mmHg. The assessment of neurologic recovery were conducted at variable time-points post-injury as shown in Table 1, with some being done "at discharge" from acute care and others as late as 1 year post-injury.

Investigators based out of the University of California, San Francisco (UCSF) collected high-frequency MAP data at 1-minute intervals and then evaluated 80 different MAP thresholds in one mmHg increments between 40 and 120 mmHg. From this data-collection system, Catapano et al. focused on a threshold of 85 mmHg and reported that patients with AIS A, B, and C injuries who did not improve neurologically had significantly more recordings below 85 mmHg.[14] Similarly, Hawryluk et al. demonstrated that the average MAP was higher in patients who exhibited more than one grade improvement in the AIS and that AIS B, C, and D patients who did not improve had more recordings below 85 mmHg.[12] Based on an analysis of every MAP between 40 and 120 mmHg, they concluded that the lowest MAP that distinguished patients with no improvement and those with 1 grade of improvement was 70-75mmHg *"suggesting that this may be the lowest blood pressure threshold associated with neurologic benefit."* Two additional studies (not from UCSF) targeted a MAP >85 mmHg.[28,29] Dakson et al. attempted to avoid having the MAP fall below 85 mmHg for any 2-hour period over 5 day.[28] They achieved this in only 20% of their cohort, with 80% experiencing episodes of this "relative hypotension" below 85 mmHg. In this study, improvement in AIS grade was less frequent in patients who experienced these episodes of MAP<85mmHg. Weinberg et al. reviewed 136 acute SCI patients and concluded that MAP<85 mmHg was associated with a higher likelihood of not converting their AIS grade.[29]

For Peer Review

The study by Haldrup et al. evaluated a MAP threshold of 80 mmHg and reported that during the first two days of hospitalization there was a "moderate impact" between improvement in AIS grade and MAP.[30] However, Figure 1 in this paper illustrates that patients who improved, worsened, and remained the same neurologically had almost identical median MAPs on days 1-2 and days 3-7.

The study by Cohn et al. included only AIS A injuries and evaluated how many minutes each patient spent within different MAP ranges.[31] Based on their results, the percent of time spent below a MAP of 70 mmHg was negatively correlated with total motor score improvement, suggesting this as a possible lower limit. Squair et al. explored the risk of "no recovery" after injury based on the MAP and SCPP as measured by a lumbar intrathecal drain.[6] Based on their analysis, the risk of not improving by an AIS grade was above 1.0 when the MAP was below 70 mmHg, supporting this as a potential lower threshold. The study by Hogg et al. included only AIS C patients and determined that those with MAP < 75 mmHg had the lowest change in motor score; however, it is difficult to conclude what the "lower limit" would be from this analysis.[32] Finally, Ehsanian et al. leveraged high frequency (q1min) data on intra-operative blood pressure management.[33] This study concluded that MAP < 70 mmHg was associated with worse motor recovery and that outcomes were optimized when intraoperative MAP was maintained between 70 and 94 mmHg.

Five studies failed to demonstrate an association between MAP and neurologic recovery.[15,34-37] The study by Martin et al. accessed electronic records of 105 patients with SCI to compare MAP over the first 3 days to motor score recovery during hospitalization.[34] Based on their results, changes in motor score did not differ between patients who required vasopressors and those who did not or between patients who were "relatively hypotensive" and those who maintained specific MAP goals. Indeed, patients who required vasopressors were significantly more likely to have MAPs below 70 mmHg or even 65 mmHg, but ultimately demonstrated similar motor score recovery as patients not requiring vasopressors. Given these findings, no "lower limit" was established from this study. The study by Chen et al. focused on SCPP and assessed how far patients deviated from the "optimal SCPP" and a target MAP of 85-90 mmHg.[35] Based on their results, there was no significant difference in motor recovery between patients who were 10 mmHg or >10 mmHg below this target, suggesting that even a MAP less than 75 mmHg does not impact

For Peer Review

neurologic improvement. Park et al. evaluated 73 patients with cervical SCI in order to identify prognostic factors that influenced outcome.[36] This study determined that patients who arrived to the hospital with a MAP <75 mmHg did worse neurologically than those with a MAP >85 mmHg on admission. However, patients with an average MAP >85 mmHg, between 75 and 85 mmHg, or <75 mmHg for 1 week did not differ with respect to neurologic recovery. These results indicate that while arriving to the hospital with a MAP <75 mmHg is unfavorable, the average MAP over the first week of hospitalization may not influence recovery. Based on a fourth study by Saadoun et al., MAP did not correlate with AIS grade improvement.[15] Specifically, the percent of patients who improved by one or even two AIS grades was similar if the average MAP was less than 75 mmHg or between 85 and 95 mmHg. Finally, Sewell et al. examined whether the institution of a checklist in the hospital reduced the number of times that MAP was recorded below 80 mmHg or 70 mmHg.[37] While the implementation of this checklist reduced the number of episodes of "relative hypotension," there was no impact on change in AIS grade.

Unfortunately, none of these studies reported "causation" but rather the association between "poor recovery" or "lack of recovery" and a certain MAP threshold. Furthermore, most studies had small sample sizes, included a restrictive patient population (e.g. AIS C only in the Hogg study[32]) or focused only on a discrete period of time (e.g. intra-operative for the Ehsanian study[33]). As such, it is difficult to know how to effectively extrapolate this data to the broader acute SCI population. Accepting these limitations, it is apparent in our review of the literature that while some studies reported no association between MAP and neurologic recovery, others identified specific lower limits below which outcomes are affected. At centers where the institutional "target" was to maintain MAP above 85 mmHg, it is justified to evaluate whether neurologic recovery is affected by drops in MAP below this threshold. In general, analyses of data collected from these sites demonstrated that recovery was worse in patients whose MAPs were less than 85 mmHg, although Hawryluk et al. concluded that a "lower threshold" might exist even at 70-75 mmHg.[12] Based on the body of literature, while several studies demonstrated worse neurologic outcomes with a MAP below 85 mmHg, others concluded no association between neurologic recovery and MAP,[15] or suggested 75 or 80 mmHg as an important target. In essence, while the 2013 guidelines recommended a MAP range of 85-90 mmHg, the lower limit of 85 mmHg was only variably reported in the literature as the threshold below which neurologic outcome (assessed at varying time points post-injury) is worsened.

For Peer Review

Establishing the "upper MAP limit"

Three studies were identified from the systematic review that discussed the "upper limit" of the MAP target range beyond which there was no further improvement in neurologic recovery (**Table 2**). An additional five studies commented on the complications associated with MAP elevation and vasopressor or inotrope use (**Table 3**).

The study by Ehsanian et al. included 25 acute SCI patients and examined the impact of intraoperative MAP, measured at 5 mmHg increments, on neurologic recovery.[33] Based on their results, improvement in motor score was not evident in patients whose MAPs were ≥ 95 mmHg but occurred when MAP was maintained between 70-94 mmHg. Hawryluk et al. included high frequency (q1min) MAP recordings throughout the first 5 days post-injury and conducted an iterative analysis of neurologic improvement (either 0, 1, or >1 AIS grade) at 1 mmHg increments from 40 mmHg to 120 mmHg.[12] This study concluded that there was no further neurologic improvement when MAP was ≥ 95 mmHg, suggesting this as a potential upper limit. Finally, in the study by Hogg et al., improvement in motor function plateaued at 95 mmHg with no neurologic benefit at MAPs above this target.[32] Notably, one of the central arguments in this study (and many others led by Dr. Marios Papadopoulos at this institution) is that neurologic recovery is more closely associated with SCPP than it is with MAP.[15,32,35,38-41]

With respect to complications, three studies from UCSF reported high rates of adverse events (74.0% to 94.0%) when vasopressors or inotropes were used to maintain a MAP of at least 85 mmHg.[42-44] In these studies, the complications associated with the use of vasopressors or inotropes were often cardiogenic and included bradycardia, atrial fibrillation or flutter, ventricular tachycardia, elevated troponin or ST changes. In a study by Vale et al., vasopressors were used to maintain MAP above 85 mmHg; while rates of complications were not specifically reported, the authors concluded that there were "no serious adverse events" associated with MAP augmentation.[3] Finally, Weinberg et al. concluded that although maintaining MAP above 85 mmHg was not associated with an increase in adverse events, the use of vasopressors was, suggesting that this is a pharmacologic effect (and not simply related to having a higher MAP).[29]

The majority of these studies were published after the 2013 AANS/CNS guidelines. Given the availability of this new literature, we acknowledged the importance of defining an "upper limit"

For Peer Review

beyond which there is no neurologic benefit but potential complications from vasopressor or inotrope use. Unfortunately, based on the five available studies, it is not possible to discern the MAP threshold above which there is a greater risk of complications. However, it can be assumed that if an upper limit (e.g. 95 mmHg) existed beyond which there was no major neurologic benefit, then there would be no justification to continue administering vasopressors to augment MAP beyond this level given the complications associated with vasopressors and inotropes.

GDG Voting on the Lower and Upper MAP Limits

Following the presentation of the results of these individual studies, the GDG voted on both a lower and an upper limit for the MAP target. For the lower MAP limit, 32% voted 75 mmHg, 63% selected 80 mmHg, and 5% chose 85 mmHg. Of note, no member of the GDG supported 60, 65, 70 or 90 mmHg as the lower limit of the proposed MAP target. Based on the voting, a lower limit range of 75-80 mmHg was selected. The decision to reduce the lower limit of the MAP target range from 85 mmHg (as per the 2013 AANS/CNS guidelines) to 75-80 mmHg was based on the reviewed studies that (i) suggested worse neurologic outcomes with a MAP below 70, 75 or 80 mmHg or (ii) failed to demonstrate an association between neurologic recovery and MAP levels. Given the variability of results across studies and because targeting exact MAP goals can be challenging from a practical standpoint, the GDG agreed that a range of 75-80 mmHg was more appropriate for the lower limit than a single number. Furthermore, a range of 5 mmHg provides physicians with more flexibility to decide when to escalate care and initiate interventions to augment MAP, including placement of a central line and treatment with vasopressors or inotropes. This "buffer" is especially relevant in patients with concomitant traumatic injuries, hemorrhage or cardiac comorbidities where increasing MAP may carry additional risks.

For the upper MAP limit, 12% voted 90 mmHg and 70% selected 95 mmHg. An additional 18% of the GDG agreed there should be no upper limit for MAP. Based on the voting, an upper limit of 90-95 mmHg was selected. While the evidence surrounding the upper limit was limited, the GDG agreed that the neurologic benefit of increasing MAP above 90-95 mmHg was questionable, and that the risk of complications from vasopressor or inotrope use was likely increased. Additional risks associated with MAP augmentation include risks associated with central line placement and utilization and risks from vasopressors including arrhythmia and ischemia.

For Peer Review

An important point of clarification is that this upper MAP limit of 90-95 only applies to patients who require active MAP augmentation (e.g. through volume resuscitation and the use of vasopressors and inotropes) and not to patients who achieve a MAP above 95 mmHg without medical intervention. To be clear, the GDG is not suggesting that treating MAP above 90-95 with anti-hypertensives, but rather avoiding medical augmentation of MAP above 90-95. For example, if an acute SCI patient naturally maintains (without any vasopressors) a blood pressure of 125/80 (MAP 95), this guideline does not recommend that treatment is needed to actively bring down the MAP; it is not. Rather, if vasopressors are being applied to artificially augment MAP, the GDG felt that this vasopressor support should not exceed the upper MAP limit of 90-95 mmHg.

For Peer Review

Clinical Practice Guideline Recommendation for the MAP Target

Population: Adult patients with acute SCI.

Key question: Should we recommend the augmentation of mean arterial blood pressure to at least 75-80 mmHg and not higher than 90-95 mmHg in order to optimize spinal cord perfusion in acute traumatic SCI?

Recommendation 1: We suggest the augmentation of mean arterial blood pressure to at least 75-80 mmHg but not higher than 90-95 mmHg in order to optimize spinal cord perfusion in acute traumatic SCI.

Quality of Evidence: Very Low

Strength of Recommendation: Weak

Evidence Summary for MAP Target Recommendation

We conducted a systematic review of the literature to inform this recommendation and the GRADE approach was used to rate the overall quality of evidence.[19] Based on very low quality evidence, the effect of MAP support on neurological recovery is uncertain. This uncertainty stems from the fact that no studies directly compared the effects of implementing various MAP targets on patient reported or neurologic outcomes. Fourteen studies, however, were identified that discussed the

association between neurologic recovery at varying time points post-injury and maintaining specific MAP goals; the results of these individual studies are provided above. With respect to adverse events, very low evidence suggested that the use of vasopressors or inotropes for MAP support may be associated with increased rates of cardiac arrhythmias, myocardial injury, acidosis, skin necrosis and other complications. However, one of the largest studies failed to identify an association between vasopressor use and rates of adverse events. Unfortunately, given the heterogeneity of the studies included in the systematic review, the GRADE approach was applied to the entire body of literature and not separately to the studies that focused on lower or upper limits of MAPs.

Rationale for MAP Target Recommendation

The outcomes ranked as critical for decision-making were change in neurologic function as reflected by improvement in AIS grade or ASIA motor score and adverse events including cardiac arrhythmias, myocardial infarction, hypertensive hemorrhage, stroke and skin necrosis. The strength of the evidence related to these outcomes was rated as "very low"; studies were downgraded for risk of bias, inconsistency and indirectness of evidence. The majority of the GDG (88%) agreed that the overall certainty of evidence was very low, with 12% of participants voting low.

The GDG agreed (88%) that there was probably no important uncertainty regarding how much key stakeholders value the main outcomes. Based on professional opinion, it is likely that clinicians and patients would similarly value the outcomes related to neurologic improvement and adverse events. While these outcomes would also be valued by payers, this stakeholder group would undoubtedly be interested in the impact of the recommendation on economic considerations such as hospital length of stay, cost and other administrative outcomes. Of note, 12% of the GDG suggested that there is possibly important uncertainty with respect to how much individuals living with SCI value the main outcomes, as values often differ among patients and because metrics of functional recovery, disability and quality of life may be more relevant to some than neurologic recovery as reflected by the ISNCSCI examination.

The anticipated desirable effects of augmenting MAP to between 75-80 mmHg and 90-95 mmHg include change in AIS grade and improvement in ASIA motor scores. Based on very low quality

For Peer Review

evidence, the anticipated desirable effects of MAP support on neurologic recovery remains uncertain. Given the limitations in the literature, 59% of the GDG voted that the anticipated desirable effects were small, 29% selected moderate and 12% indicated large. While the systematic review failed to demonstrate a clear association between MAP and neurologic recovery, the GDG acknowledged that results from several individual studies suggested that augmenting MAP can result in change in AIS by one or sometimes two grades as well as improvement in ASIA motor score. When voting, the GDG also agreed that any improvement in neurologic function has the potential to significantly impact the quality of life of an individual living with SCI. Given these considerations, the GDG decided that the anticipated desirable effects were not trivial as the evidence might suggest but rather were either small or moderate. Furthermore, it was acknowledged that the impact of MAP on neurologic recovery may be influenced by a number of other patient factors including AIS, concomitant traumatic injuries and underlying co-morbidities.

The anticipated undesirable effects include adverse events secondary to vasopressor or inotrope use such as death, cardiac arrhythmias, myocardial infarction, hypertensive hemorrhage, stroke and skin necrosis. Based on very low evidence, the use of vasopressors or inotropes for MAP support may be associated with increased rates of complications, especially cardiovascular. Ninety-four percent of the GDG agreed that the anticipated undesirable effects were small given that the majority of cardiovascular complications were related to changes in heart rate, such as bradycardia or tachycardia, and were not life threatening. Given that targeting MAP between 75-80 mmHg and 90-95 mmHg may yield small to moderate neurologic improvement, and because the adverse events associated with vasopressor or inotrope use are small, 78% of the GDG agreed that the balance between desirable and undesirable effects probably favor the intervention.

In the absence of high-quality evidence in the literature, the GDG used their clinical expertise to discuss the resources required to target a MAP between 75-80 mmHg and 90-95 mmHg in patients with SCI. Unfortunately, there are no available studies that summarize the resources required to achieve this or any other proposed MAP goal. However, given that patients often require vasopressors or inotropes to reach a MAP target of at least 75-80 mmHg, and because they must be monitored continuously in an ICU setting, the majority of the GDG agreed that the costs associated with this recommendation are either moderate (78%) or large (6%). An additional 11% of participants stated that they did not know how large the resource requirement was to maintain

For Peer Review

this MAP target. The GDG unanimously agreed that there were no included studies that compare resource requirements between patients who achieve the MAP goal and those who do not. With respect to the cost-effectiveness of MAP augmentation therapy (to a goal 75-80 mmHg and 90-95 mmHg), 80% of the GDG were uncertain whether the incremental cost associated with ICU monitoring and vasopressor or inotrope use was small relative to the benefit of maintaining MAP within this target range. In contrast, 20% of the GDG rationalized that if augmenting MAP improves neurologic recovery then this intervention may result in significant lifelong savings and is therefore probably cost-effective.

The GDG agreed (95%) that a recommendation for a MAP goal between 75-80 mmHg and 90-95 mmHg in patients with traumatic SCI would probably reduce health inequities if standardized pathways of care were implemented to ensure that patients with hemodynamic instability were monitored closely in an ICU setting with purposeful augmentation of their MAP to a specific target. The GDG acknowledged that if policy makers fund initiatives to ensure that these patients are triaged appropriately and have better access to higher level care, then there would be less disparity across socioeconomic groups and geographic regions. Practically speaking, however, the cost associated with the level of care required to achieve this MAP target may be prohibitively expensive in certain developing nations, such that health inequities may be increased (5% of GDG voted with this sentiment). The GDG (94%) agreed that the proposed MAP target would probably be acceptable to key stakeholders given the potential neurologic benefit and the small risks associated with medical augmentation of MAP (e.g. risk of central line placement, and risk of cardiac arrhythmia and ischemia with vasopressors). During discussions among members of the GDG, it was emphasized that patients with devastating injuries may value small neurologic improvement as these may translate to clinically meaningful changes in functional status and quality of life. The majority of the GDG voted "probably yes" instead of "yes" with respect to acceptability of this recommendation due to limited data on the resource requirement and costs associated with prolonged ICU monitoring. Finally, there was no consensus within the GDG with respect to whether the target MAP goals were feasible to implement. Based on the voting, 28% were uncertain, 50% voted probably yes, 6% selected yes and 17% stated that it varies. This lack of consensus reflects that while this MAP goal could be readily adopted into clinical practice at some institutions, it may not be feasible to implement in others, particularly low to middle income countries.

For Peer Review

Finally, considering all of these factors, 89% of the GDG voted that the desirable consequences of maintaining MAP between 75-80 mmHg and 90-95 mmHg outweighed undesirable consequences in most setting. This consensus led to the formation of a weak recommendation (84% “suggested” offering this option, while 16% “recommended” offering this option).

The final wording of this recommendation for maintaining a target MAP between 75-80 mmHg and 90-95 mmHg was subjected to a final vote of all the GDG members and consensus was achieved for its approval (>85%). There was concern raised in this final voting that it be clarified that while we have recommended an upper limit of 90-95 mmHg for the MAP target, we do not believe that clinicians should actively reduce the MAP in patients who were physiologically maintaining a MAP higher than 90-95 mmHg on their own. In other words, when actively trying to augment the MAP with vasopressors, the recommended upper limit is 90-95 mmHg; however, in a patient whose MAP is above 95 mmHg without any volume resuscitation or vasopressors, we do not recommend actively trying to lower the MAP as part of this hemodynamic management protocol. Also, there remained concern about the lower limit of 75-80 mmHg being too low, and it was acknowledged that the evidence around the limits remains weak and clinicians will be obviously able to use their clinical judgment for achieving a target within the proposed range.

PART 2. DURATION OF MAP AUGMENTATION

The second part of this guideline aims to address the following key question: *Should we recommend the augmentation of mean arterial blood pressure for a duration of Z days in order to optimize spinal cord perfusion in acute traumatic spinal cord injury?* The following section will summarize how the duration “Z” was decided.

Defining the Duration of MAP Augmentation

In addition to identifying the MAP range to target in acute SCI patients, another practical consideration for hemodynamic management is the duration of MAP augmentation. The 2013 AANS/CNS guidelines recommended that a MAP target of 85-90 mmHg be maintained for 7 days. The duration of aggressive hemodynamic management is an important consideration given that access to an ICU bed for monitoring and treatment may be unavailable in low and middle-income

For Peer Review

nations, and limited even in wealthier countries with well-organized infrastructure. Furthermore, in settings where ICU or high-acuity beds are available, there is usually pressure to move patients out of these units, making it difficult to adhere to the recommended 7 days. Furthermore, the previous guideline did not distinguish between patients with different severities of neurologic impairment. For the purpose of this revised guideline, we searched our systematic review in order to collect evidence that would support a specific duration of MAP-targeted treatment.

Fourteen studies were identified within the systematic review that discussed the duration of hemodynamic management (**Table 4**). Of note, there was considerable variation in the duration of monitoring across studies, hindering efforts to effectively synthesize the results. Furthermore, there was a paucity of studies that compared the effects of varying durations of MAP therapy on neurologic recovery or other outcomes. At one extreme, Ehsanian et al. examined MAP only during the surgical procedure.[33] Although maintaining an intraoperative MAP between 70 mmHg and 94 mmHg was positively associated with motor recovery, further conclusions with respect to the optimal number of days of MAP management cannot be gleaned from this study. Similarly, a study by Sewell et al. only evaluated episodes of hypotension within the first 24 hours and was unable to identify an association with neurologic improvement.[37] The remaining studies assessed durations of MAP monitoring from 3 to 7 days. Studies by Catapano et al., Martin et al., and Weinberg et al., monitored MAP over a 3-day period and either concluded that MAP augmentation to a target >85 mmHg was associated with neurologic improvement,[14,29] or that it did not influence outcomes.[34] Three other studies monitored and managed MAP for a 5-day period.[6,12,28] Hawryluk et al. reported that higher MAPs correlated with neurological outcomes in the first 3 days, and that patients who improved by 2 or more AIS grades had a reduced number of episodes below 85 mmHg, especially in the first 24 hours.[12] This study also noted that while there was a difference in the frequency of MAP recordings below 85 mmHg between patients who improved and those who did not, this difference seemed to diminish with time. This result suggests that timely support of MAP early within the first week post-injury is potentially more important from a neuroprotection standpoint than treatment later in the week. A study by Dakson et al. concluded that patients whose MAP dropped below 85 mmHg for 2 hours within the first 5 days fared worse neurologically than those who consistently maintained this MAP goal.[28] Finally, although Squair et al. reported that a MAP < 70 mmHg was associated with an increased risk of poor neurologic recovery, outcomes based on distinct durations of relative hypotension were not described.[6] In an

For Peer Review

additional analysis, exposure to low SCPP in the first 36 hours had the most impact on neurologic outcomes, suggesting that supporting perfusion early in the first week post-injury is the most critical.

Five studies monitored and managed MAP over a 7 day period and reported variable impact on neurologic recovery.[15,30-32,36] In the study by Cohn et al, the percent of time patients spent with a MAP below 65 or 70 mmHg during a 7 day period was inversely correlated with AIS grade improvement.[31] In the study of AIS C patients, Hogg et al. did not standardize duration of treatment but did demonstrate that improvement in motor function was more closely related to SCPP during a 7 day period.[32] Similarly, Saadoun et al. concluded that neurologic improvement was optimized when SCPP was maintained over 90 mmHg for 7 days.[15] In the study by Park et al., a patient's MAP on arrival to the hospital was significantly associated with outcome, whereas the average MAP over a 7 day period did not correlate with change in AIS grade.[36] Unfortunately, these studies did not explore neurologic outcomes at different time points during the first week of injury but are often interpreted as supporting 7 days as the ideal period for hemodynamic management.

Finally, Haldrup et al. targeted a MAP over 80 mmHg and examined different time periods, including pre-hospital transport, intra-operatively, and days 1-2 and 3-7 of hospitalization.[30] According to their results, a significant relationship between MAP and neurologic outcome was evident in the first two days post-injury but less prominent on days 3-7 of hospitalization. However, the figure included in this paper demonstrated no apparent differences in the median MAPs among patients who improved, were unchanged, or worsened at days 1-2 or days 3-7. Therefore, although this study suggested that supporting MAP during the first two days following injury is more important than later on, our confidence in these conclusions is limited given the data presented in the paper.

Ultimately, the literature included in our updated systematic review did not provide strong guidance with respect to the optimal duration of hemodynamic management. Of note, high quality evidence was also not available when the previous guidelines recommended targeting MAP goals for 7 days. Instead, this time frame was largely adopted from the original Levi et al.[2] study which aimed to keep MAP > 90 mmHg for up to 7 days, and the Vale et al.[3] study which advocated for

For Peer Review

maintaining MAP >85 mmHg for 7 days in an ICU setting. In the discussion of the Vale et al. study, the authors indicated that they selected 7 days as the minimum duration for treatment *"based on data from experimental SCI studies which indicate that maximum cord edema and spinal cord vascular congestion occur between 3 and 5 days after spinal cord injury". The patients were weaned from therapy earlier than 7 days post-injury if they maintained a MAP greater than 85 mmHg without administration of vasopressors, and if they were medically stable with respect to any associated injury".*[3] And so, even though the Vale et al. article is highly cited for substantiating the 7-day period of hemodynamic management, the authors themselves indicated that they shortened this time window in certain circumstances.

While some evidence in our systematic review suggested that supporting MAP during the first few days post-injury is the most important, other studies concluded that hemodynamic management significantly affects neurologic outcomes when sustained over a 7-day period. Furthermore, the literature does not clearly address the spectrum of injury severity and how a spinal cord that is injured in a high-energy fracture dislocation may have different hemodynamic requirements than a spinal cord injured following a low-energy impact without spinal column fracture. This heterogeneity makes it conceptually difficult to justify a 7-day window of hemodynamic treatment for all SCI patients.

GDG Voting on the Duration of MAP Augmentation

Following the presentation of the results of these fourteen studies, the GDG voted on the ideal duration of MAP-targeted treatment. Fifty percent of participants voted for 5 days, 30% suggested 3 days, 10% selected 7 days and 5% proposed 1-2 days as the duration of hemodynamic management. In addition, 5% of the GDG specified no duration. Given the variability in responses, the GDG recommended a range of treatment durations between 3 and 7 days following injury, leaving it at the discretion of the clinician to decide a specific duration within this range. In voting for this range, the GDG acknowledged that while the first few days post-injury are perhaps the most critical, there may be circumstances where 7 days of hemodynamic treatment is warranted. As such, a range for the duration of treatment allows the managing physician to adopt a more personalized approach based on injury severity and a patient's need for aggressive and prolonged

For Peer Review

MAP augmentation. Eighty-two percent of the GDG agreed to develop a recommendation based on the range of 3 to 7 days.

Clinical Practice Guideline Recommendation for Duration of MAP Augmentation

Population: Adult patients with acute spinal cord injury.

Key question: Should we recommend the augmentation of mean arterial blood pressure for a duration of 3-7 days in order to optimize spinal cord perfusion in acute traumatic SCI?

Recommendation 1: We suggest the augmentation of mean arterial pressure for a duration of 3-7 days in order to optimize spinal cord perfusion in acute traumatic spinal cord injury.

Quality of Evidence: Very Low

Strength of Recommendation: Weak

For Peer Review

Evidence Summary for Duration of MAP Augmentation

A systematic review of the literature was conducted to inform the guideline process and to determine the effects of specific durations of MAP-targeted therapy on the extent of neurologic recovery and rates of adverse events. Based on this review, no study was identified that directly compared the effects of varying durations of MAP support on neurologic recovery, patient-reported outcomes or adverse events. Using GRADE, the overall quality of evidence was rated as very low due to risk of bias, indirectness and imprecision. Fourteen studies, however, were identified that reported on the association between neurologic outcomes and maintaining MAP or SCPP goals for 3 to 7 days following injury; the results of these individual studies are summarized above.

Rationale for Recommendation on MAP Augmentation Duration

The outcomes ranked as critical for decision making were identical to part 1 and included change in neurological function as reflected by improvement in AIS grade or ASIA motor score and adverse events. The strength of the evidence related to these outcomes was rated as very low. Ninety-five percent of the GDG agreed that the overall certainty of evidence was very low. Similar

to part 1, 89% of the GDG agreed that there was probably no important uncertainty regarding how much key stakeholders value the main outcomes.

The anticipated desirable effects of maintaining MAP goals for 3 to 7 days include change in AIS grade and improvement in ASIA motor score. Based on very low evidence, the anticipated desirable effects of our proposed duration of MAP support on neurologic recovery were uncertain. The GDG, however, acknowledged that several studies supported early and timely intervention of MAP augmentation in order to optimize outcomes, while others demonstrated a significant association between neurologic recovery and MAP therapy continued for 7 days. The GDG agreed that the anticipated desirable effects were small, with 15% of participants voting that the desirable effects were moderate. The anticipated undesirable effects include adverse events associated with prolonged vasopressor or inotrope use such as death, cardiac arrhythmias, myocardial infarction, hypertensive hemorrhage, stroke and skin necrosis. Based on very low evidence, the anticipated undesirable effects of maintaining MAP goals for 3 to 7 days were uncertain, as no study was identified that compared rates of complications based on duration of MAP augmentation. Based on professional opinion, however, the GDG agreed that prolonged use of vasopressors or inotropes is likely associated with an increased risk of adverse events. The majority of the GDG (94%) voted that the anticipated undesirable effects were small given that the majority of cardiovascular complications were related to changes in heart rate and were not life threatening. Given that MAP-targeted therapy for a duration of 3 to 7 days may result in neurological improvement and because the undesirable effects were deemed to be small, 95% of the GDG agreed that the balance between the desirable and undesirable effects probably favored the intervention.

For Peer Review

In the absence of literature, the GDG used their clinical expertise to discuss the resources required to target MAP goals for 3 to 7 days. Unfortunately, there were no available studies that summarized the resources required to maintain MAP for this or any duration. However, the GDG acknowledged that the resource requirements are increased the longer a patient needs to be on MAP-directed therapy, monitored continuously in an ICU setting, and on vasopressors or inotropes. The majority of the GDG agreed that the costs associated with this recommendation were either moderate (83%) or large (6%). Eighty-four percent of GDG agreed that there were no included studies that compared the resource requirement between patients who receive MAP-directed therapy for 3 to 7 days and those who do not. With respect to the cost-effectiveness of this recommendation, 79%

of the GDG stated that they did not know whether the incremental cost associated with ICU monitoring and vasopressor or inotrope use for 3 to 7 days was small relative to the benefit of maintaining MAP targets for this duration. In contrast, 21% of the GDG suggested that if augmenting MAP improves neurologic recovery than this intervention may result in significant lifelong savings and is therefore probably cost-effective. Similar to part 1, 89% of the GDG agreed that implementation of this recommendation would probably reduce health inequities.

Sixty-one percent of the GDG agreed that the proposed duration of MAP-targeted therapy would probably be acceptable to key stakeholders, while 39% were uncertain given the current state of the literature. Participants expressed uncertainty with respect to whether this recommendation would be acceptable to key stakeholders given the available evidence related to benefit, the risks associated with prolonged vasopressor or inotrope use and the increased costs required for extended monitoring in an ICU setting. Finally, there was no consensus within the GDG as to whether the duration of MAP treatment were feasible to implement. Based on the voting, 39% were uncertain, 56% voted probably yes, and 6% selected yes. This lack of consensus reflects that while supporting MAP for 3 to 7 days could be achieved at some institutions, it may not be feasible to implement at others, such as in resource-limited settings.

For Peer Review

Finally, considering all of these factors, 89% of the GDG voted that the desirable consequences of maintaining MAP goals for 3 to 7 days outweigh undesirable consequences in most settings. Based on this vote, 84% "suggested" offering this option, while 16% "recommended" offering this option.

The final wording of this recommendation for maintaining a target MAP for 3-7 days was subjected to a final vote of all the GDG members and consensus was achieved for its approval (>90%). There was concern raised in this final voting that reducing the duration to 3 days would be "abused" as a rationalization to discontinue aggressive hemodynamic management in some patients who might otherwise benefit from it. Here, clinical judgement will be required to strike a delicate balance between the potential benefit of prolonging MAP augmentation therapy and the ongoing resource utilization associated with extending ICU stay.

PART 3. CHOICE OF VASOPRESSORS OR INOTROPES FOR MAP AUGMENTATION

The third part of this guideline aims to address the following key question: *Should we recommend the use of a specific vasopressor or inotrope in order to achieve MAP-directed goals in patients with acute traumatic SCI?*

If the MAP is to be elevated to a certain target for acute SCI patients, then the interventions used to achieve this goal become clinically important. Again, we reviewed the studies included in the systematic review to determine if the literature would support the use of a specific vasopressor or inotrope for MAP augmentation. Three studies published by the UCSF group were identified that largely focused on rates of complications and adverse events with the use of different vasopressors or inotropes.[42-44] (**Table 5**) In a study by Inoue et al., cardiovascular complications frequently occurred with the use of vasopressors (e.g. cardiac arrhythmias) and were more common with dopamine compared to phenylephrine.[42] Furthermore, the increased risk of complications with dopamine was particularly remarkable in older patients. These findings were reproduced in two papers published by Readdy et al., which either examined patients with central cord syndrome (again reporting a slightly higher incidence of complications with dopamine)[44] or penetrating SCI.[43]

Unfortunately, while these studies suggested an increased risk of complications with the use of dopamine (particularly in the elderly), none addressed which, if any, pharmacologic agent is better at promoting blood flow and oxygenation to the injured spinal cord. Additionally, while many clinicians use norepinephrine to support MAP, there were no studies within our systematic review that evaluated the safety or effectiveness of this vasopressor in patients with SCI. As such, the use of norepinephrine in this patient population remains an important knowledge gap. Kwon and colleagues conducted an experiment within a clinical trial of CSF pressure monitoring where acute SCI patients were "crossed-over" between dopamine and norepinephrine to evaluate the effect of these medications on intrathecal CSF pressure and thus SCPP.[45] Somewhat surprisingly, the use of dopamine resulted in a MAP-independent increase in intrathecal pressure, thus reducing SCPP. This data suggests that if the choice between these two drugs was based solely on clinician preference (i.e. equipoise for the clinician), then there might be an advantage from an SCPP standpoint to use norepinephrine over dopamine.

For Peer Review

Given that specific vasopressors and inotropes have different affinities to adrenergic receptors, they might have different effects on spinal cord vasculature within the injured spinal cord. Until methods are developed to assess this hypothesis *in vivo* and ultimately in human SCI, such comparative data remains unavailable. In a pig model of SCI, Streijger et al. compared invasive, intra-parenchymal measures of oxygenation and blood flow during and after spinal cord compression, and determined that norepinephrine was better at restoring perfusion than phenylephrine.[46] Furthermore, Williams et al. demonstrated that dobutamine is better than norepinephrine in this context at restoring cardiac output (which is affected immediately after injury) and improving oxygenation in the spinal cord, suggesting that a cardio-centric approach to hemodynamic management may be required in certain patients instead of just using vasopressors that affect peripheral vascular resistance.[47]

Members of GDG were asked to vote on whether the choice of vasopressor or inotrope should be left at the discretion of the attending physician or whether a specific recommendation should be made. Given the limitations in the literature, 89% of the GDG agreed that this decision should be made by the treating physician. Furthermore, the GDG acknowledged that because the choice of vasopressor or inotrope is dependent on several patient factors, including the presence of a central line, concurrent cardiopulmonary dysfunction and co-morbidities, a recommendation for a specific drug would be too restrictive.

SPINAL CORD PERFUSION PRESSURE AND HEMODYNAMIC MANAGEMENT

One of the most interesting areas of investigation since the 2013 AANS/CNS guidelines is the recognition that SCPP may be a more relevant metric than MAP for neurologic recovery. This work has been largely pioneered by Papadopoulos and colleagues, who developed a pressure sensor that is inserted at the injury site in order to detect swelling of the spinal cord against the dura. A series of studies from the observational clinical trial "ISCOPE" were included in our systematic review and demonstrated that neurologic recovery may be more closely tied to the SCPP than it is to the MAP.[15,32] Importantly, by evaluating spinal cord autoregulation with pressure reactivity indices, Papadopoulos and colleagues have suggested that the 'optimal SCPP' (i.e the SCPP at which autoregulation is best maintained) can differ quite significantly among patients. [15] The work of Kwon and colleagues is also centered around SCPP but with the use of

For Peer Review

lumbar intrathecal catheters instead of pressure catheters at the injury site. In the study by Squair et al.[6] the SCPP appeared to be a more relevant measure with respect to patient outcomes than MAP, supporting the work of Papadopoulos and colleagues.

These findings raise the question about the value in recommending a generic MAP target for acute SCI patients if the "optimal SCPP" (and thus the optimal MAP) may differ among patients, and because MAP may not be as closely related to neurologic recovery as SCPP. At this stage, however, the GDG agreed that recommending a MAP target was useful for a number of reasons. First, measurement of SCPP either by placing a pressure sensor at the injury site[8] or in the lumber cistern[6] is not widely performed and the vast majority of clinicians still manage their patients based on MAP. Second, the SCPP may differ if measured by a pressure monitor at the injury site versus a pressure monitor at the lumbar cistern if there is occlusion of the subarachnoid space due to either extradural compression or intradural compression from the swollen spinal cord. This fact was demonstrated in a study by Hogg et al. where pressure catheters were placed at both the injury site and the lumbar cistern.[48] Based on their results, when the subarachnoid space is closed at the injury site, the intrathecal pressure in the lumbar cistern is likely to be lower than the pressure at the injury site where the spinal cord may be swollen and confined by the relatively inelastic dura. This further emphasizes the importance of the surgical technique used for decompression of the spinal cord. Specifically, Aarabi and colleagues demonstrated that a long posterior laminectomy in the vast majority (>90%) of cases can restore the subarachnoid space around the injury site.[49-51] Nonetheless, given that these techniques are only done at select centers and because the quality of evidence remains low, the GDG agreed that it would not be meaningful to generate a recommendation around SCPP monitoring or management. This is a growing area of interest and future research is required to determine how to best monitor SCPP and assess its association with neurologic recovery. It is anticipated that the results of an ongoing clinical trial that uses CSF drainage to improve SCPP ("CASPER"; Clinicaltrials.gov NCT03911492) will provide further insight on this topic and enhance the current body of literature. As new evidence emerges, a recommendation on monitoring and managing SCPP may be made in the future.

EVIDENCE GAPS AND FUTURE RESEARCH RECOMMENDATIONS

For Peer Review

This guideline was undertaken as new research has emerged since the publication of the 2013 AANS/CNS guidelines and because the recommendations surrounding MAP warranted clarification given the very narrow proposed target range (85 to 90 mmHg) and the variable adaptations in the literature. Unfortunately, the quality of evidence that addresses the main question of *"what should the MAP target be?"* continues to be low, preventing firm conclusions about the neurologic benefit of maintaining specific MAP goal. Accepting these limitations in the literature, the GDG agreed that the guidelines still warranted reconsideration due to the proposed narrow target range of 85-90 mmHg. For one, recommending a 5-mmHg range implies that neurologic improvement is tied to MAPs of 85, 86, 87, 88, 89 and 90mmHg (which does not appear to be the case) and that such a target can be feasibly maintained (which it cannot). Furthermore, studies by Squair et al. and Martin et al. demonstrated that neurologic improvement can occur when the MAP is maintained at much lower levels than 85 mmHg (e.g. 70 mmHg).[6,34] While it was agreed that hypotension should be avoided, the GDG proposed a lower limit target of 75-80 mmHg given that some studies identified no association between lower MAPs and neurologic outcome.

On the other end, the GDG agreed that an upper limit should be proposed given that some centers have interpreted the previous guidelines as maintaining the MAP above 85 mmHg and because some studies have revealed that neurologic outcomes do not improve, or may even worsen, beyond a certain MAP. Furthermore, other studies have identified complications associated with the use of vasopressors or inotropes and have recognized that augmenting the MAP with these pharmacological therapies is not totally benign. As such, if there exists a target beyond which neurologic improvement is not promoted, then further increasing the MAP with vasopressors or inotropes would only subject the patient to unwarranted risks. The caveat here is that some patients might actually autoregulate best at exceedingly high MAPs (over 100).[8] However, given that other studies demonstrated less neurologic benefit with MAPs above 95mmHg, the GDG proposed an upper limit target of 90-95 mmHg.

One of the questions that will undoubtedly arise from this guideline is *"If the previous recommendations of targeting MAP between 85-90 mmHg was problematic because it is practically impossible to maintain MAP within a 5 mmHg range, then why would we implement a 5 mmHg lower limit (75-80 mmHg) and a 5 mmHg upper limit (90-95mmHg)? Why not just set a*

For Peer Review

lower limit of 75 mmHg and an upper limit of 95 mmHg?" It was the objective of the GDG to provide an achievable MAP target by recognizing that there are minute-to-minute variations in blood pressure in the SCI population. The GDG agreed that if the lower limit was set at 75 mmHg, then there would invariably be times when the MAP dropped below this threshold, potentially negatively impacting neurologic outcomes. Instead, by recommending a lower limit between 75 and 80 mmHg, it is anticipated that there will be fewer instances when MAP drops below 75 mmHg given that clinicians will aim at or above this 5-mmHg range. Similarly, we proposed a 5-mmHg range between 90-95 mmHg for the upper limit target of MAP. There is some evidence that suggests augmenting MAP beyond 95 mmHg may result in worse neurologic recovery (or at least no further improvement) and increased rates of complications associated with vasopressor or inotrope use, making this threshold a reasonable "upper limit". Again, if clinicians targeted a MAP below 95 mmHg, there would be times when the MAP surpassed this threshold, resulting in unnecessary risks to the patient without neurologic benefit. Instead, by suggesting an upper limit between 90 and 95 mmHg, clinicians may be more cautious when approaching this target such that patients would be less likely to experience MAPs above 95 mmHg. By proposing a range for the lower and upper limit, the GDG agreed that this would result in fewer episodes of MAP below 75 mmHg and above 95 mmHg. Furthermore, these ranges embrace the realities of hemodynamic management in patients with SCI – a population whose pressures often fluctuate and where precise control does not exist.

The systematic review of the literature and the guideline development process exposed important knowledge gaps with respect to the hemodynamic management of SCI and opportunities for future research. Further high-quality prospective studies, with creative designs, are warranted in order to evaluate the relationship between MAP and neurologic outcomes. While a prospective randomized trial comparing specific MAP targets would significantly contribute to the literature, conducting such a study is almost impossible considering the history of interventional trials in acute SCI that have attempted to compare one therapeutic approach against another in a controlled manner. Furthermore, the question of which vasopressor to use to manage hemodynamics in SCI patients remains a significant knowledge gap. In this regard, it is conceivable that applying machine learning approaches to a large database can help to establish the adverse event profile of different vasopressors used in patients with acute SCI. It will, however, be challenging to establish appropriate methodology for determining the differential effects of these vasopressors on the

For Peer Review

injury penumbra over time. Finally, Saadoun and Papadopoulos asked in their article *"Is monitoring from the injury site the future?"*[8] However one responds to this question, injury site monitoring is clearly a knowledge gap that needs to be addressed. In some respects, the injury site that we are treating by managing hemodynamics remains a "black box" and the development of an easy, widely available way to monitor this site over time is necessary in order to guide treatment. Currently, Kwon and colleagues are working on an epidural near-infrared spectroscopy (NIRS) biosensor for the spinal cord that is intended to monitor tissue oxygen levels; however, this approach remains experimental and requires further investigation.[52,53]

IMPLEMENTATION CONSIDERATIONS

Dissemination of this guideline will be accomplished at multiple levels in order to ensure effective implementation into clinical practice:

- Presentation at international spine surgery, critical care, neurology, and anesthesiology conferences.
- Scientific and educational courses.
- Online webinars that engage a broad audience in an interactive format.
- Publication of a focus issue in the Global Spine Journal.

INTERNAL APPRAISAL AND EXTERNAL REVIEW

The leader of the GDG and a methodologist from Aggregate Analytics completed an internal appraisal of the final guideline using the AGREE II checklist. A multidisciplinary group of stakeholders (see Appendix) were invited to externally review this guideline document prior to publication. This guideline was also reviewed by several societies (see Appendix). The methods paper published elsewhere in this focus issue summarizes additional details of these processes and highlights the conflicts of interest of both internal and external reviews.

PLANS FOR UPDATING

This guideline with be reviewed by AO Spine, Praxis Spinal Cord Institute and members of the leadership group at three to five years following publication. The guideline will be updated at this time, or earlier, if there are changes in (i) the evidence related to benefits and harms, (ii) outcomes

For Peer Review

deemed critical for decision-making; (iii) knowledge related to resource requirements and cost-effectiveness; and (iv) available interventions and resources.

For Peer Review

REFERENCES

1. Ryken TC, Hurlbert RJ, Hadley MN, et al. The acute cardiopulmonary management of patients with cervical spinal cord injuries. *Neurosurgery*. Mar 2013;72 Suppl 2:84-92. doi:10.1227/NEU.0b013e318276ee16

2. Levi L, Wolf A, Belzberg H. Hemodynamic parameters in patients with acute cervical cord trauma: description, intervention, and prediction of outcome. *Neurosurgery*. Dec 1993;33(6):1007-16; discussion 1016-7.

3. Vale FL, Burns J, Jackson AB, Hadley MN. Combined medical and surgical treatment after acute spinal cord injury: results of a prospective pilot study to assess the merits of aggressive medical resuscitation and blood pressure management. *J Neurosurg*. Aug 1997;87(2):239-46. doi:10.3171/jns.1997.87.2.0239

4. *Early Acute Management in Adults with Spinal Cord Injury: A Clinical Practice Guideline for Health-Care Professionals*. 2008:20-21.

5. Squair JW, Bélanger LM, Tsang A, et al. Empirical targets for acute hemodynamic management of individuals with spinal cord injury. *Neurology*. Sep 17 2019;93(12):e1205-e1211. doi:10.1212/wnl.0000000000008125

6. Squair JW, Bélanger LM, Tsang A, et al. Spinal cord perfusion pressure predicts neurologic recovery in acute spinal cord injury. *Neurology*. Oct 17 2017;89(16):1660-1667. doi:10.1212/wnl.0000000000004519

7. Varsos GV, Werndle MC, Czosnyka ZH, et al. Intraspinal pressure and spinal cord perfusion pressure after spinal cord injury: an observational study. *Journal of neurosurgery Spine*. Aug 14 2015:1-9. doi:10.3171/2015.3.SPINE14870

8. Saadoun S, Papadopoulos MC. Spinal cord injury: is monitoring from the injury site the future? *Critical care*. Oct 05 2016;20(1):308. doi:10.1186/s13054-016-1490-3

9. Yang J, Skaggs DL, Chan P, et al. Raising Mean Arterial Pressure Alone Restores 20% of Intraoperative Neuromonitoring Losses. *Spine (Phila Pa 1976)*. Jul 1 2018;43(13):890-894. doi:10.1097/brs.0000000000002461

10. Panthee N, Ono M. Spinal cord injury following thoracic and thoracoabdominal aortic repairs. *Asian Cardiovasc Thorac Ann*. Feb 2015;23(2):235-46. doi:10.1177/0218492314548901

11. Khan NR, Smalley Z, Nesvick CL, Lee SL, II LMM. The use of lumbar drains in preventing spinal cord injury following thoracoabdominal aortic aneurysm repair: an updated systematic review and meta-analysis. *Journal of Neurosurgery: Spine*. 2016;25(3):383-393. doi:10.3171/2016.1.Spine151199

12. Hawryluk G, Whetstone W, Saigal R, et al. Mean Arterial Blood Pressure Correlates with Neurological Recovery after Human Spinal Cord Injury: Analysis of High Frequency Physiologic Data. *J Neurotrauma*. Dec 15 2015;32(24):1958-67. doi:10.1089/neu.2014.3778

For Peer Review

13. Gee CM, Tsang A, Bélanger LM, et al. All over the MAP: describing pressure variability in acute spinal cord injury. *Spinal Cord*. May 2022;60(5):470-475. doi:10.1038/s41393-022-00802-0

14. Catapano JS, John Hawryluk GW, Whetstone W, et al. Higher Mean Arterial Pressure Values Correlate with Neurologic Improvement in Patients with Initially Complete Spinal Cord Injuries. *World Neurosurg*. Dec 2016;96:72-79. doi:10.1016/j.wneu.2016.08.053

15. Saadoun S, Chen S, Papadopoulos MC. Intraspinal pressure and spinal cord perfusion pressure predict neurological outcome after traumatic spinal cord injury. *J Neurol Neurosurg Psychiatry*. May 2017;88(5):452-453. doi:10.1136/jnnp-2016-314600

16. Kwon BK. AO Spine/Praxis Clinical Practice Guidelines for the Management of Acute Spinal Cord Injury: An Introduction to a Focus Issue (In Review). *Global Spine Journal*. 2023;

17. Shiffman RN, Michel G. Toward improved guideline quality: using the COGS statement with GEM. *Stud Health Technol Inform*. 2004;107(Pt 1):159-63.

18. Shiffman RN, Shekelle P, Overhage JM, Slutsky J, Grimshaw J, Deshpande AM. Standardized reporting of clinical practice guidelines: a proposal from the Conference on Guideline Standardization. *Ann Intern Med*. Sep 16 2003;139(6):493-8.

19. Evaniew N. Interventions to Optimize Spinal Cord Perfusion in Patients with Acute Traumatic Spinal Cord Injury: An Updated Systematic Review (In Review). *Global Spine Journal*. 2023;

20. Moher D, Liberati A, Tetzlaff J, Altman DG. Preferred Reporting Items for Systematic Reviews and Meta-Analyses: The PRISMA Statement. *Journal of Clinical Epidemiology*. 2009/10/01/ 2009;62(10):1006-1012. doi:10.1016/j.jclinepi.2009.06.005

21. Institute of Medicine Committee on Standards for Developing Trustworthy Clinical Practice G. *Clinical Practice Guidelines We Can Trust*. National Academies Press (US); 2011.

22. Balshem H, Helfand M, Schünemann HJ, et al. GRADE guidelines: 3. Rating the quality of evidence. *J Clin Epidemiol*. Apr 2011;64(4):401-6. doi:10.1016/j.jclinepi.2010.07.015

23. Andrews J, Guyatt G, Oxman AD, et al. GRADE guidelines: 14. Going from evidence to recommendations: the significance and presentation of recommendations. *J Clin Epidemiol*. Jul 2013;66(7):719-25. doi:10.1016/j.jclinepi.2012.03.013

24. Andrews JC, Schunemann HJ, Oxman AD, et al. GRADE guidelines: 15. Going from evidence to recommendation-determinants of a recommendation's direction and strength. *J Clin Epidemiol*. Jul 2013;66(7):726-35. doi:10.1016/j.jclinepi.2013.02.003

25. Schunemann HJ, Oxman AD, Akl EA, et al. Moving from evidence to developing recommendations in guidelines: article 11 in Integrating and coordinating efforts in COPD guideline development. An official ATS/ERS workshop report. *Proceedings of the American Thoracic Society*. Dec 2012;9(5):282-92. doi:10.1513/pats.201208-064ST

26. Tetreault LM. AO Spine/Praxis Clinical Practice Guidelines for the Management of Acute Spinal Cord Injury: Overarching Development Process and Methodology (In Review). *Global Spine Journal*. 2023;

For Peer Review

27. Tetreault LM. Clinical Practice Guideline Protocol Summary for Timing of surgery, Hemodynamics, and Intraoperative SCI (In Review). *Global Spine Journal*. 2023;

28. Dakson A, Brandman D, Thibault-Halman G, Christie SD. Optimization of the mean arterial pressure and timing of surgical decompression in traumatic spinal cord injury: a retrospective study. *Spinal Cord*. Nov 2017;55(11):1033-1038. doi:10.1038/sc.2017.52

29. Weinberg JA, Farber SH, Kalamchi LD, et al. Mean arterial pressure maintenance following spinal cord injury: Does meeting the target matter? *J Trauma Acute Care Surg*. Jan 1 2021;90(1):97-106. doi:10.1097/ta.0000000000002953

30. Haldrup M, Dyrskog S, Thygesen MM, Kirkegaard H, Kasch H, Rasmussen MM. Initial blood pressure is important for long-term outcome after traumatic spinal cord injury. *J Neurosurg Spine*. Mar 20 2020:1-5. doi:10.3171/2020.1.Spine191005

31. Cohn JA, Wright J, McKenna SL, Bushnik T. Impact of mean arterial blood pressure during the first seven days post spinal cord injury. *Top Spinal Cord Inj Rehabil*. 2010;15(3):96-106.

32. Hogg FRA, Kearney S, Zoumprouli A, Papadopoulos MC, Saadoun S. Acute Spinal Cord Injury: Correlations and Causal Relations Between Intraspinal Pressure, Spinal Cord Perfusion Pressure, Lactate-to-Pyruvate Ratio, and Limb Power. *Neurocrit Care*. Feb 2021;34(1):121-129. doi:10.1007/s12028-020-00988-2

33. Ehsanian R, Haefeli J, Quach N, et al. Exploration of surgical blood pressure management and expected motor recovery in individuals with traumatic spinal cord injury. *Spinal Cord*. Mar 2020;58(3):377-386. doi:10.1038/s41393-019-0370-5

34. Martin ND, Kepler C, Zubair M, Sayadipour A, Cohen M, Weinstein M. Increased mean arterial pressure goals after spinal cord injury and functional outcome. *J Emerg Trauma Shock*. Apr-Jun 2015;8(2):94-8. doi:10.4103/0974-2700.155507

35. Chen S, Smielewski P, Czosnyka M, Papadopoulos MC, Saadoun S. Continuous Monitoring and Visualization of Optimum Spinal Cord Perfusion Pressure in Patients with Acute Cord Injury. *J Neurotrauma*. Nov 1 2017;34(21):2941-2949. doi:10.1089/neu.2017.4982

36. Park JH, Kim JH, Roh SW, Rhim SC, Jeon SR. Prognostic factor analysis after surgical decompression and stabilization for cervical spinal-cord injury. *Br J Neurosurg*. Apr 2017;31(2):194-198. doi:10.1080/02688697.2016.1247781

37. Sewell MD, Vachhani K, Hockings J, Chan J, Alrawi A, Williams R. A Hemodynamic Safety Checklist Can Improve Blood Pressure Monitoring in Patients with Acute Spinal Cord Injury. *World Neurosurg*. Aug 2019;128:e225-e230. doi:10.1016/j.wneu.2019.04.106

38. Chen S, Gallagher MJ, Papadopoulos MC, Saadoun S. Non-linear Dynamical Analysis of Intraspinal Pressure Signal Predicts Outcome After Spinal Cord Injury. *Front Neurol*. 2018;9:493. doi:10.3389/fneur.2018.00493

39. Hogg FRA, Kearney S, Gallagher MJ, Zoumprouli A, Papadopoulos MC, Saadoun S. Spinal Cord Perfusion Pressure Correlates with Anal Sphincter Function in a Cohort of

For Peer Review

Patients with Acute, Severe Traumatic Spinal Cord Injuries. *Neurocrit Care*. Dec 2021;35(3):794-805. doi:10.1007/s12028-021-01232-1

40. Phang I, Werndle MC, Saadoun S, et al. Expansion duroplasty improves intraspinal pressure, spinal cord perfusion pressure, and vascular pressure reactivity index in patients with traumatic spinal cord injury: injured spinal cord pressure evaluation study. *J Neurotrauma*. Jun 15 2015;32(12):865-74. doi:10.1089/neu.2014.3668

41. Saadoun S, Papadopoulos MC. Targeted Perfusion Therapy in Spinal Cord Trauma. *Neurotherapeutics*. Apr 2020;17(2):511-521. doi:10.1007/s13311-019-00820-6

42. Inoue T, Manley GT, Patel N, Whetstone WD. Medical and surgical management after spinal cord injury: vasopressor usage, early surgerys, and complications. *J Neurotrauma*. Feb 1 2014;31(3):284-91. doi:10.1089/neu.2013.3061

43. Readdy WJ, Saigal R, Whetstone WD, et al. Failure of Mean Arterial Pressure Goals to Improve Outcomes Following Penetrating Spinal Cord Injury. *Neurosurgery*. Nov 2016;79(5):708-714. doi:10.1227/neu.0000000000001249

44. Readdy WJ, Whetstone WD, Ferguson AR, et al. Complications and outcomes of vasopressor usage in acute traumatic central cord syndrome. *J Neurosurg Spine*. Nov 2015;23(5):574-580. doi:10.3171/2015.2.Spine14746

45. Altaf F, Griesdale DE, Belanger L, et al. The differential effects of norepinephrine and dopamine on cerebrospinal fluid pressure and spinal cord perfusion pressure after acute human spinal cord injury. *Spinal Cord*. Jan 2017;55(1):33-38. doi:10.1038/sc.2016.79

46. Streijger F, So K, Manouchehri N, et al. A Direct Comparison between Norepinephrine and Phenylephrine for Augmenting Spinal Cord Perfusion in a Porcine Model of Spinal Cord Injury. *J Neurotrauma*. Jun 15 2018;35(12):1345-1357. doi:10.1089/neu.2017.5285

47. Williams AM, Manouchehri N, Erskine E, et al. Cardio-centric hemodynamic management improves spinal cord oxygenation and mitigates hemorrhage in acute spinal cord injury. *Nat Commun*. Oct 15 2020;11(1):5209. doi:10.1038/s41467-020-18905-8

48. Hogg FRA, Gallagher MJ, Kearney S, Zoumprouli A, Papadopoulos MC, Saadoun S. Acute Spinal Cord Injury: Monitoring Lumbar Cerebrospinal Fluid Provides Limited Information about the Injury Site. *J Neurotrauma*. May 1 2020;37(9):1156-1164. doi:10.1089/neu.2019.6789

49. Aarabi B, Olexa J, Chryssikos T, et al. Extent of Spinal Cord Decompression in Motor Complete (American Spinal Injury Association Impairment Scale Grades A and B) Traumatic Spinal Cord Injury Patients: Post-Operative Magnetic Resonance Imaging Analysis of Standard Operative Approaches. *Journal of neurotrauma*. Mar 19 2019;36(6):862-876. doi:10.1089/neu.2018.5834

50. Chryssikos T, Stokum JA, Ahmed AK, et al. Surgical Decompression of Traumatic Cervical Spinal Cord Injury: A Pilot Study Comparing Real-Time Intraoperative Ultrasound After Laminectomy With Postoperative MRI and CT Myelography. *Neurosurgery*. Feb 1 2023;92(2):353-362. doi:10.1227/neu.0000000000002207

51. Aarabi B, Chixiang C, Simard JM, et al. Proposal of a Management Algorithm to Predict the Need for Expansion Duraplasty in American Spinal Injury Association Impairment

For Peer Review

Scale Grades A-C Traumatic Cervical Spinal Cord Injury Patients. *J Neurotrauma*. Dec 2022;39(23-24):1716-1726. doi:10.1089/neu.2022.0218

52. Cheung A, Tu L, Manouchehri N, et al. Continuous Optical Monitoring of Spinal Cord Oxygenation and Hemodynamics during the First Seven Days Post-Injury in a Porcine Model of Acute Spinal Cord Injury. *Journal of neurotrauma*. Aug 17 2020;doi:10.1089/neu.2020.7086

53. Shadgan B, Macnab A, Fong A, et al. Optical Assessment of Spinal Cord Tissue Oxygenation Using a Miniaturized Near Infrared Spectroscopy Sensor. *Journal of neurotrauma*. Jun 17 2019;doi:10.1089/neu.2018.6208

For Peer Review

Table 1. Studies reporting on the association between neurological improvement and MAP targets: *Is there a lower limit?*

Author (Year)	Lower Limit Studied	Outcome Measure	Duration of Therapy	Results	Conclusions
Catapano et al (2016)	85 mmHg. Also examined 80 MAP thresholds between 40 and 120 mmHg	Improvement in AIS grade "before discharge", at ~10-40 days post-injury	3 days	-In the AIS A, B/C groups, there was a significantly greater proportion of MAP values below 85 mmHg in patients who did not improve compared to those who did. Average MAPs in patients who improved were 96.6 and 97.2 mmHg in the AIS A and B/C groups, respectively.<br>-In the AIS D group, patients who improved interestingly had a greater proportion of MAP values <85 mmHg threshold than those who did not improve.	-MAP below 85 mmHg may result in worse neurological outcomes in AIS A, B and C patients.
Chen et al (2017)	85-95 mmHg	Improvement in AIS grade at 2 weeks post-injury	Not specified	-There was no correlation between deviation from a MAP goal of 85-95 mmHg and improving on AIS grade.<br>-However, a greater percentage of patients improved if they deviated above the MAP goal of 85-95mmHg.<br>-This study utilized a subdural pressure probe at the injury site to measure SCPP.	-MAP above 85-95 mmHg may result in improved neurological outcomes
Cohn et al (2010)	65, 70, 75, 80, 85 mmHg	Improvement in ASIA Motor Score during acute admission, at ~22 days post-injury	7 days	-The percent of time spent with MAP <65 or <70 mmHg was inversely correlated to improvement in ASIA motor score.<br>-After accounting for confounders, the percent of time a patient spent with MAP <65 or <70 mmHg accounted for 47% and 30% of variability in motor score gains, respectively.	-MAP below 65 or 70 mmHg may result in worse neurological outcomes.
Dakson et al (2017)	85 mmHg	Improvement in AIS grade and ASIA Motor Score as far out as "discharge from rehab", at ~252 days post-injury	5 days	-Patients who failed to meet a MAP goal ≥85 mmHg for a >2-hour period were less likely to improve on the AIS grade (11%) compared to patients whose MAP was consistently ≥85 mmHg (57%).<br>-The odds of not achieving neurologic improvement was 11.1 (95% CI 1.6 to 75.6) times higher in patients with a MAP <85 mmHg.<br>-ASIA motor score improvement was significantly higher in patients who achieved a MAP goal ≥ 85mmHg (22.5±30.9) than those who did not (3.1±23.2).	-MAP below 85 mmHg may result in worse neurological outcomes.
Ehsanian et al (2020)	50-64, 70-94, 95-104 mmHg	Improvement in ASIA Motor Score at "discharge from acute rehab", at ~42-52 days post-injury	Intraoperative	-Increased time spent with MAP between 70 and 94 mmHg was associated with motor score improvement. Individuals with improvement in ASIA motor score tended to spend less time with MAP outside of 70-94 mmHg than patients who did not improve (not statistically significant, p=0.062).<br>-Increased time spent with MAP between 50 and 69 mmHg was associated with worse motor score improvement (not statistically significant)	-A greater time spent with MAP between 70 and 94 mmHg is associated with improvement in neurological outcomes.<br>-A MAP between 50-69 mmHg may be associated with worse neurological outcomes.
Haldrup et al (2020)	80 mmHg	Improvement in AIS grade at 1-year post-injury	Prehospital, intraoperative, 1-2 days, and 3-7 days	-Maintaining a MAP of 80mmHg in the prehospital period, intraoperatively and the first 2 days in the ICU was significantly correlated to long-term neurological outcomes. Patients with a MAP ≥80 mmHg, with none or few events below 80mmHg had significantly better AIS.	-MAP below 80 mmHg may result in worse neurological outcomes.

For Peer Review

Hawryluk et al (2015)	85 mmHg. Also examined 80 MAP thresholds between 40 and 120 mmHg	Improvement in AIS grade before discharge, at ~27-47 days post-injury	5 days	-Patients who improved by >1 AIS grade had the highest MAP and the lowest proportion of measures below 85 mmHg at every examined time point (first 3, first 7 and first 30 days after injury).<br>-Patients who improved by 1 AIS grade had higher MAPs for the first 24-72 hours than patients who did not improve.<br>-Patients who improved by >1 AIS grade had a lower number of measures below all examined MAP thresholds compared to patients who did not improve (especially in the first 24 hours).<br>-The lowest MAP at which patients with no improvement were distinguished from those with 1 grade improvement was 70-75 mmHg	-MAP below 85 mmHg may result in worse neurological outcomes.<br>-MAP 70-75 mmHg may be the lowest blood pressure threshold associated with neurological benefit.
Hogg et al (2021)	<75 to 94 mmHg	Improvement in ASIA Motor Score, within 1 week post-injury.	Not standardized	-An increase in MAP from <75 to 95 mmHg was associated with an average gain of 4 motor points.<br>-This study utilized a subdural pressure probe at the injury site to measure SCPP.	-MAP below 75 mmHg may be associated with worse motor recovery.
Martin et al (2015)	65, 70, 85, 90 mmHg	Improvement in ASIA Motor Score "during hospitalization", at ~18 days post-injury	3 days	-Patients with >60 episodes of relative hypotension (<90, <85 mmHg) during the first 3 days admission also had worse admission motor scores than those with fewer episodes of relative hypotension. This difference was not significant when exploring MAPs of <70 or <65 mmHg.<br>-An increased frequency of hypotensive events during hospitalization was not associated with change in motor scores for any MAP threshold.	-Frequency of relative episodes of hypotension are related to more severe SCI.<br>-Episodes of hypotension throughout hospitalization were not associated with change in motor scores.
Park et al (2017)	>85, 75-84, <75 mmHg	Improvement in AIS grade at 3 months post-injury	7 days	-Patients with MAP <75 mmHg on admission were less likely to improve by ≥1 grade on the AIS grade (OR 0.12, 95% CI 0.03 to 0.53) compared to those with MAP ≥85 mmHg.<br>-Maintaining MAPs ≥85 mmHg for 7 days was not associated with improvement in AIS grade compared to achieving MAPs of 75-84 or <75 mmHg.	-MAP below 75 mmHg on admission may result in worse neurological outcomes
Saadoun et al (2017)	No cut-off specified	Improvement in AIS grade at 9-12 months post-injury	7 days	-MAP was not significantly associated with improvement in AIS grade. A greater percentage of patients demonstrated improvements if SCPP (as measured with a subdural pressure probe at the injury site) was maintained over 90mmHg (MAP must be higher than this).	-No cut-off
Sewell et al (2019)	<80, <70 mmHg	Improvement in AIS grade at 30 days post-injury	24 hours	-There was no association between change in AIS grade and the presence of MAP readings <80 or <70 mmHg.	-No cut-off
Squair et al (2017)	80-85, <70mmHg	Improvement in AIS grade and ASIA Motor Score at 6 months post-injury	5 days	-Patients were more likely to demonstrate an improvement on both AIS grade (OR 1.037, 95% CI 1.011 to 1.063) and motor score (OR 1.038, 95% CI 1.013 to 1.064) if they maintained a MAP goal between 80 and 85 mmHg for 5 days.<br>-Further analysis demonstrated that patients who failed to demonstrate improvement in AIS grade or motor score did not have more episodes of MAP <70 mmHg (AIS grade: OR 1, 95% CI 0.98 to 1.1; AMS: OR 0.16, 95% 0.36 to 0.69)<br>-This study utilized a subdural pressure probe at the injury site to measure SCPP.	-MAP between 80 and 85 mmHg may result in improved neurological outcomes.

For Peer Review

Weinberg et al (2021)	85 mmHg	Improvement in AIS grade "by hospital discharge", at ~17 days post-injury	3 days	-The proportion of MAPs ≥85mmHg was significantly higher in patients that demonstrated improvement of AIS grade than those who did not (81% versus 72%). Based on multivariate analysis, each 10% increase in the proportion of MAPs ≥85 mmHg was associated with a 17% increased likelihood of improving on AIS grade (HR 1.17, 95% CI 1.03 to 1.42).	-MAP above 85 mmHg may result in improved neurological outcomes.

For Peer Review

Table 2. Studies reporting on the association between neurological improvement and MAP targets: *Is there an upper limit?*

Author (Year)	Upper Limit Studied	Outcome Measure to Assess Change in Neurological Status	Duration of Therapy	Results	Conclusions
Ehsanian et al (2020)	95-104mmHg	Improvement in ASIA Motor Score at "discharge from acute rehab", at ~42-52 days post-injury	Intraoperative	-Increased time spent with MAP between 70 and 94 mmHg was associated with motor score improvement. Motor scores increased 0.036 for each minute of exposure to the MAP range 70–94 mmHg during the operative procedure (statistically significant p = 0.042). Motor scores decreases −0.039 for each minute of exposure to the MAP range 95–104 mmHg (not statistically significant p = 0.764).	-A greater time spent with MAP between 70 and 94 mmHg is associated with improvement in neurological outcomes.<br>-A MAP between 95–104 mmHg may be associated with worse neurological outcomes.
Hawryluk et al (2015)	Examined 80 different and physiologically relevant MAP thresholds (40-120mmHg)	Improvement in AIS grade before discharge, at ~27-47 days post-injury	5 days	-MAP above 95mmHg is not related to neurological improvement.	-MAP above 95mmHg may not have benefit on neurological outcomes.
Hogg et al (2021)	95-130mmHg	Improvement in ASIA Motor Score while measuring MAP & SCPP changes within 1 week post-injury	Not standardized	-Fluctuations in MAP in the range between 95-130mmHg were not associated with changes in ASIA motor scores	-MAP between 95 and 130mmHg may not have benefit on neurological outcomes. Improvement in motor scores levels off at 95mmHg.

For Peer Review

For Peer Review

Table 3. Studies reporting on adverse events: ***Is there an upper limit for MAP above which risk of adverse events increase?***

Author (Year)	MAP Goal	Adverse Event	Duration of MAP Therapy	Results	Conclusions
Inoue et al (2014)	None	Tachycardia (>130bpm), bradycardia (<50bpm), new atrial fibrillation, ventricular tachycardia, elevated troponin, ST changes consistent with ischemia, acidosis (pH<7.0), atrial flutter and skin necrosis.	5 days	-Overall, 74.0% of patients treated with any type of vasopressor experienced a complication. The most common type of complication was tachycardia (41.2%), followed by bradycardia (24.4%), atrial fibrillation (12.2%), ventricular tachycardia (10.7%), elevated troponin/ST changes (6.1%), acidosis (3.1%), atrial flutter (0.78%) and skin necrosis (0.78%).<br>-In multivariate analysis, both the use of dopamine and phenylephrine were associated with an increased rate of complications.	-Vasopressor use was associated with a high rate of complications (74.0%).
Readdy et al (2015)	>85mmHg	Tachycardia (>130bpm), bradycardia (<50bpm), new atrial fibrillation, ventricular tachycardia, elevated troponin.	4 days	-All patients required vasopressor support to maintain MAP>85mmHg. 91% of patients on vasopressors experienced a complication and 9% had multiple complications: 15% atrial fibrillation, 35% tachycardia (>130bpm), 32% bradycardia (<50bpm), 9% ventricular tachycardia, 9% elevated troponin.	-Vasopressor use was associated with a high rate of complications (91%),
Readdy et al (2016)	>85mmHg	Tachycardia (>130bpm), bradycardia (<50bpm), new atrial fibrillation, ventricular tachycardia, elevated troponin.	4 days	-All patients required vasopressor support to maintain MAP >85mmHg. 94% of patients on vasopressors experienced a complication. 62% of patients had a cardiogenic adverse event.	-Vasopressor use was associated with a high rate of complications (94.0%), especially cardiogenic (62%).
Vale et al (1997)	>85mmHg	Hypertensive hemorrhage, stroke, myocardial infarction or death	7 days	-There were no recognized untoward effects of aggressive volume resuscitation or MAP elevation therapy. Specifically, there was no instance of hypertensive hemorrhage, stroke, myocardial injury or death.	-Maintaining MAP above 85mmHg resulted in no serious adverse events.
Weinberg et al (2021)	>85mmHg	Pneumonia, DVT, unplanned return to the OR, unplanned intubation, superficial surgical site infection	3 days	-Vasopressors were administered to 78% of patients. There was no significant difference in the average proportion of elevated MAP by complication status (pneumonia, DVT, unplanned return to the OR, unplanned intubation and superficial surgical site infection). Complications were more prevalent in patients who required vasopressors (28.3%) than those who did not (10.0%, OR 3.6, 95% CI 1.0 to 12.6).	-Maintaining MAP above 85mmHg was not associated with an increase in adverse events. Vasopressor use, however, was associated with an increased rate of adverse events.

For Peer Review

Table 4. Studies reporting on different durations of MAP-targeted therapy

Author (Year)	MAP Goals	Outcome Measure	Duration of Therapy	Results
Catapano et al (2016)	85 mmHg.<br>Also examined 80 MAP thresholds between 40 and 120 mmHg	Improvement in AIS grade "before discharge", at ~10-40d post-injury	3 days	-In the AIS A, B/C groups, MAPs were significantly higher for 3 days in patients who improved on ASIA Impairment Score compared to those who did not.
Chen et al (2017)	85-95 mmHg	Improvement in AIS grade at 2 weeks post-injury	Not specified	-Did not specify duration of therapy
Cohn et al (2010)	65mmHg<br>70mmHg<br>75mmHg<br>80mmHg<br>85mmHg	Improvement in ASIA Motor Score	7 days	-The percent of time spent with MAP <65 or <70mmHg during a 7 day period was inversely correlated to improvement in ASIA motor score.
Dakson et al (2017)	85mmHg	Improvement in ASIA Motor Score during acute admission, at ~22 days post-injury	5 days	-Patients who failed to meet a MAP goal ≥85mmHg for a >2-hour period during the first 5 days were less likely to improve on the AIS grade (11%) compared with patients with MAP ≥85mmHg (57%).
Ehsanian et al (2020)	50-64mmHg<br>70-94mmHg<br>95-104mmHg	Improvement in ASIA Motor Score at "discharge from acute rehab", at ~40-50 days post-injury	Intraoperative	-Increased time spent with MAP between 70 and 94mmHg during surgery was positively associated with ASIA motor score improvement.
Haldrup et al (2020)	80mmHg	Improvement in AIS grade at 1-year post-injury	Prehospital, intraoperative, 1-2 days, and 3-7 days	-Maintaining MAP goals during prehospital transport, intraoperative and for the first 1 to 2 days in the ICU had a moderate impact on neurological outcomes.<br>-Maintaining MAP goals on day 3 to 7 was not significantly associated with improved neurological outcomes
Hawryluk et al (2015)	85mmHg<br>80 different and physiologically relevant MAP thresholds (40-120mmHg)	Improvement in AIS grade before discharge, at ~27-47 days post-injury	5 days	-The group achieving >1 AIS grade improvement had the highest MAP and the lowest proportion of measures below 85mmHg at every examined time point (first 3, first 7 and first 30 days after injury).<br>-A higher MAP correlated with outcome in the first 3 days after injury; however, by 7 days, higher MAP values were only noted in the group achieving >1 AIS grade improvement.<br>-The group achieving >1 grade of improvement on AIS had a reduced number of measures below all examined MAP thresholds compared to groups with less improvement, especially in the first 24 hours.
Hogg et al (2021)	<75mmHg to 94mmHg	Improvement in ASIA Motor Score, at ~6 months post-injury	Not standardized	-Duration of therapy was not standardized.

For Peer Review

Martin et al (2015)	65mmHg<br>70mmHg<br>85mmHg<br>90mmHg	Improvement in ASIA Motor Score “during hospitalization”, at ~18 days post-injury	3 days	-Patients with >60 episodes of relative hypotension (<90, <85mmHg) had worse admission motor scores than those with fewer episodes. This difference was not significant when exploring MAPs of <70 or <65mmHg. In contrast, increased frequency of hypotensive events was not associated with change in motor scores during hospitalization for any MAP threshold.
Park et al (2017)	>85mmHg<br>75-84mmHg<br><75mmHg	Improvement in AIS grade at 3 months post-injury	7 days	-Compared to MAP ≥85mmHg, patients with MAP <75mmgHg on admission were less likely to improve by ≥1 grade on the ASIA Impairment Score (OR 0.12, 95% CI 0.03 to 0.53).<br>-MAPs ≥85mmHg for 7 days was not significantly associated with improvement of AIS grade compared to MAPs of 75-84mmHg or <75mmHg.
Saadoun et al (2017)	No cutoff specified	Improvement in AIS grade at 9-12 months post-injury	7 days	-A greater percentage of patients demonstrated improvements if SCPP was maintained over 90mmHg (MAP must be higher than this) for 7 days.
Sewell et al (2019)	<80mmHg<br><70mHg	Improvement in AIS grade at 30 days post-injury	24 hours	- There was no association between change in ASIA Impairment Score and the presence of MAP readings <80mmHg or <70mmHg for the first 24 hours.
Squair et al (2017)	80-85mmHg<br><70mmHg	Improvement in AIS grade and ASIA Motor Score at 6 months post-injury	5 days	-Did not evaluate different durations of MAP therapy. However, increased exposure to SCPP <50mmHg (and therefore lower MAP) in the first 48 hours reduced the likelihood of converting AIS grade and achieving motor improvement by greater than or equal to 6 points.
Weinberg et al (2021)	85mmHg	Improvement in AIS grade “by hospital discharge”, at ~7-15 days post-injury	3 days	-The proportion of MAP ≥85mmHg was significantly higher for the first 3 days in patients that demonstrated improvement of AIS grade than those who did not (81% versus 72%).

For Peer Review

For Peer Review

Table 5. Studies reporting on the use of different vasopressors

Author (Year)	MAP Goal	Vasopressor	Results
Inoue et al (2014)	None	Dopamine, phenylephrine	- Dopamine was associated with significantly more complications (69.2%) than phenylephrine (46.5%). Patients who were treated with dopamine had a higher rate of sinus tachycardia, ventricular tachycardia, ST segment elevation, troponinemia and atrial fibrillation. In contrast, phenylephrine use was associated with a higher rate of metabolic acidosis and bradycardia
Readdy et al (2015)	>85mmHg	Dopamine, phenylephrine	- There was a nonsignificant trend toward a higher complication rate with dopamine (68%) compared to phenylephrine (45%)
Readdy et al (2016)	>85mmHg	Dopamine, phenylephrine	-Rates of complications were significantly greater with dopamine (76%) than phenylephrine (40%)

For Peer Review
